# Influence of Spacer Chains Derived from Ring-Opened Crown Ethers on the Anti-Influenza A Virus Activity of *closo*-Decaborates Bearing Amino Acid Ester Pendant Moieties

**DOI:** 10.3390/molecules31183157

**Published:** 2026-09-08

**Authors:** Evgenii Yu Matveev, Elizaveta E. Rasskazova, Elizaveta A. Eshtukova-Shcheglova, Artemiy I. Nichugovskiy, Timur M. Garaev, Ilya I. Yudin, Natalya V. Breslav, Tatyana V. Grebennikova, Elena I. Burtseva, Varvara V. Avdeeva, Konstantin Yu Zhizhin, Nikolai T. Kuznetsov

**Affiliations:** 1 Institute of Fine Chemical Technologies, MIREA—Russian Technological University, Vernadskogo pr. 86, Moscow 119571, Russia; cat1983@yandex.com (E.Y.M.); l.e.rasskazova@mail.ru (E.E.R.); shchegs.ea@gmail.com (E.A.E.-S.); ainichugovskiy@gmail.com (A.I.N.); zhizhin@igic.ras.ru (K.Y.Z.); 2 Gamaleya National Research Center for Epidemiology and Microbiology, Ministry of Health of Russian Federation, Moscow 123098, Russia; tmgaraev@gmail.com (T.M.G.); yudinilya2000@yandex.ru (I.I.Y.); n.belyakova1983@gmail.com (N.V.B.); t_grebennikova@mail.ru (T.V.G.); elena-burtseva@yandex.ru (E.I.B.); 3 Kurnakov Institute of General and Inorganic Chemistry, Russian Academy of Sciences, Moscow 119991, Russia; ntkuz@igic.ras.ru

**Keywords:** antiviral activity, viroporins, amino acids, polyhedral boron anions, *closo*-decaborate anion, crown-ethers, opening of cyclic substituents

## Abstract

A series of novel substituted derivatives of the *closo*-decaborate anion [B_10_H_10_]^2−^ bearing alkoxy spacer chains of varying length derived from ring-opened crown ethers (12-crown-4, 15-crown-5, and 18-crown-6) with pendant L-amino acid methyl ester residues (L-tryptophan and L-histidine) have been synthesized. The synthetic approach involved the nucleophilic ring-opening of oxonium crown ether derivatives of the *closo*-decaborate anion followed by coupling with amino acid methyl esters via mixed anhydride activation. The obtained compounds were characterized by multinuclear NMR spectroscopy (^1^H, ^11^B, ^13^C), IR spectroscopy, elemental analysis, and electrospray ionization mass spectrometry (ESI-MS). The compounds were obtained as sodium salts and evaluated for their in vitro cytotoxic and antiviral properties against the influenza A virus strain A/Moscow/78/2020 (H1N1)pdm09, which contains the S31N mutation conferring resistance to adamantane-class drugs. Cytotoxicity was assessed on MDCK cells, and antiviral activity was determined by cell ELISA. The results revealed a clear structure–activity relationship: the length of the alkoxy spacer chain significantly influenced both antiviral activity and selectivity. The shortest spacer (derived from 12-crown-4) proved to be optimal, while elongation of the chain led to a decrease in antiviral potency. The compound containing a 12-crown-4-derived spacer and a tryptophan methyl ester residue exhibited the highest selectivity index (SI = 155) with low cytotoxicity (CC_50_ = 155 µg/mL) and high antiviral activity (IC_50_ = 1.0 µg/mL). Tryptophan-containing derivatives consistently outperformed their histidine analogues, confirming the key role of the indole side chain in antiviral activity. Overall, the *closo*-decaborate platform with crown ether-derived spacers and amino acid ester pendant groups represents a promising scaffold for the development of low-toxicity, highly selective inhibitors of influenza A virus replication. The compound with the 12-crown-4-derived spacer and a tryptophan methyl ester residue merits further investigation as the most promising among the synthesized samples.

## 1. Introduction

Vaccination remains the most accessible solution for the treatment and prevention of diseases caused by the influenza virus. However, currently available influenza vaccines have a number of limitations that reduce their effectiveness compared to immunization against other respiratory pathogens [1].

Development of antiviral drugs capable of combating infections caused by multiple viruses is critically needed, as underscored by the coronavirus disease 2019 (COVID-19) pandemic caused by severe acute respiratory syndrome coronavirus 2 (SARS-CoV-2) and the ongoing annual influenza epidemics. In this context, boron clusters—a class of inorganic polyhedral compounds featuring a unique three-dimensional architecture and high thermal and chemical stability—are attracting attention as a non-traditional platform for the design of antiviral agents.

Boron cluster anions [B*_n_*H*_n_*]^2−^ (*n* = 10, 12) represent unique inorganic systems possessing three-dimensional aromaticity [2,3,4,5]. The exo-polyhedral hydrogen atoms in these systems can be substituted with a variety of C-, N-, O-, S-, and other functional groups [6,7,8,9,10,11,12]. Further functionalization of the already introduced groups is also of great importance. One of the most convenient approaches is the nucleophilic ring-opening of attached cyclic ether molecules to form *closo*-borates containing pendant functional groups, including biologically active ones [13,14,15,16,17,18,19,20,21,22,23,24,25].

One example of the successful application of boron clusters in antiviral chemistry is the development of HIV protease (HIV PR) inhibitors—a primary target for antiretroviral therapy. Using a structure-based approach, substituted metallacarboranes (bis(dicarbollide) cobalt complexes) were identified and characterized as a novel class of HIV PR inhibitors. Linking two clusters via a linker bearing a substituted ammonium group afforded a series of compounds based on the general formula Na[H_2_N–(8–(C_2_H_4_O)_2_–1,2-C_2_B_9_H_10_)(1′,2′-C_2_B_9_H_11_)–3,3′-Co)_2_]. The study of the inhibitory properties of these compounds with various substituents, determination of the crystal structure of the HIV PR–inhibitor complex, and computational analysis of the conformational space of the linker demonstrated that linker-substituted cobalt bis(dicarbollides) can serve as lead compounds for the development of potent HIV protease inhibitors, including those active against drug-resistant mutants [26,27].

Another promising direction is the design of polyfunctionalized dodecaborate clusters [B_12_(OR)_12_]^2−^, the surface of which is densely covered with charged ligands. These compounds exhibit minimal cytotoxicity toward mammalian cells and promising antiviral activity against both human immunodeficiency virus type 1 (HIV-1) and cytomegalovirus (CMV). This class of compounds represents a promising platform for studying the role of ligand identity, molecular size, and charge density in antiviral activity [28]. Therefore, the development of low-toxicity and effective small-molecule inhibitors of viral replication, particularly against influenza virus, is of crucial importance for the prevention and treatment of circulating and emerging strains.

**Figure 1:**
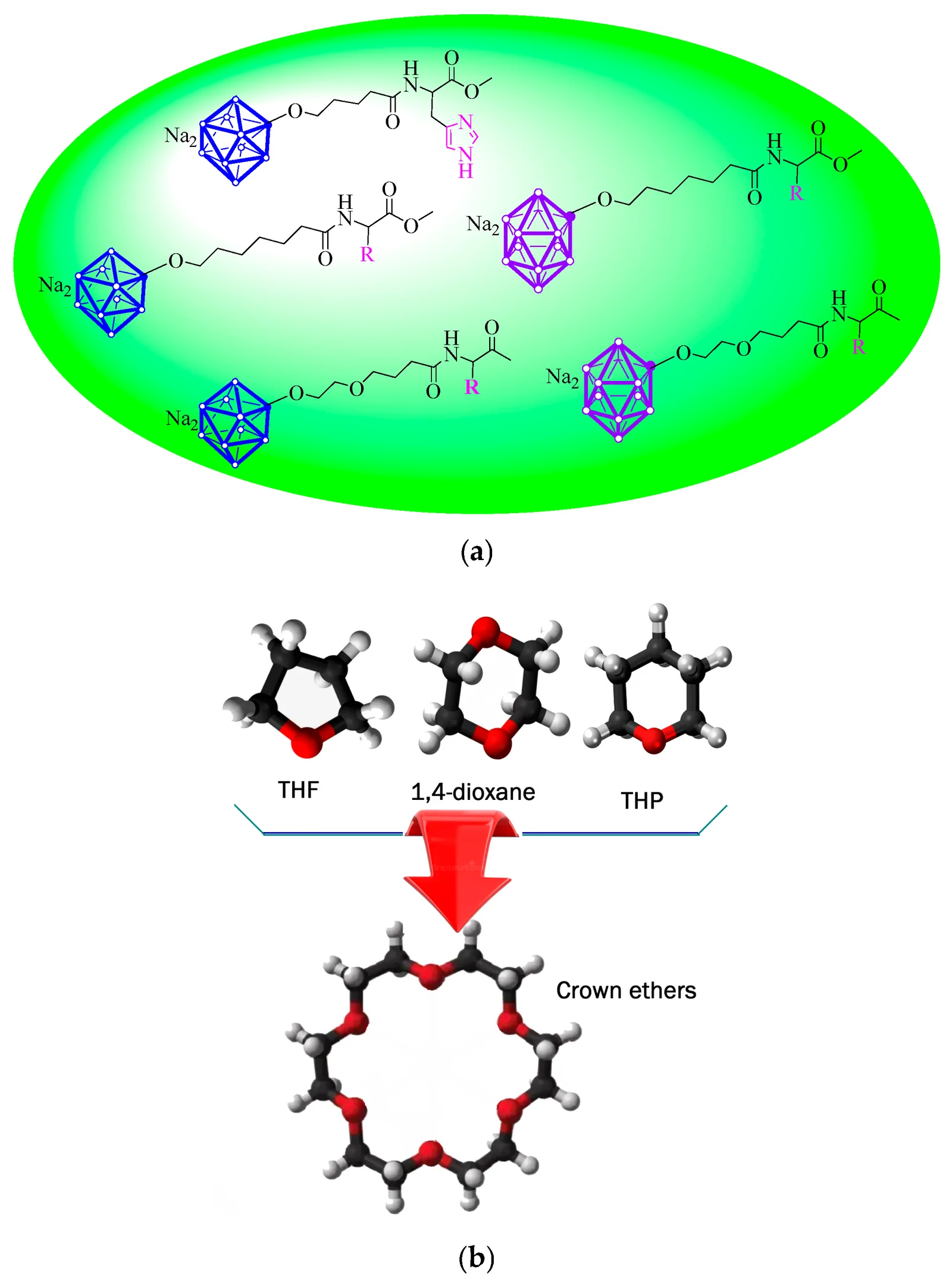
(**a**) Structures of known B_10_-based (blue) and B_12_-based (purple) compounds with amino acid residues (tryptophan, histidine) found to be active against A/IIV-Orenburg/83/2012 (H1N1)pdm09 [29,30,31,32,33]; (**b**) oxygen-containing rings capable of serving as synthetic precursors of spacer groups.

Previously, we proposed, using low-molecular-weight bioinorganic platforms based on polyhedral anions [B*_n_*H*_n_*]^2−^ (*n* = 10, 12) and residues of L-amino acid methyl esters (His, Trp, Met), new inhibitors of the replication of modern influenza A virus strains resistant to existing drugs (Figure 1a). The design of the bioinorganic agents included a membranotropic core of the *closo*-decaborate/*closo*-dodecaborate anion and a spacer group based on ring-opened oxonium-type substituents—molecules of 1,4-dioxane, tetrahydropyran, and tetrahydrofuran [29,30,31,32,33]. However, the alkoxy spacer chain can be lengthened by using bulkier cyclic ethers, such as crown ethers (Figure 1b).

**Figure 2:**
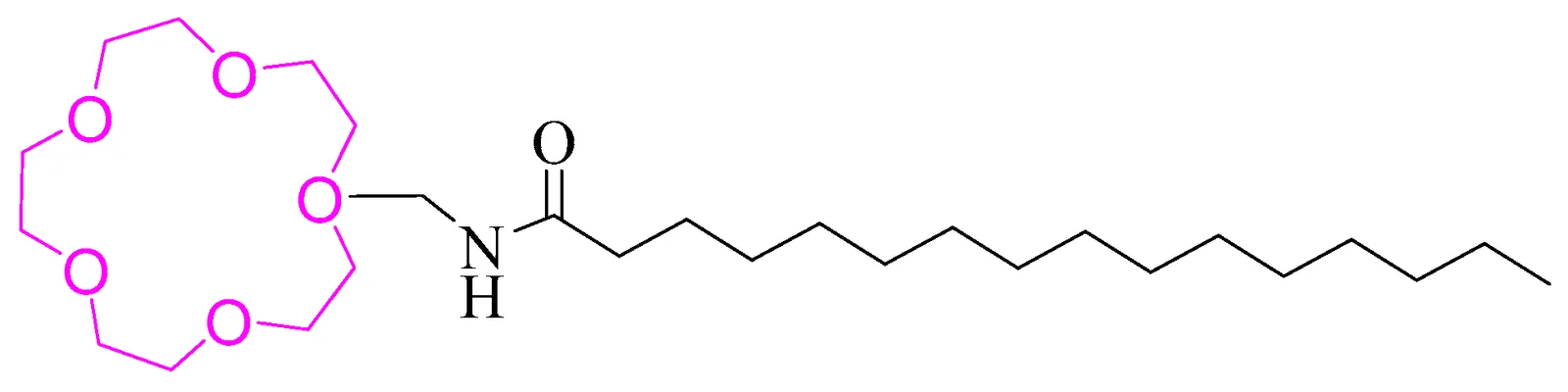
Structure of *N*-hexadecanoyl-2-aminomethyl-15-crown-5.

The unique structure of crown ethers has attracted the attention of many scientists to the use of these compounds in organic synthesis and drug delivery. Crown ethers, with their unique structure, hold great potential in the drug delivery process [34,35,36,37]. An interesting feature of crown ethers is that the lone electron pairs present on the heteroatoms of the ring allow the molecule to form complexes with a wide range of cations within the empty cavity located in the center of the ring [38]. The formation of complexes between crown ethers and cations depends on the relative size of the crown ether compared to the metal ion, the nature of the solvent, and the nature of the substituents on the crown ether. Vesicular drug delivery systems, such as nonionic liposomes (niosomes), are playing an increasingly important role, as they can be used as targeted drug delivery systems. Niosomes are capable of encapsulating both hydrophilic and lipophilic drugs and can serve as effective drug carriers [39,40,41]. A known crown ether derivative is the amphiphilic N-hexadecanoyl-2-aminomethyl-15-crown-5 [42] (Figure 2). PCE was used as a cation-containing or cation-sensitive agent for a controlled-release system. That is, crown-5 acts as a targeted courier for the N-hexadecanoyl-2-aminomethyl molecule.

It should be emphasized that crown ethers themselves (including 12-crown-4, 15-crown-5, and 18-crown-6) are not regarded as direct antiviral agents; rather, they serve as macrocyclic scaffolds for the design of more potent derivatives [43,44,45,46,47].

In the present work, an alternative use of crown ethers was proposed, namely derivatives of the *closo*-decaborate anion with molecules of 12-crown-4, 15-crown-5, and 18-crown-6 as exo-polyhedral substituents, which were subsequently subjected to nucleophilic ring-opening reactions of the oxonium cycles. As a result, *closo*-decaborates bearing attached residues of L-amino acid methyl esters (His and Trp) as pendant groups were obtained. Structurally related compounds we previously proposed, containing ring-opened tetrahydrofuran, 1,4-dioxane, and tetrahydropyran cycles as spacers [29,30,31,32,33], featured an alkoxy chain length of 5–6 atoms in the chain. In the present work, the goal was to significantly increase the length of the alkoxy chain and to study the effect of this modification in such bioinorganic molecules on their antiviral and cytotoxic properties.

Investigating the changes in biological properties with increasing spacer length was a key objective of this study, and therefore the biological evaluation of the intermediates was not carried out. It is also worth noting the laboriousness of this additional task, since all intermediates are tetrabutylammonium salts, which are highly toxic to the MDCK cell monolayer [29]. Conducting such a study would require the conversion of each intermediate into the corresponding sodium salts, which is extremely time-consuming and impractical. Moreover, our previous studies have shown that aliphatic groups (L-methionine-OMe) or alicyclic moieties (methyl ester or methyl 2-amino-3-(2-oxopyrrolidin-3-yl)propanoate) at the spacer terminus are inferior in antiviral activity to heterocyclic aromatic substituents (L-tryptophan methyl ester, L-histidine methyl ester) [30,31,32], whereas derivatives with pendant groups –NHCH_2_CH_2_NH_2_–, –OOC(o-C_6_H_4_)COOH–, –OOCCH_2_NHCH_2_COOH–, –OCH_2_CH_2_OH– and terminal groups –CN, –SCN, and –SH were found to show weak antiviral activity at high concentrations (1250 μg/mL) [48]. Therefore, heterocyclic His and Trp were chosen for testing the effect of spacer length on the antiviral properties of the final compounds.

The aim of the work was to synthesize new substituted derivatives of the *closo*-decaborate anion Na_2_[2-B_10_H_9_O*_n_*(CH_2_)_2*n*+1_CO-XOMe] (*n* = 4, 5, 6; X = Trp or His), obtained by ring-opening of the crown ether substituent in the anions [2-B_10_H_9_O*_n_*(CH_2_)_2*n*_]**^−^**, and test their antiviral activity against influenza virus A/Moscow/78/2020 (H1N1)pdm09.

## 2. Results and Discussion

### 2.1. Synthesis of Target Compounds Na_2_***4***–Na_2_***9***

The targeted synthesis of a new class of bioinorganic systems based on derivatives of boron cluster anions with chemically bound amino acids makes it possible to obtain compounds that exhibit affinity for viral target proteins and the ability to penetrate cells to inhibit the replication of RNA-containing viruses in humans and animals. Compounds based on boron cluster anions can possess both hydrophobic and hydrophilic properties, since they are anions, and variation in the nature and size of the cation allows the solubility of these substances in solvents of various polarities to be controlled. In particular, potassium/sodium salts of the target derivatives of *closo*-hydroborate anions and amino acids will exhibit significant water solubility. This property is extremely important for membranotropic drugs. This ability is also crucial for the bioavailability of the future drug and its toxicity toward cells and the organism.

**Figure 3:**
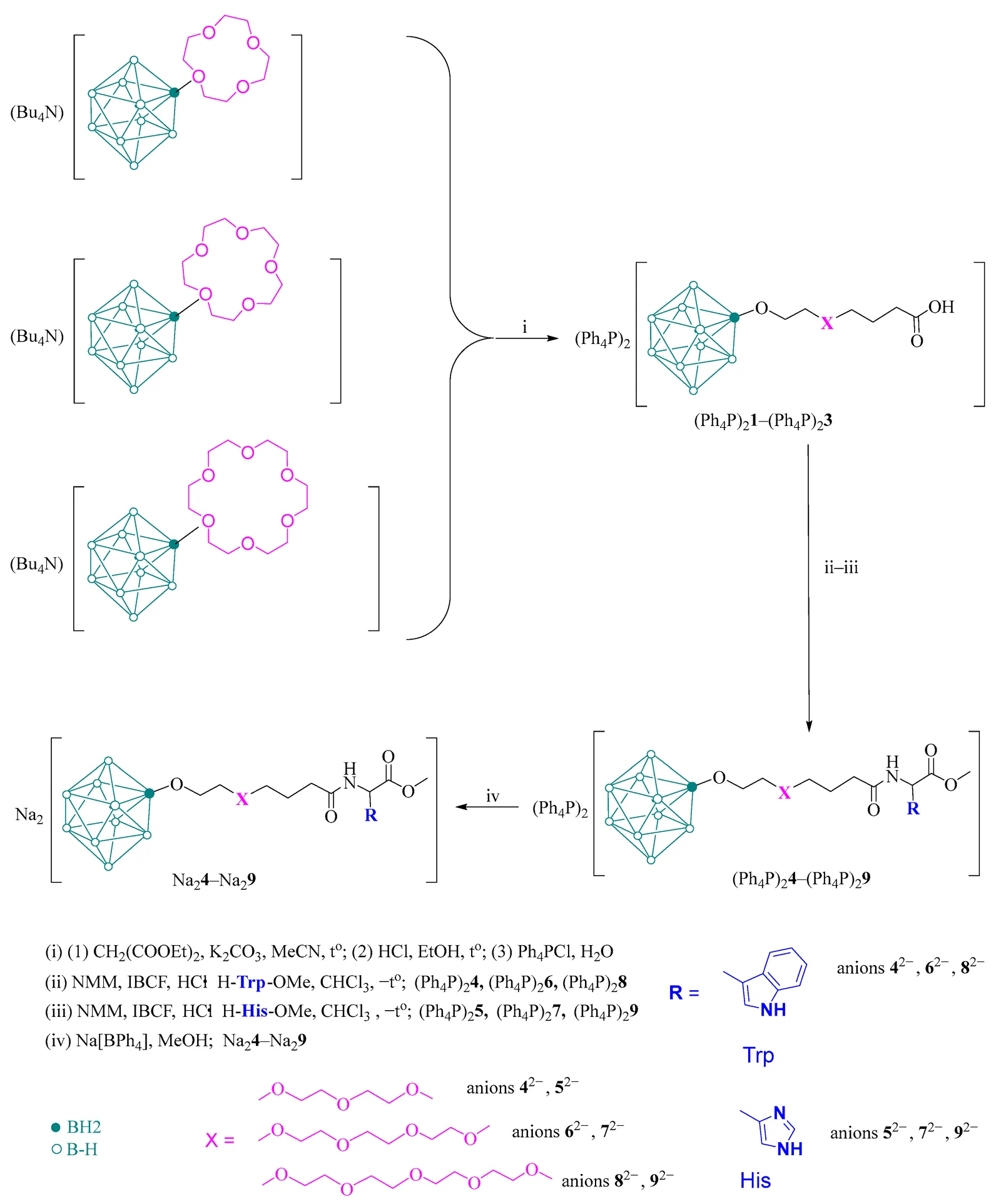
Synthetic scheme for the preparation of [B_10_H_10_]^2–^ derivatives with pendant groups being amino acid ester residues attached to the boron cluster via an alkoxy spacer chain.

In this study, monosubstituted derivatives of the *closo*-decaborate anion with various crown ethers—(*n*-Bu_4_N)[2-B_10_H_9_O_4_(CH_2_)_8_], (*n*-Bu_4_N)[2-B_10_H_9_O_5_(CH_2_)_10_], and (*n*-Bu_4_N)[2-B_10_H_9_O_6_(CH_2_)_12_]—were subjected to nucleophilic ring-opening reactions of the cyclic substituent with malonic ester in the presence of K_2_CO_3_, followed by acidic hydrolysis. The resulting compounds are acids (Ph_4_P)_2_[B_10_H_9_O(CH_2_CH_2_O)*_n_*(CH_2_)_3_COOH] (*n* = 3, 4, 5) (compounds **1**–**3**), in which the carboxyl group is separated by an alkoxy group of varying length (Figure 3, first step). The progress of the ring-opening reactions of the oxonium cycles was monitored by ^11^B{^1^H} NMR spectroscopy. The spectra of the reaction products remained characteristic of monosubstituted *closo*-decaborates; however, due to the change in the nature of the substituent from oxonium to ether, a redistribution of signals was observed: the singlet from the ipso boron atom shifted upfield, and the signals from the apical positions converged (see Supplementary Materials). A redistribution of signals from the remaining equatorial boron atoms was also observed. Studies have shown that the reaction time is closely related to the size of the introduced crown ether, namely, it is inversely proportional to it. In particular, the ring-opening reaction of the attached 18-crown-6 proceeds in approximately 2 h, whereas the analogous reaction with the 12-crown-4 substituent requires more than 16 h.

Subsequently, derivatives with methyl esters of the amino acids L-histidine and L-tryptophan were obtained (Figure 3, second step). These amino acid residues were selected due to their greatest promise as inhibitors of influenza A virus replication, based on previous experience [49] in studying the influence of the amino acid side-chain structure on in vitro antiviral properties. Thus, the presence of aliphatic (linear and cyclic) substituents was significantly inferior to the aromatic heterocyclic side groups of indole and imidazole. Accordingly, pairs of compounds (His and Trp) were obtained for each alkoxide spacer represented by the ring-opened crown ethers –O_4_(CH_2_)_8_–, –O_5_(CH_2_)_10_–, and –O_6_(CH_2_)_12_– (compounds Na_2_**4**–Na_2_**9**).

The spacer chains based on the disclosed cycles of crown ethers attached to the boron cluster –O_4_(CH_2_)_8_–, –O_5_(CH_2_)_10_–, and –O_6_(CH_2_)_12_– constitute a systematic series differing by one ethylene oxide unit (–CH_2_CH_2_O–), which enables structure–activity relationship (SAR) analysis. In addition to length, the increasing number of oxygen atoms (4 → 5 → 6) along the spacer modulates both conformational flexibility and the hydrogen-bonding capacity of the linker, potentially affecting its interaction with water molecules and amino acid residues in the binding site of the target protein (presumably the M2 channel). Therefore, determining the optimal spacer length is essential for maximizing antiviral activity among the series of compounds Na_2_**4**–Na_2_**9**.

### 2.2. Biological Testing of Na_2_***4***–Na_2_***9***

To study the antiviral and cytotoxic properties, stock solutions of the compounds were prepared at a concentration of 1.0 mg/mL in distilled water. A 5.0 mg portion was dissolved in 1.0 mL of distilled water, and then 200.0 µL aliquots were transferred into tubes containing 800.0 µL of water to obtain the stock solution. It was noted that compounds Na_2_**4**–Na_2_**7** exhibited very good water solubility, while compounds Na_2_**8** and Na_2_**9** dissolved completely only after intensive mixing on a Vortex V-3 vibrational shaker (ELMI, Riga, Latvia), and true solutions of the compounds were successfully obtained. The stock solution was then filtered through a 0.22 µm polyethersulfone (PES) membrane filter (FDCELL, Luoyang Fudau Biotech Co., Ltd., Luoyang, China) to prevent contamination of the cell monolayer and to avoid bacterial growth. Further dilutions to the test concentrations were performed in DMEM cell growth medium containing 1% fetal bovine serum supplemented with 100 U/mL penicillin + 100 µg/mL streptomycin and L-glutamine.

### 2.3. Study of the Cytotoxic Properties of Compounds Na_2_***4***–Na_2_***9***

**Figure 4:**
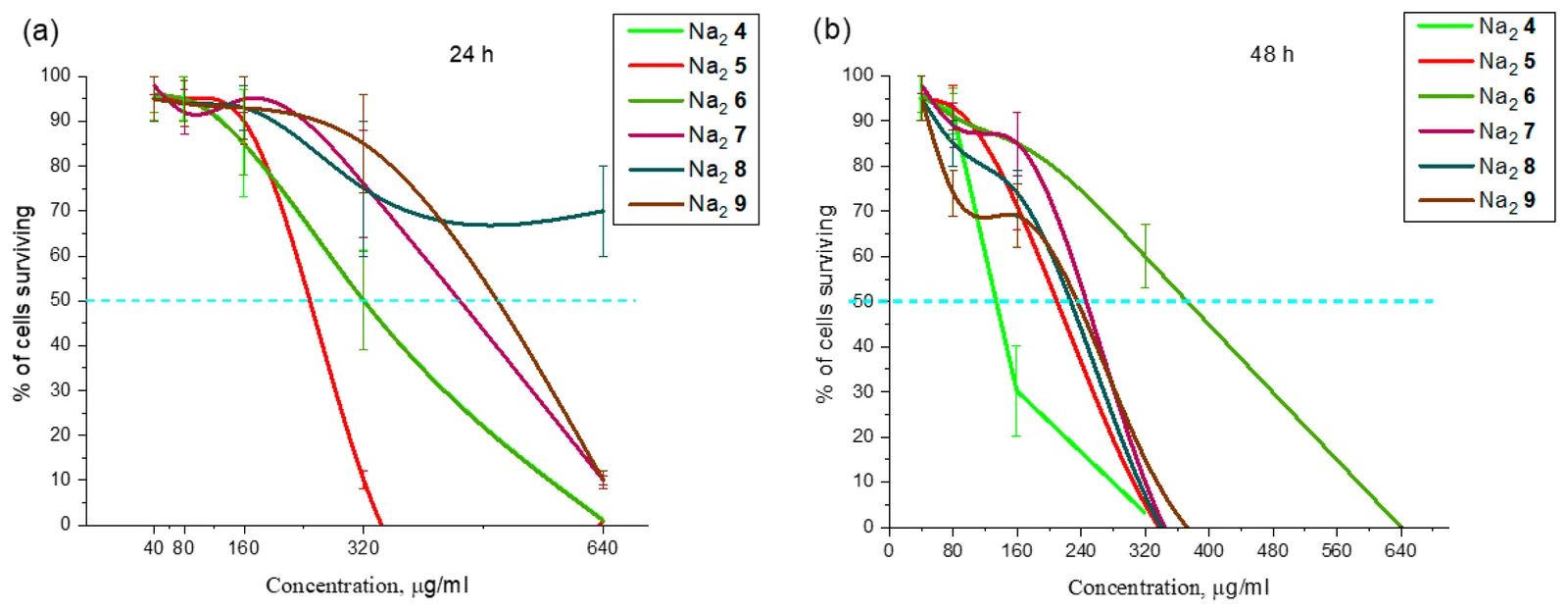
Cytotoxic properties of Na_2_**4**–Na_2_**9**: (**a**) after 24 h; (**b**) after 48 h.

The cytotoxic effect of compounds Na_2_**4**–Na_2_**9** on MDCK cell monolayers was investigated, taking into account the optical density (OD) in wells with various concentrations of the compounds in comparison with the untreated monolayer (without compounds) using a vital dye in the MTT assay after 24 and 48 h of incubation in a 5.0% CO_2_ atmosphere. The following compound concentrations were used: 20.0, 40.0, 80.0, 160.0, 320.0, and 640.0 µg/mL. In the wells containing the compound concentration of 640.0 µg/mL, a yellowish color change in the medium indicator was observed, indicating the slightly acidic nature of the compounds. At the remaining concentrations, the color of the medium indicator remained normal. The evaluation of the cytotoxic effect of various concentrations of compounds Na_2_**4**–Na_2_**9** was performed based on the analysis of cell viability by visual assessment of the cell monolayer state, in comparison with the cell control (without compound). The results are presented in Figure 4.

### 2.4. Study of the Anti-Influenza Properties of Compounds Na_2_***4***–Na_2_***9***

Experiments to determine the percentage of viral replication inhibition by compounds Na_2_**4**–Na_2_**9** were carried out against the influenza virus strain A/Moscow/78/2020 (H1N1)pdm09, following a procedure analogous to that previously conducted for compounds Na_2_[B_10_H_9_O(CH_2_)_6_C(O)X], where X = L-Trp-OMe or L-His-OMe [31]. The compounds were tested at six concentrations: 0.5, 1.0, 2.5, 5.0, 7.5, and 10.0 µg/mL.

**Figure 5:**
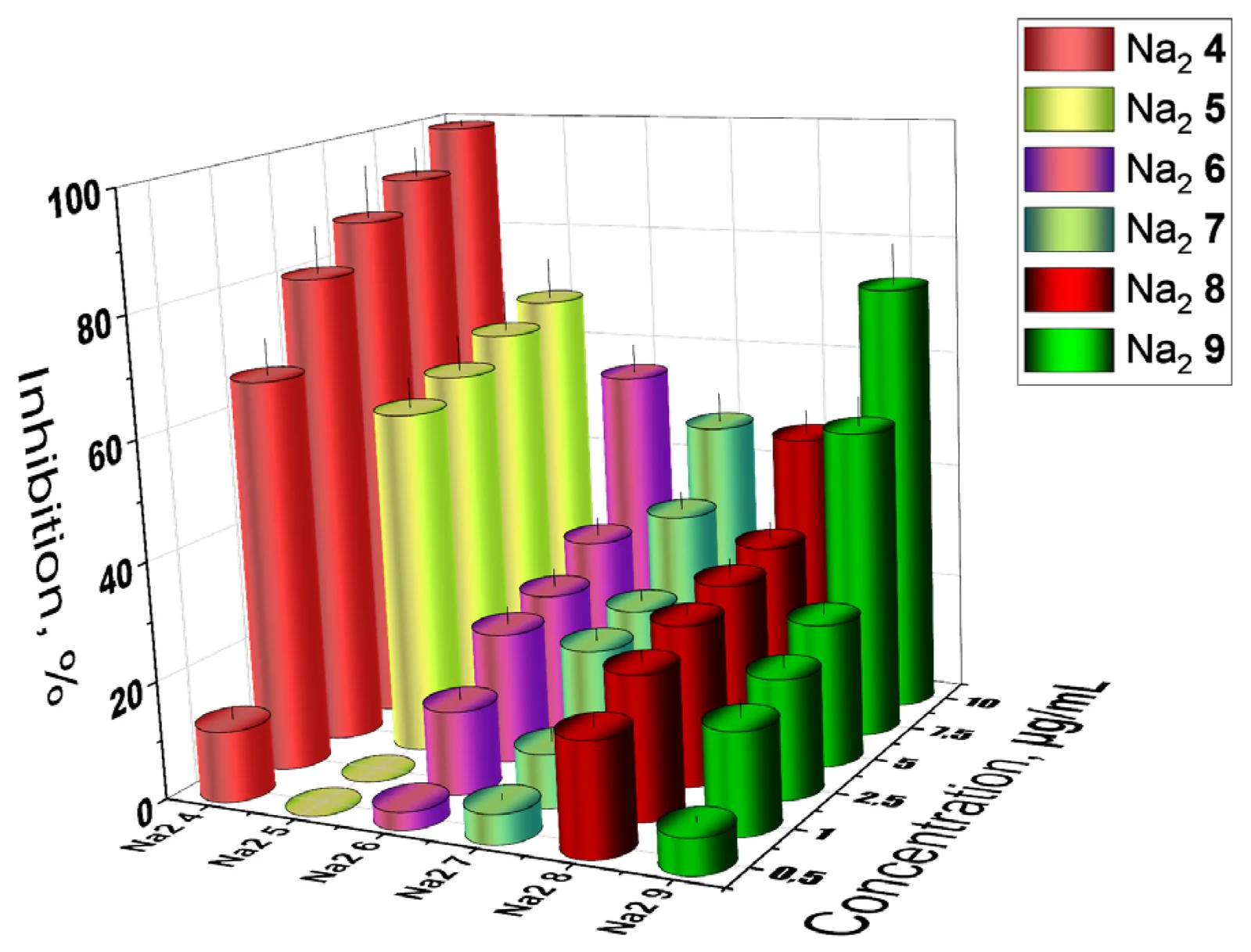
Antiviral properties of novel substituted derivatives of the *closo*-decaborate anion Na_2_**4**–Na_2_**9** against influenza A virus strain A/Moscow/H1N1pdm09.

Figure 5 presents the mean values of antiviral activity results obtained from independent experiments (four replicates in three independent trials) conducted under identical conditions. The results are presented with standard deviation (SD).

The CC_50_ and IC_50_ values were obtained graphically using OriginPro 2021 software (OriginLab Corporation, Northampton, MA, USA). The biological experimental dataset comprised averaged optical density values (OD_450_), from which the percentage of viral replication inhibition and the percentage of cell viability were calculated. Graphical representation of the values with ±SD is shown in Figure 4 and Figure 5. Therefore, standard deviations for the CC_50_ and IC_50_ values cannot be provided, as they were calculated from single averaged OD_450_ values. Details of the biological experimental procedure and calculations are provided in the Supplementary Materials.

The chemotherapeutic index (selectivity index, SI) is defined as the ratio of the cytotoxic concentration (CC_50_) to the antiviral concentration (IC_50_): SI = CC_50_/IC_50_. This parameter is a key criterion for assessing the potential of a compound as an antiviral agent. The higher the SI value, the more selectively the compound acts on the virus without exerting significant toxic effects on host cells. SI values > 10 are generally considered to indicate high selectivity and promise for further preclinical studies.

As follows from the presented data (Table 1), all synthesized compounds Na_2_**4**–Na_2_**9** demonstrated pronounced antiviral activity against the influenza virus strain A/Moscow/78/2020 (H1N1)pdm09, while their cytotoxicity varied depending on the length of the alkoxy spacer chain and the nature of the amino acid residue.

The highest selectivity was exhibited by compound Na_2_**4** (SI = 155), containing a spacer based on ring-opened 12-crown-4 and a tryptophan residue. This compound combines low cytotoxicity (CC_50_ = 155 µg/mL) with high antiviral activity (IC_50_ = 1.0 µg/mL), making it the most promising among the series studied.

Compound Na_2_**5** (SI = 82) also demonstrated high performance; however, its antiviral activity was somewhat inferior to that of Na_2_**4** (IC_50_ = 2.5 µg/mL), with moderately higher cytotoxicity.

Compounds Na_2_**6**–Na_2_**9**, containing longer spacer chains (based on 15-crown-5 and 18-crown-6), exhibited lower selectivity indices (SI = 23–37). This was primarily due to a decrease in antiviral activity (IC_50_ = 7.5–10.0 µg/mL) rather than an increase in cytotoxicity, which remained at a comparable level (CC_50_ = 230–375 µg/mL). This suggests that excessive elongation of the alkoxy spacer chain may reduce the ability of the compounds to interact with the relevant viral target(s).

Thus, it was established that the length of the alkoxy spacer chain and the type of amino acid residue significantly affect the antiviral activity and selectivity of the compounds. The optimal combination was achieved for the derivative with the shortest spacer chain (12-crown-4) and the tryptophan residue (Na_2_**4**), which exhibited the highest selectivity index. The results obtained indicate that the developed bioinorganic systems based on the *closo*-decaborate anion with crown ether-derived spacer groups and amino acid ester pendant moieties represent a promising class of compounds for further development as inhibitors of influenza A virus replication.

Note that neuraminidase inhibitors (Tamiflu and Relenza) are reference drugs for influenza treatment [50]. Oseltamivir targets the conserved neuraminidase active site and is active against all tested subtypes, including A(H1N1)pdm09. In MDCK cell assays, the active metabolite oseltamivir carboxylate is used. However, in co-addition experiments, neuraminidase inhibitors do not block viral replication—this requires alternative methods such as plaque titration or the MUNANA-based neuraminidase activity assay [51]. Reported IC_50_ values for oseltamivir carboxylate range widely (0.0023 → 10 μg/mL), with a median of ~0.3 μg/mL (1 nM); its CC_50_ is ~100 μg/mL, vs. 155 μg/mL for Na_2_**4**.

## 3. Materials and Methods

### 3.1. Materials

Tetrabutylammonium decahydro-*closo*-decaborate (*n*-Bu_4_N)_2_[B_10_H_10_] was prepared according to [52]. Tetrabutylammonium undecahydrodecaborate (*n*-Bu_4_N)[B_10_H_11_] was synthesized following a known procedure [53]. Substituted derivatives (*n*-Bu_4_N)[2-B_10_H_9_O_4_(CH_2_)_8_], (*n*-Bu_4_N)[2-B_10_H_9_O_5_(CH_2_)_10_], and (*n*-Bu_4_N)[2-B_10_H_9_O_6_(CH_2_)_12_] were synthesized by the reaction of the [B_10_H_11_]^−^ anion with the corresponding crown ethers (12-crown-4, 15-crown-5, and 18-crown-6, respectively) under microwave-assisted conditions (2.45 GHz, 700 W) without solvent, using H^+^ (as part of [B_10_H_11_]^−^) as the electrophilic inducer [54]. The reaction proceeds via the EINS mechanism and affords the target products in high yields (81–92%) with a significant reduction in reaction time compared to conventional methods.

15-Crown-5 (98%, Aldrich, Milwaukee, WI, USA), 12-crown-4 (98%, Aldrich), 18-crown-6 (98%, Aldrich), methanol (99.9%, Aldrich), malonic ester (98%, Aldrich), acetonitrile (99%, Aldrich), potassium carbonate (analytical grade, Khimmed, Moscow, Russia), hydrochloric acid (36%, analytical grade, Khimmed), tetraphenylphosphonium chloride (99%, Aldrich), and sodium tetraphenylborate (99.5%, Aldrich) were used without additional purification. L-Amino acid methyl ester hydrochlorides (histidine, tryptophan, methionine, and pyrrolidone), N-methylmorpholine (NMM), and isobutyl chloroformate (IBCF) (99%, Aldrich) were used as received.

### 3.2. Methods

IR spectra of the compounds were recorded on an INFRAlyum FT-02 FT-IR spectrometer (St. Petersburg, Russia) in the range 400–4000 cm^−1^. Samples were prepared as KBr pellets.

^1^H, ^11^B, and ^13^C NMR spectra of solutions in DMSO-*d*_6_ were recorded on a Bruker DPX-300 spectrometer (Rheinstetten, Germany) at 300.3, 96.32, and 75.49 MHz, respectively, with deuterium internal lock.

Electrospray ionization mass spectra (ESI-MS) were recorded using an Agilent 1200 four-channel pump (G1311A) (Waldbronn, Germany) and a TSQ Quantum Access MAX triple quadrupole mass spectrometer (San Jose, CA, USA) equipped with an HESI-II API source and a 6-port injector with a 0.002 cm^3^ external loop. Samples were introduced into the ion source by manual loop injection into the solvent flow. HPLC-grade acetonitrile (ACN) and Milli-Q water were used as solvents. The pump flow rate was set to 0.4 mL/min in isocratic mode ACN/H_2_O (50:50). Electrospray ionization in the negative ion mode (ESI^−^) was performed under the following conditions: spray voltage −2.5 kV, ion transfer tube temperature 350 °C, vaporizer temperature 300 °C, sheath gas (N_2_) pressure 35 arb. units, auxiliary gas flow 10 arb. units. Full-scan mass spectra were recorded in the m/z range 100–1300. Data acquisition and processing were performed using Xcalibur software (version 4.5).

Boron elemental analysis was carried out on an ELAN DRC-e inductively coupled plasma mass spectrometer (PerkinElmer, Mylan, Italy). Carbon, hydrogen, and nitrogen contents were determined on a Eurovector EuroEA 3000 CHNS elemental analyzer (Pavia, Italy).

### 3.3. Syntheses

Tetraphenylphosphonium 2-[2-(2-(2-(2-methylcarboxy)ethoxy)ethoxy)ethoxy]ethoxy]undecahydro-*closo*-decaborate, (Ph_4_P)_2_[B_10_H_9_O(CH_2_CH_2_O)_3_(CH_2_)_3_COOH], (Ph_4_P)_2_**1**.

(*n*-Bu_4_N)[2-B_10_H_9_O_4_(CH_2_)_8_] (1.61 g, 3.0 mmol), malonic ester (1.33 mL, 9.0 mmol), and potassium carbonate (1.54 g, 11.2 mmol) were added to 50 mL of acetonitrile, and the resulting suspension was refluxed with stirring for 16 h. After cooling, the mixture was filtered to remove excess unreacted K_2_CO_3_. The pale yellow filtrate was evaporated almost to dryness to give a small amount of yellow viscous liquid. A solution of 70 mL of 11% hydrochloric acid and 15 mL of ethanol was added. The resulting solution was heated at reflux for 24 h. After cooling, the clear solution was evaporated to dryness, and the obtained white powder was dissolved in water (20 mL). A solution of Ph_4_PCl (2.25 g, 6.0 mmol) in water (25 mL) was added. The precipitated white solid was filtered off and dried under deep vacuum for 2 h at 60 °C. Yield: 2.63 g (85%).

^1^H NMR (DMSO-*d*_6_, δ, ppm, cation signals omitted): −1.00–1.00 (m, 9H), 1.69 (m, 2H), 3.15–3.53 (m, 16H). ^11^B {^1^H} NMR (DMSO-*d*_6_, δ, ppm): −31.0 (s, 1B, B (4)); −26.9 (s, 2B, B (7,8)); −21.2 (s, 4B, B (3,5) + B (6,9)); −3.2 (s, 1B, B (10)); −0.2 (s, 2B, B (1) + B (2)). ^13^C NMR (DMSO-*d*_6_, δ, ppm, cation signals omitted): 174.2, 72.8, 72.1, 70,4, 25.1. IR (KBr), cm^−1^: 3395, 3065, 2867, 2449, 1714, 1589, 1425, 1097, 705, 517. Found (%): B, 10.28; C, 67.22; H, 6.54. For C_58_H_68_B_10_O_6_P_2_ anal. calcd. (%): B, 10.49; C, 67.57; H, 6.60. ESI MS. Found, a.m.u.: 353.42 {[B_10_H_9_O(CH_2_CH_2_O)_3_(CH_2_)_3_COOH]^2−^ + H^+^}^−^. (C_10_H_29_B_10_O_6_). Calcd.: M = 353.44.

Tetraphenylphosphonium 2-[2-(2-(2-(2-(2- methylcarboxy)ethoxy)ethoxy)ethoxy)ethoxy]ethoxy]undecahydro-*closo*-decaborate, (Ph_4_P)_2_[B_10_H_9_O(CH_2_CH_2_O)_4_(CH_2_)_3_COOH], (Ph_4_P)_2_**2**.

(*n*-Bu_4_N)[2-B_10_H_9_O_5_(CH_2_)_10_] (1.74 g, 3.0 mmol), malonic ester (1.33 mL, 9.0 mmol), and potassium carbonate (1.54 g, 11.2 mmol) were added to 50 mL of acetonitrile. The suspension was stirred at room temperature for 12 h and then refluxed with stirring for 8 h. After cooling, the mixture was filtered. The filtrate was evaporated and treated with 70 mL of 11% HCl and 15 mL of ethanol, then refluxed for 24 h. The solution was evaporated to dryness, dissolved in water (20 mL), and Ph_4_PCl (2.25 g, 6.0 mmol) in water (25 mL) was added. The precipitate was filtered off and dried in air. Yield: 2.71 g (83%).

^1^H NMR (DMSO-*d*_6_, δ, ppm, cation signals omitted): −1.00–1.00 (m, 9H), 1.68 (m, 2H), 3.09–3.53 (m, 20H). ^11^B {^1^H} NMR (DMSO-*d*_6_, δ, ppm): −32.1 (s, 1B, B(4)); −27.6 (s, 2B, B (7,8)); −21.7 (s, 4B, B (3,5) + B (6,9)); −3.8 (s, 1B, B (10)); −0.8 (s, 2B, B (1) + B (2)). ^13^C NMR (DMSO-*d*_6_, δ, ppm, cation signals omitted): 174.7, 72.8, 72.5, 70.3, 25.1. IR (KBr), cm^−1^: 3365, 3058, 2867, 2447, 1718, 1589, 1426, 1097, 705, 515. Found (%): B, 9.82; C, 66.79; H, 6.63. For C_60_H_72_B_10_O_7_P_2_ anal. calcd. (%): B, 10.06; C, 67.04; H, 6.70. ESI MS. Found, a.m.u.: 397.46 { [B_10_H_9_O(CH_2_CH_2_O)_4_(CH_2_)_3_COOH]^2−^ + H^+^}^−^. (C_12_H_33_B_10_O_7_). Calcd.: M = 397.49.

Tetraphenylphosphonium 2-[2-(2-(2-(2-(2-(2- methylcarboxy)ethoxy)ethoxy)ethoxy)ethoxy)ethoxy]ethoxy]undecahydro-*closo*-decaborate, (Ph_4_P)_2_[B_10_H_9_O(CH_2_CH_2_O)_5_(CH_2_)_3_COOH], (Ph_4_P)_2_**3**.

(*n*-Bu_4_N)[2-B_10_H_9_O_6_(CH_2_)_12_] (1.87 g, 3.0 mmol), malonic ester (1.33 mL, 9.0 mmol), and potassium carbonate (1.54 g, 11.2 mmol) were added to 50 mL of acetonitrile. The suspension was stirred at room temperature for 12 h and then refluxed with stirring for 2 h. After cooling, the mixture was filtered. The filtrate was evaporated and treated with 70 mL of 11% HCl and 15 mL of ethanol, then refluxed for 24 h. The solution was evaporated to dryness, dissolved in water (20 mL), and Ph_4_PCl (2.25 g, 6.0 mmol) in water (25 mL) was added. The precipitate was filtered and dried. Yield: 2.68 g (80%).

^1^H NMR (DMSO-*d*_6_, δ, ppm, cation signals omitted): −1.00–1.00 (m, 9H), 1.68 (m, 2H), 3.10–3.48 (m, 24H). ^11^B {^1^H} NMR (DMSO-*d*_6_, δ, ppm): −32.6 (s, 1B, B (4)); −28.0 (s, 2B, B (7,8)); −22.2 (s, 4B, B (3,5) + B (6,9)); −3.9 (s, 1B, B (10)); −1.3 (s, 2B, B (1) + B (2)). ^13^C NMR (DMSO-*d*_6_, δ, ppm, cation signals omitted): 175.4, 72.8, 72.4, 70.2, 25.2. IR (KBr), cm^−1^: 3335, 3058, 2867, 2447, 1718, 1589, 1426, 1097, 705, 515. Found (%): B, 9.51; C, 66.24; H, 6.74. For C_62_H_76_B_10_O_8_P_2_ anal. calcd. (%): B, 9.67; C, 66.55; H, 6.80. ESI MS. Found, a.m.u.: 441.51 {[B_10_H_9_O(CH_2_CH_2_O)_4_(CH_2_)_3_COOH]^2−^ + H^+^}^−^. (C_14_H_37_B_10_O_8_). Calcd.: M = 441.54.

General procedure for the synthesis of (Ph_4_P)_2_[B_10_H_9_O(CH_2_CH_2_O)*_n_*(CH_2_)_3_C(O)XOMe] (X = Trp, His). The corresponding carboxylic acid (Ph_4_P)_2_[B_10_H_9_O(CH_2_CH_2_O)*_n_*(CH_2_)_3_COOH] (0.30 mmol) was dissolved in 20.0 mL of CH_2_Cl_2_, NMM (0.032 mL, 0.30 mmol) was added, and the mixture was cooled to −25 °C. IBCF (0.038 mL, 0.30 mmol) was added with stirring. After 15 min, a suspension of the corresponding L-amino acid methyl ester hydrochloride and NMM in equimolar ratio in 15.0 mL of CH_2_Cl_2_ was added. The mixture was stirred for 1 h at −20 °C, then for 2 h at 0 °C, and finally for 18 h at 25 °C. The reaction mixture was washed successively with 0.25 M H_2_SO_4_ (1 × 4.0 mL), 0.25 M KHCO_3_ (2 × 10.0 mL), and H_2_O (1 × 5.0 mL). The organic layer was dried over anhydrous Na_2_SO_4_ and evaporated under vacuum (45 °C; 25 mm Hg) to give an oily residue. The oil was triturated with 10.0 mL of diethyl ether on a Vortex shaker and re-evaporated to afford a white crystalline product.

Tetraphenylphosphonium methyl 2-[2-(2-(2-(2-carbonyl)amino]-3-(1H-indol-2-yl)propanoate)ethoxy)ethoxy)ethoxy)ethoxy]undecahydro-*closo*-decaborate, (Ph_4_P)_2_[B_10_H_9_O(CH_2_CH_2_O)_3_(CH_2_)_3_C(O)TrpOMe], (Ph_4_P)_2_**4**. Prepared from (Ph_4_P)_2_**1** (0.3 g, 0.244 mmol) and HCl·H-Trp-OMe (0.074 g, 0.244 mmol). Yield: 0.34 g (94.97%).

^1^H NMR (DMSO-*d*_6_, δ, ppm): −1.00–1.00 (m, 9H), 1.59 (2H, m, -CH_2_-); 2.11 (2H, m, CH_2_, Trp), 3.13–3.59 (m, 16H), 3.26 (3H, s, O-CH_3_); 4.48 (m, 1H, CH, Trp); 5.75 (s, 1H, Trp), 6.96–7.46 (ar, Trp); 10.91 (s, 1H, Trp). ^11^B {^1^H} NMR (DMSO-*d*_6_, δ, ppm): −31.4 (s, 1B, B (4)); −27.1 (s, 2B, B (7,8)); −21.1 (s, 4B, B (3,5) + B (6,9)); −3.5 (s, 1B, B (10)); 0.0 (s, 2B, B (1) + B (2)). ^13^C NMR (DMSO-*d*_6_, δ, ppm, cation signals omitted): 173.3, 172.5, 127.5, 124.3, 121.4, 112.0, 110.1, 72.8, 71.6, 70.4, 60.7, 52.3, 32.1, 27.3, 25.7. IR (KBr), cm^−1^: 3193, 2924, 2452, 1728, 1429, 705, 515. Found (%): B, 8.61; C, 68.02; H, 6.51; N, 2.23. For C_70_H_80_B_10_N_2_O_7_P_2_ anal. calcd. (%): B, 8.78; C, 68.27; H, 6.55; N, 2.27. ESI MS. Found, m/z: 554.05 {H^+^ + [B_10_H_9_O(CH_2_CH_2_O)_3_(CH_2_)_3_C(O)TrpOMe]^2−^}. (C_22_H_42_B_10_N_2_O_7_). Calcd.: M = 554.10.

Sodium salt Na_2_[B_10_H_9_O(CH_2_CH_2_O)_3_(CH_2_)_3_C(O)TrpOMe], Na_2_**4**. (Ph_4_P)_2_**4** (0.37 g, 0.30 mmol) was dissolved in 2 mL of methanol. A solution of Na[BPh_4_] (0.21 g, 0.60 mmol) in 3 mL of methanol was added. The precipitated white solid was filtered off, and the filtrate was evaporated to give a white hygroscopic powder, which was dried under deep vacuum. Yield: 0.098 g (55%).

Tetraphenylphosphonium methyl 2-[2-(2-(2-(2-carbonyl)amino]-3-(1H-imidazol-4-yl)propanoate)ethoxy)ethoxy)ethoxy)ethoxy]undecahydro-*closo*-decaborate, (Ph_4_P)_2_[B_10_H_9_O(CH_2_CH_2_O)_3_(CH_2_)_3_C(O)HisOMe], (Ph_4_P)_2_**5**. Prepared analogously from (Ph_4_P)_2_**1** (0.3 g, 0.254 mmol) and 2HCl·H-His-OMe (0.061 g, 0.254 mmol). Yield: 0.288 g (83.72%).

^1^H NMR (DMSO-*d*_6_, δ, ppm): −1.00–1.00 (m, 9H), 1.61 (2H, m, -CH_2_-); 2.12 (2H, m, CH_2_, His), 3.13–3.64 (m, 16H), 3.40 (3H, s, O-CH_3_); 4.15 (m, 1H, CH, His); 6.55 (s, 1H, His), 7.31, 8.18, 8.76, 8.81 (ar, His). ^11^B {^1^H} NMR (DMSO-*d*_6_, δ, ppm): −31.2 (s, 1B, B (4)); −27.0 (s, 2B, B (7,8)); −21.1 (s, 4B, B (3,5) + B (6,9)); −3.2 (s, 1B, B (10)); −0.2 (s, 2B, B (1) + B (2)). ^13^C NMR (DMSO-*d*_6_, δ, ppm, cation signals omitted): 172.7, 171.8, 129.2, 118.7, 72.8, 72.5, 70.2, 60.8, 52.6, 31.9, 27.7, 25.1. IR (KBr), cm^−1^: 3213, 2921, 2452, 1732, 1423, 1097, 705, 516. Found (%): B, 8.98 C, 65.72; H, 6.51; N, 3.52. For C_65_H_77_B_10_N_3_O_7_P_2_ anal. calcd. (%): B, 9.14 C, 66.03; H, 6.56; N, 3.55. ESI MS. Found, m/z: 505.03 {H^+^ + [B_10_H_9_O(CH_2_CH_2_O)_3_(CH_2_)_3_C(O)HisOMe]^2−^}. (C_17_H_39_B_10_N_3_O_7_). Calcd.: M = 505.10.

Sodium salt Na_2_[B_10_H_9_O(CH_2_CH_2_O)_3_(CH_2_)_3_C(O)HisOMe], Na_2_**5**. Prepared analogously from (Ph_4_P)_2_[B_10_H_9_O(CH_2_CH_2_O)_3_(CH_2_)_3_C(O)HisOMe] (0.35 g, 0.30 mmol) and Na[BPh_4_] (0.21 g, 0.60 mmol). Yield: 0.091 g (55%).

Tetraphenylphosphonium methyl 2-[2-(2-(2-(2-(2-carbonyl)amino]-3-(1H-indol-2-yl)propanoate)ethoxy)ethoxy)ethoxy)ethoxy)ethoxy]undecahydro-*closo*-decaborate, (Ph_4_P)_2_[B_10_H_9_O(CH_2_CH_2_O)_4_(CH_2_)_3_C(O)TrpOMe], (Ph_4_P)_2_**6**. Prepared analogously from (Ph_4_P)_2_**4** (0.3 g, 0.235 mmol) and HCl·H-Trp-OMe (0.060 g, 0.235 mmol). Yield: 0.337 g (94.66%).

^1^H NMR (DMSO-*d*_6_, δ, ppm): −1.00–1.00 (m, 9H), 1.41 (2H, m, -CH_2_-); 2.07 (2H, m, CH_2_, Trp), 3.15–3.56 (m, 20H), 3.34 (3H, s, O-CH_3_); 4.24 (m, 1H, CH, Trp); 6.53 (s, 1H, Trp), 7.04–7.96 (ar, Trp); 10.87 (s, 1H, Trp). ^11^B {^1^H} NMR (DMSO-*d*_6_, δ, ppm): −31.3 (s, 1B, B (4)); −27.2 (s, 2B, B (7,8)); −21.3 (s, 4B, B (3,5) + B (6,9)); −3.1 (s, 1B, B (10)); −0.1 (s, 2B, B (1) + B (2)). ^13^C NMR (DMSO-*d*_6_, δ, ppm, cation signals omitted): 173.0, 172.6, 127.5, 124.3, 121.4, 111.9, 110.0, 72.8, 70.4, 60.2, 52.3, 32.3, 27.3, 25.7. IR (KBr), cm^−1^: 3202, 2868, 2450, 1727, 1430, 705, 515. Found (%): B, 8.33 C, 67.52; H, 6.60; N, 2.17; N, 2.23. For C_72_H_84_B_10_N_2_O_8_P_2_ anal. calcd. (%): B, 8.47 C, 67.80; H, 6.64; N, 2.20. ESI MS. Found, m/z: 598.03 {H^+^ + [B_10_H_9_O(CH_2_CH_2_O)_4_(CH_2_)_3_C(O)TrpOMe]^2−^}. (C_24_H_46_B_10_N_2_O_8_). Calcd.: M = 598.10.

Sodium salt Na_2_[B_10_H_9_O(CH_2_CH_2_O)_4_(CH_2_)_3_C(O)TrpOMe], Na_2_**6.** Prepared analogously from (Ph_4_P)_2_[B_12_H_11_O(CH_2_)_2_O(CH_2_)_3_C(O)TrpOMe] (0.38 g, 0.30 mmol) and Na[BPh_4_] (0.21 g, 0.60 mmol). Yield: 0.110 g (57%).

Tetraphenylphosphonium methyl 2-[2-(2-(2-(2-(2-carbonyl)amino]-3-(1H-imidazol-4-yl)propanoate)ethoxy)ethoxy)ethoxy)ethoxy)ethoxy]undecahydro-*closo*-decaborate, (Ph_4_P)_2_[B_10_H_9_O(CH_2_CH_2_O)_4_(CH_2_)_3_C(O)HisOMe], (Ph_4_P)_2_**7**. Prepared analogously from (Ph_4_P)_2_**2** (0.3 g, 0.245 mmol) and 2HCl·H-His-OMe (0.059 g, 0.245 mmol). Yield: 0.215 g (62.86%).

^1^H NMR (DMSO-*d*_6_, δ, ppm): −1.00–1.00 (m, 9H), 1.21 (2H, m, -CH_2_-); 2.07 (2H, m, CH_2_, His), 3.10–3.62 (m, 20H), 3.52 (3H, s, O-CH_3_); 4.12 (m, 1H, CH, His); 6.53 (s, 1H, His), 7.28, 8.74 (ar, His). ^11^B {^1^H} NMR (DMSO-*d*_6_, δ, ppm): −31.7 (s, 1B, B (4)); −27.2 (s, 2B, B (7,8)); −21.2 (s, 4B, B (3,5) + B (6,9)); −3.3 (s, 1B, B (10)); −0.4 (s, 2B, B (1) + B (2)). ^13^C NMR (DMSO-*d*_6_, δ, ppm, cation signals omitted): 172.2, 171.2, 129.1, 118.7, 72.6, 72.5, 70.4, 60.7, 51.4, 31.2, 27.7, 25.8. IR (KBr), cm^−1^: 3219, 2866, 2448, 1732, 1425, 1097, 705, 515. Found (%): B, 8.64 C, 65.33; H, 6.60; N, 3.39. For C_67_H_81_B_10_N_3_O_8_P_2_ anal. calcd. (%): B, 8.81 C, 65.62; H, 6.66; N, 3.43. ESI MS. Found, m/z: 549.04 {H^+^ + [B_10_H_9_O(CH_2_CH_2_O)_4_(CH_2_)_3_C(O)HisOMe]^2−^}. (C_19_H_43_B_10_N_3_O_8_). Calcd.: M = 549.10.

Sodium salt Na_2_[B_10_H_9_O(CH_2_CH_2_O)_4_(CH_2_)_3_C(O)HisOMe], Na_2_**7**. Prepared analogously from (Ph_4_P)_2_[B_10_H_9_O(CH_2_CH_2_O)_4_(CH_2_)_3_C(O)HisOMe] (0.37 g, 0.30 mmol) and Na[BPh_4_] (0.21 g, 0.60 mmol). Yield: 0.091 g (51%).

Tetraphenylphosphonium methyl 2-[2-(2-(2-(2-(2-(2-carbonyl)amino]-3-(1H-indol-2-yl)propanoate)ethoxy)ethoxy)ethoxy)ethoxy)ethoxy]undecahydro-*closo*-decaborate, (Ph_4_P)_2_[B_10_H_9_O(CH_2_CH_2_O)_5_(CH_2_)_3_C(O)TrpOMe], (Ph_4_P)_2_**8**.

Prepared analogously from (Ph_4_P)_2_**3** (0.3 g, 0.227 mmol) and HCl·H-Trp-OMe (0.058 g, 0.227 mmol). Yield: 0.330 g (93.22%).

^1^H NMR (DMSO-*d*_6_, δ, ppm): −1.00–1.00 (m, 9H), 1.21 (2H, m, -CH_2_-); 2.07 (2H, m, CH_2_, Trp), 3.15–3.58 (m, 24H), 3.38 (3H, s, O-CH_3_); 4.23 (m, 1H, CH, Trp); 6.53 (s, 1H, Trp), 6.96–7.98 (ar, Trp); 10.88 (s, 1H, Trp). ^11^B {^1^H} NMR (DMSO-*d*_6_, δ, ppm): −31.8 (s, 1B, B (4)); −27.0 (s, 2B, B (7,8)); −21.3 (s, 4B, B (3,5) + B (6,9)); −3.4 (s, 1B, B (10)); −0.5 (s, 2B, B (1) + B (2)). ^13^C NMR (DMSO-*d*_6_, δ, ppm, cation signals omitted): 173.3, 171.9, 127.2, 124.3, 121.4, 111.9, 110.1, 72.8, 72.5, 70.2, 60.7, 52.3, 32.3, 28.1, 25.7. IR (KBr), cm^−1^: 3205, 2866, 2449, 1730, 1430, 705, 516. Found (%): B, 8.05 C, 67.13; H, 6.65; N, 2.06. For C_74_H_88_B_10_N_2_O_9_P_2_ anal. calcd. (%): B, 8.19 C, 67.36; H, 6.72; N, 2.12. ESI MS. Found, m/z: 642.02 {H^+^ + [B_10_H_9_O(CH_2_CH_2_O)_5_(CH_2_)_3_C(O)TrpOMe]^2−^}. (C_26_H_50_B_10_N_2_O_9_). Calcd.: M = 642.10.

Sodium salt Na_2_[B_10_H_9_O(CH_2_CH_2_O)_5_(CH_2_)_3_C(O)TrpOMe], Na_2_**8**. Prepared analogously from (Ph_4_P)_2_[B_12_H_11_O(CH_2_)_2_O(CH_2_)_3_C(O)TrpOMe] (0.40 g, 0.30 mmol) and Na[BPh_4_] (0.21 g, 0.60 mmol). Yield: 0.102 g (53%).

Tetraphenylphosphonium methyl 2-[2-(2-(2-(2-(2-(2-carbonyl)amino]-3-(1H-imidazol-4-yl)propanoate)ethoxy)ethoxy)ethoxy)ethoxy)ethoxy]undecahydro-*closo*-decaborate, (Ph_4_P)_2_[B_10_H_9_O(CH_2_CH_2_O)_5_(CH_2_)_3_C(O)HisOMe], (Ph_4_P)_2_**9**.

Prepared analogously from (Ph_4_P)_2_**3** (0.3 g, 0.245 mmol) and 2HCl·H-His-OMe (0.059 g, 0.245 mmol). Yield: 0.290 g (85.29%).

^1^H NMR (DMSO-*d*_6_, δ, ppm): −1.00–1.00 (m, 9H), 1.21 (2H, m, -CH_2_-); 2.07 (2H, m, CH_2_, His), 3.21–3.62 (m, 24H), 3.48 (3H, s, O-CH_3_); 4.10 (m, 1H, CH, His); 6.54 (s, 1H, His), 7.30, 8.88 (ar, His). ^11^B {^1^H} NMR (DMSO-*d*_6_, δ, ppm): −31.5 (s, 1B, B (4)); −27.1 (s, 2B, B (7,8)); −21.5 (s, 4B, B (3,5) + B (6,9)); −3.4 (s, 1B, B (10)); −0.3 (s, 2B, B (1) + B (2)). ^13^C NMR (DMSO-*d*_6_, δ, ppm, cation signals omitted): 173.5, 171.8, 129.4, 118.7, 72.8, 72.3, 70.2, 60.7, 51.5, 31.2, 27.7, 25.3. IR (KBr), cm^−1^: 3212, 2919, 2451, 1732, 1424, 1097, 705, 515. Found (%): B, 8.35 C, 64.89; H, 6.70; N, 3.26. For C_69_H_85_B_10_N_3_O_9_P_2_ anal. calcd. (%): B, 8.51 C, 65.23; H, 6.74; N, 3.31. ESI MS. Found, m/z: 593.05 {H^+^ + [B_10_H_9_O(CH_2_CH_2_O)_5_(CH_2_)_3_C(O)HisOMe]^2−^}. (C_21_H_47_B_10_N_3_O_9_). Calcd.: M = 593.10.

Sodium salt Na_2_[B_10_H_9_O(CH_2_CH_2_O)_5_(CH_2_)_3_C(O)HisOMe], Na_2_**9**. Prepared analogously from (Ph_4_P)_2_[B_10_H_9_O(CH_2_CH_2_O)_5_(CH_2_)_3_C(O)HisOMe] (0.38 g, 0.30 mmol) and Na[BPh_4_] (0.21 g, 0.60 mmol). Yield: 0.092 g (52%).

### 3.4. Study of Cytotoxic Properties

The cytotoxicity of compounds Na_2_**4**–Na_2_**9** was evaluated on a three-day confluent monolayer of the MDCK cell line (NBL-2: CCL-34, ATCC) grown in Eagle’s MEM medium supplemented with 10% fetal bovine serum, L-glutamine, and antibiotics (150 U/mL penicillin and 150 µg/mL streptomycin). The 50% cytotoxic concentration (CC_50_) was determined according to the standard procedure described previously [31].

### 3.5. Study of Antiviral Properties

The antiviral activity of the compounds was investigated in vitro in 96-well plates with a confluent (100%) MDCK cell monolayer. The tests were performed against the contemporary influenza virus strain A/Moscow/78/2020 (H1N1)pdm09, which contains the drug-resistance mutation (S31N) to adamantane-class drugs such as rimantadine and amantadine. Six compound concentrations were used: 0.5, 1.0, 2.5, 5.0, 7.5, and 10.0 µg/mL. The test compounds were added to the cell culture simultaneously with the influenza A virus at two multiplicities of infection (10^−3^ and 10^−4^). The results of antiviral activity were recorded by cell ELISA by measuring optical density in experimental and control wells, as described previously [31].

## 4. Conclusions

In this study, a series of novel substituted derivatives of the *closo*-decaborate anion Na_2_**4**–Na_2_**9** were synthesized, containing alkoxy spacer chains of varying lengths derived from ring-opened crown ethers (12-crown-4, 15-crown-5, and 18-crown-6) with L-amino acid methyl ester residues (L-tryptophan and L-histidine) as pendant groups. The compounds were evaluated for their cytotoxic and antiviral properties against the influenza A virus strain A/Moscow/78/2020 (H1N1)pdm09.

The results demonstrated that the length of the alkoxy spacer chain significantly affects both antiviral activity and selectivity. The shortest spacer chain (derived from 12-crown-4) proved to be optimal, while elongation of the chain led to a decrease in antiviral potency. Compound Na_2_**4**, containing a 12-crown-4-based spacer and a tryptophan residue, exhibited the highest selectivity index (SI = 155) with low cytotoxicity (CC_50_ = 155 µg/mL) and high antiviral activity (IC_50_ = 1.0 µg/mL). Tryptophan-containing derivatives consistently showed better performance than their histidine analogues. A clear structure–activity relationship was established: increasing the spacer chain length from 8 to 12 atoms resulted in a decline in the selectivity index from 155 to 23–37.

Overall, the *closo*-decaborate platform with crown ether-derived spacers and amino acid ester pendant groups represents a promising scaffold for the development of low-toxicity, highly selective inhibitors of influenza A virus replication. Compound Na_2_**4** merits further investigation as a lead candidate among the samples prepared.

## Figures and Tables

**Table 1:** Cytotoxic concentrations (CC_50_), half-maximal inhibitory concentrations (IC_50_), and selectivity indices (SI = CC_50_/IC_50_) of compounds Na_2_**4**–Na_2_**9** against influenza A virus strain A/Moscow/78/2020 (H1N1)pdm09 in MDCK cells.

Compounds	CC_50_, µg/mL (mM)	IC_50_, µg/mL	SI
Na_2_**4**	155 (0.258)	1.0	155
Na_2_**5**	206 (0.386)	2.5	82
Na_2_**6**	375 (0.591)	10.0	37
Na_2_**7**	250 (0.416)	10.0	25
Na_2_**8**	230 (0.328)	10.0	23
Na_2_**9**	240 (0.379)	7.5	32

## Data Availability

No new data were created or analyzed in this study. Data sharing is not applicable to this article.

## Supplementary Materials

The following supporting information can be downloaded at: https://www.mdpi.com/article/10.3390/molecules31183157/s1, Figures S1–S45: spectroscopic data.
