# Supplementary material for: Influence of Spacer Chains Derived from Ring-Opened Crown Ethers on the Anti-Influenza A Virus Activity of closo-Decaborates Bearing Amino Acid Ester Pendant Moieties
Source: Molecules. 2026 Sep 8;31(18):3157. doi: 10.3390/molecules31183157 (PMC13609459; doi:10.3390/molecules31183157)
Supplement: Supplementary file 1 [file molecules-31-03157-s001.zip › molecules-4484203-supplementary.pdf]

SUPPLEMENTARY INFORMATION

for

Influence of Spacer Chains Derived from Ring-Opened Crown Ethers on the Anti-Influenza A Virus Activity of *closo*-Decaborates Bearing Amino Acid Ester Pendant Moieties

Contents

$(Ph_4P)_2[B_{10}H_9O(CH_2CH_2O)_3(CH_2)_3COOH]$, $(Ph_4P)_2$**1**....3

Fig. S1. ^{11}B {^{1}H} NMR spectra in DMSO-d_6....3

Fig. S2. ^{1}H NMR spectrum in DMSO-d_6. 3

Fig. S3. ^{13}C NMR spectrum in DMSO-d_6. 4

Fig. S4. Anionic part of the ESI mass-spectrum. 5

Fig. S5. IR spectrum. 5

$(Ph_4P)_2[B_{10}H_9O(CH_2CH_2O)_4(CH_2)_3COOH]$, $(Ph_4P)_2$**2**....5

Fig. S6. ^{11}B {^{1}H} NMR spectrum in DMSO-d_6. 6

Fig. S7. ^{1}H NMR spectrum in DMSO-d_6. 6

Fig. S8. ^{13}C NMR spectrum in DMSO-d_6. 6

Fig. S9. Anionic part of the ESI mass-spectrum. 7

Fig. S10. IR spectrum. 7

$(Ph_4P)_2[B_{10}H_9O(CH_2CH_2O)_5(CH_2)_3COOH]$, $(Ph_4P)_2$**3**....8

Fig. S11. ^{11}B {^{1}H} NMR spectrum in DMSO-d_6. 8

Fig. S12. ^{1}H NMR spectrum in DMSO-d_6. 8

Fig. S13. ^{13}C NMR spectrum in DMSO-d_6. 9

Fig. S14. Anionic part of the ESI mass-spectrum. 9

Fig. S15. IR spectrum. 10

$(Ph_4P)_2[B_{10}H_9O(CH_2CH_2O)_3(CH_2)_3C(O)TrpOMe]$, $(Ph_4P)_2$**4**....11

Fig. S16. ^{11}B {^{1}H} NMR spectrum in DMSO-d_6. 11

Fig. S17. ^{1}H NMR spectrum in DMSO-d_6. 11

Fig. S18. ^{13}C NMR spectrum in DMSO-d_6. 12

Fig. S19. IR spectrum. 12

Fig. S20. Anionic part of the ESI mass-spectrum. 13

$(Ph_4P)_2[B_{10}H_9O(CH_2CH_2O)_3(CH_2)_3C(O)HisOMe]$, $(Ph_4P)_2$**5**....14

Fig. S21. ^{11}B {^{1}H} NMR spectrum in DMSO-d_6. 14

Fig. S22. ^{1}H NMR spectrum in DMSO-d_6. 14

Fig. S23. ^{13}C NMR spectrum in DMSO-d_6. 15
Fig. S24. IR spectrum. 15
Fig. S25. Anionic part of the ESI mass-spectrum. 16
$(Ph_4P)_2[B_{10}H_9O(CH_2CH_2O)_4(CH_2)_3C(O)TrpOMe]$, $(Ph_4P)_2$**6** 17
Fig. S26. $^{11}B\{^1H\}$ NMR spectrum in DMSO-d_6. 17
Fig. S27. ^1H NMR spectrum in DMSO-d_6. 17
Fig. S28. ^{13}C NMR spectrum in DMSO-d_6. 18
Fig. S29. IR spectrum. 18
Fig. S30. Anionic part of the ESI mass-spectrum. 19
$(Ph_4P)_2[B_{10}H_9O(CH_2CH_2O)_4(CH_2)_3C(O)HisOMe]$, $(Ph_4P)_2$**7** 20
Fig. S31. $^{11}B\{^1H\}$ NMR spectrum in DMSO-d_6. 20
Fig. S32. ^1H NMR spectrum in DMSO-d_6. 21
Fig. S33. ^{13}C NMR spectrum in DMSO-d_6. 21
Fig. S34. IR spectrum. 21
Fig. S35. Anionic part of the ESI mass-spectrum. 22
$(Ph_4P)_2[B_{10}H_9O(CH_2CH_2O)_5(CH_2)_3C(O)TrpOMe]$, $(Ph_4P)_2$**8** 23
Fig. S36. $^{11}B\{^1H\}$ NMR spectrum in DMSO-d_6. 23
Fig. S37. ^1H NMR spectrum in DMSO-d_6. 23
Fig. S38. ^{13}C NMR spectrum in DMSO-d_6. 24
Fig. S39. IR spectrum. 24
Fig. S40. Anionic part of the ESI mass-spectrum. 25
$(Ph_4P)_2[B_{10}H_9O(CH_2CH_2O)_5(CH_2)_3C(O)HisOMe]$, $(Ph_4P)_2$**9** 26
Fig. S41. $^{11}B\{^1H\}$ NMR spectrum in DMSO-d_6. 26
Fig. S42. ^1H NMR spectrum in DMSO-d_6. 26
Fig. S43. ^{13}C NMR spectrum in DMSO-d_6. 27
Fig. S44. IR spectrum. 27
Fig. S45. Anionic part of the ESI mass-spectrum. 28
^{11}B NMR monitoring of the oxonium ring-opening in the derivatives $[B_{10}H_9O_4(CH_2)_8]^-$, $[B_{10}H_9O_5(CH_2)_{10}]^-$ and $[B_{10}H_9O_6(CH_2)_{12}]^-$ with malonic ester in the presence of K_2CO_3 29
1) Stirring at room temperature for 12 h 29
2) Refluxing for 2 h 30
3) Refluxing for 8 h 32
4) Refluxing for 16 h 33
Details of biological experiments 34

$(Ph_4P)_2[B_{10}H_9O(CH_2CH_2O)_3(CH_2)_3COOH]$, $(Ph_4P)_2$**1**

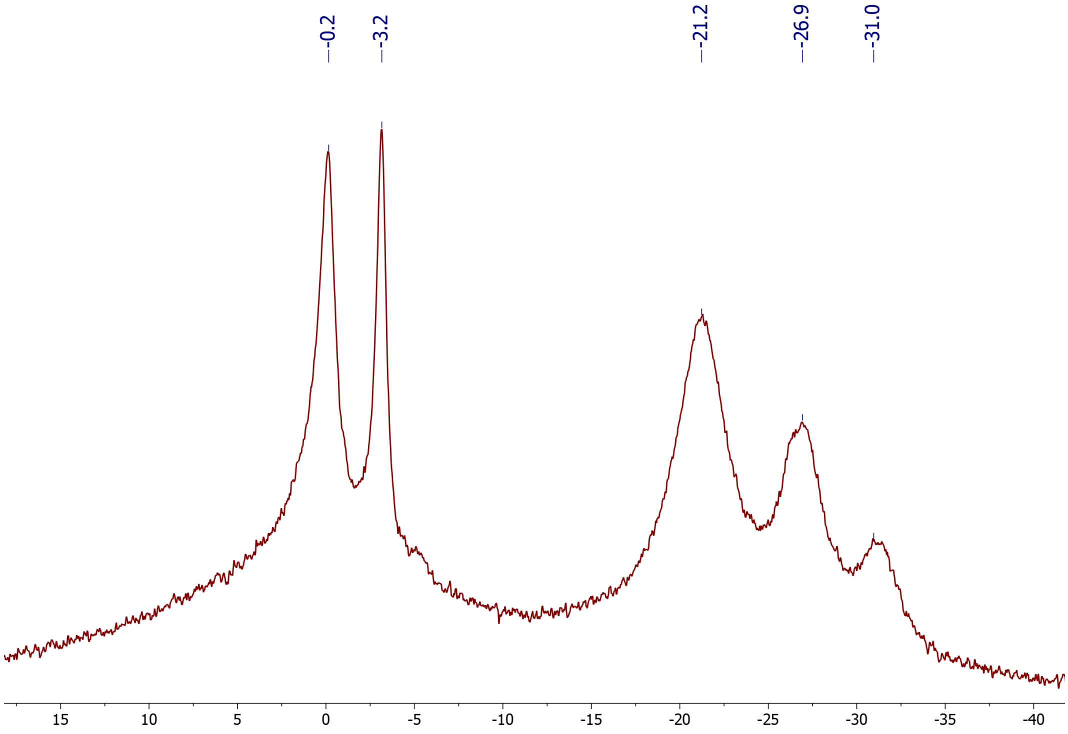

Fig. S1. $^{11}B\ \{^1H\}$ NMR spectra in DMSO-d$_6$.

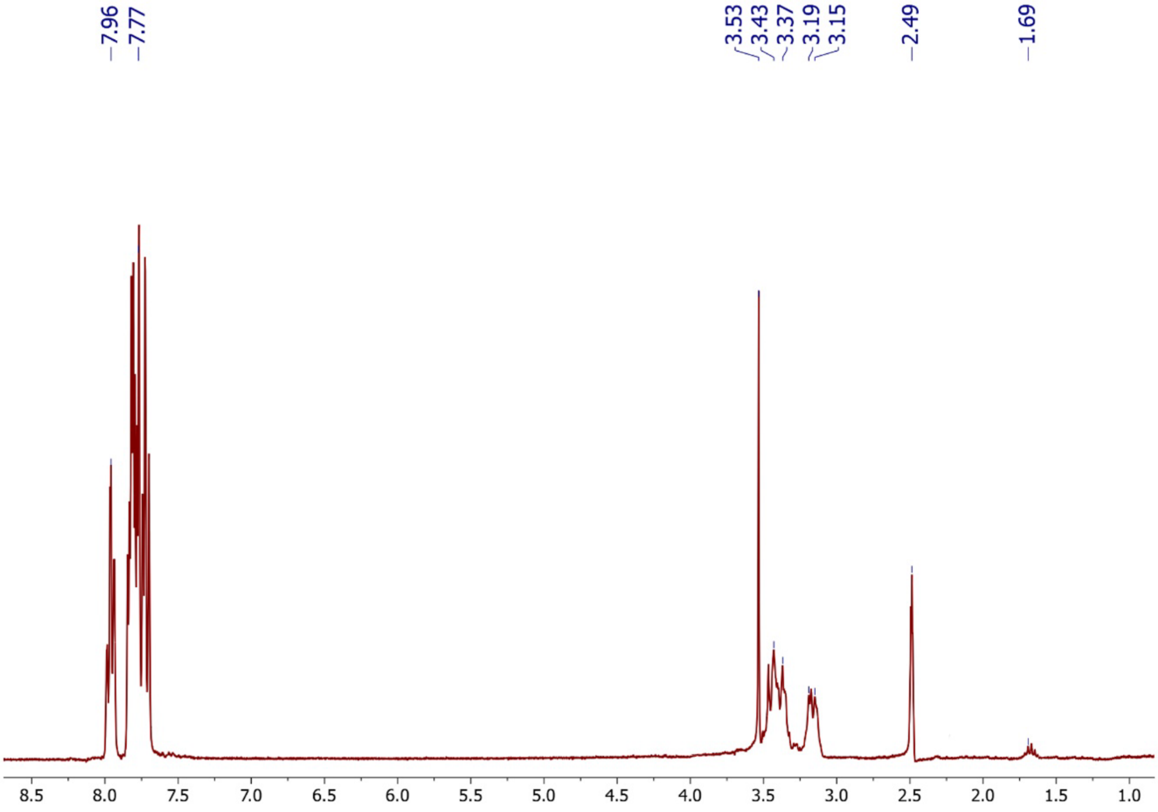

Fig. S2. ^1H NMR spectrum in DMSO-d$_6$.

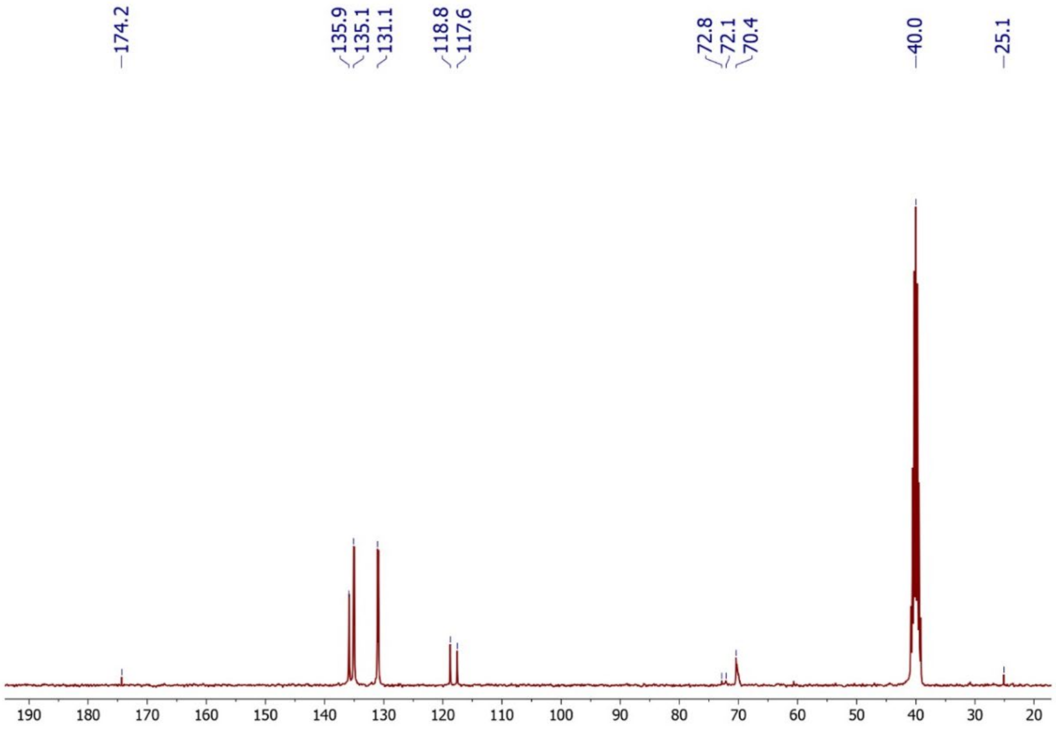

Fig. S3. ^{13}C NMR spectrum in DMSO-d_6.

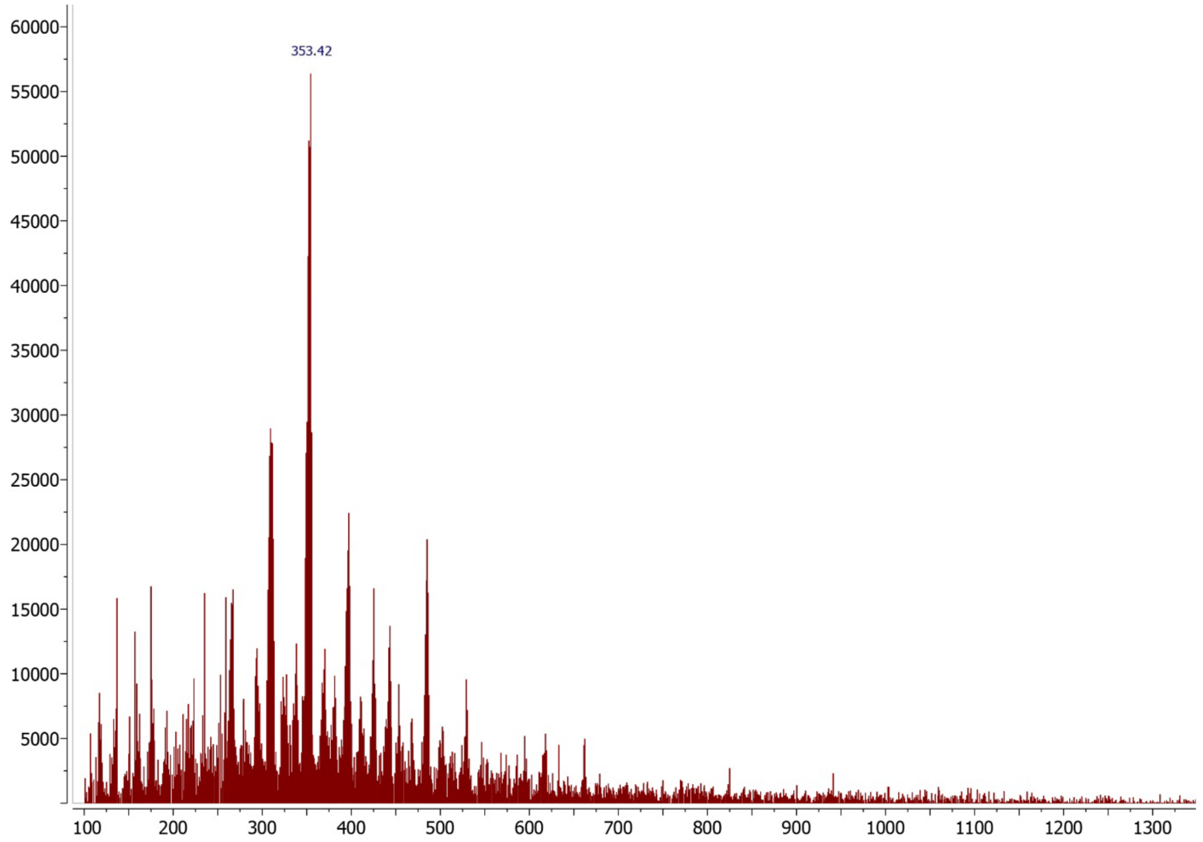

Fig. S4. Anionic part of the ESI mass-spectrum.

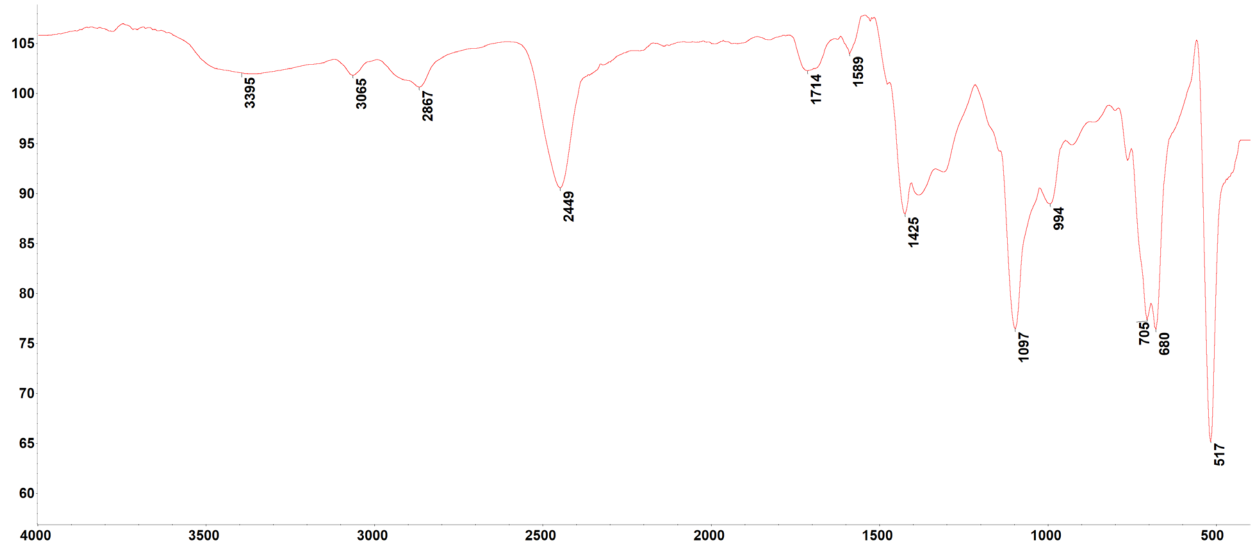

Fig. S5. IR spectrum.

$(Ph_4P)_2[B_{10}H_9O(CH_2CH_2O)_4(CH_2)_3COOH]$, $(Ph_4P)_2$**2**

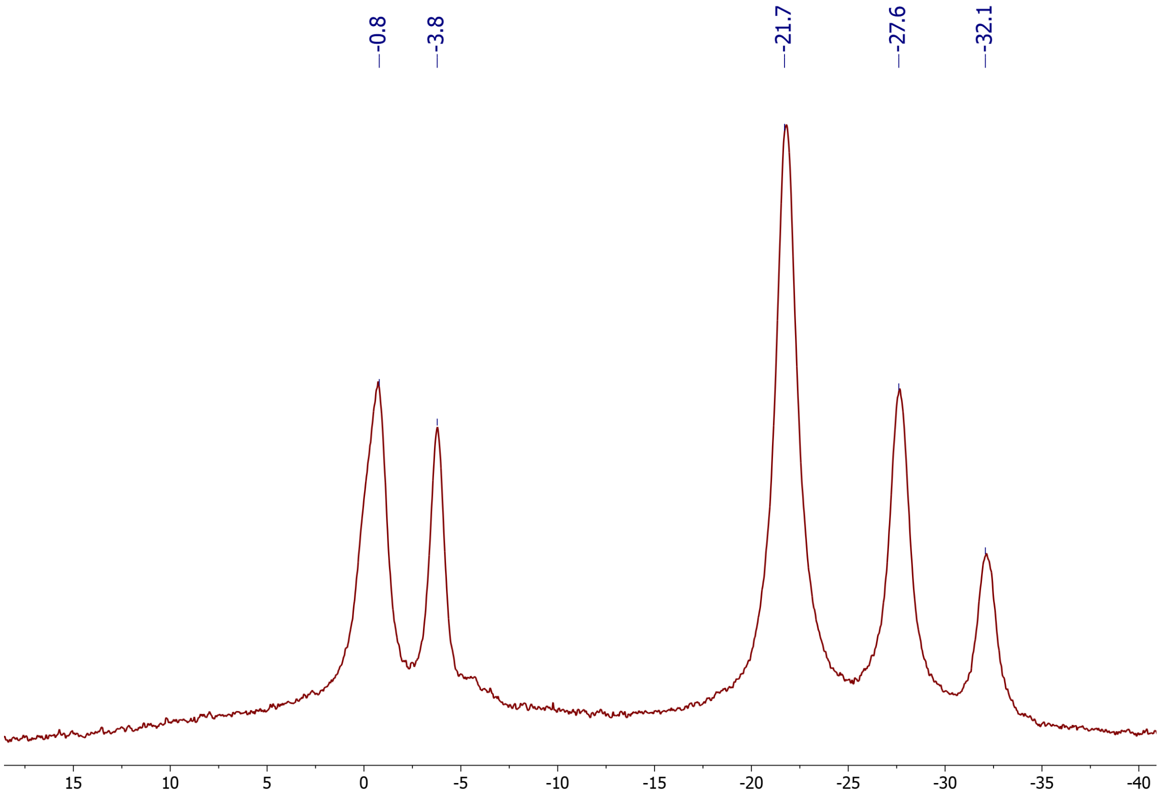

Fig. S6. ^{11}B {^{1}H} NMR spectrum in DMSO-d_6.

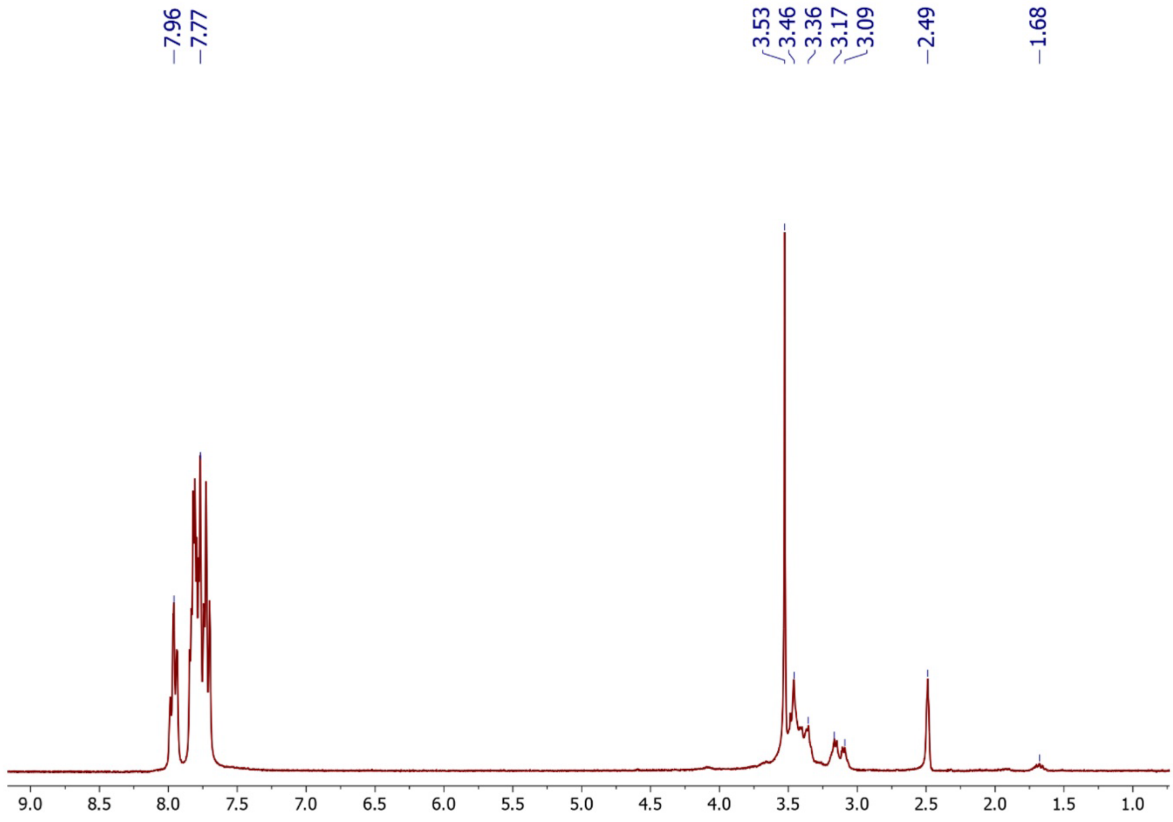

Fig. S7. ^{1}H NMR spectrum in DMSO-d_6.

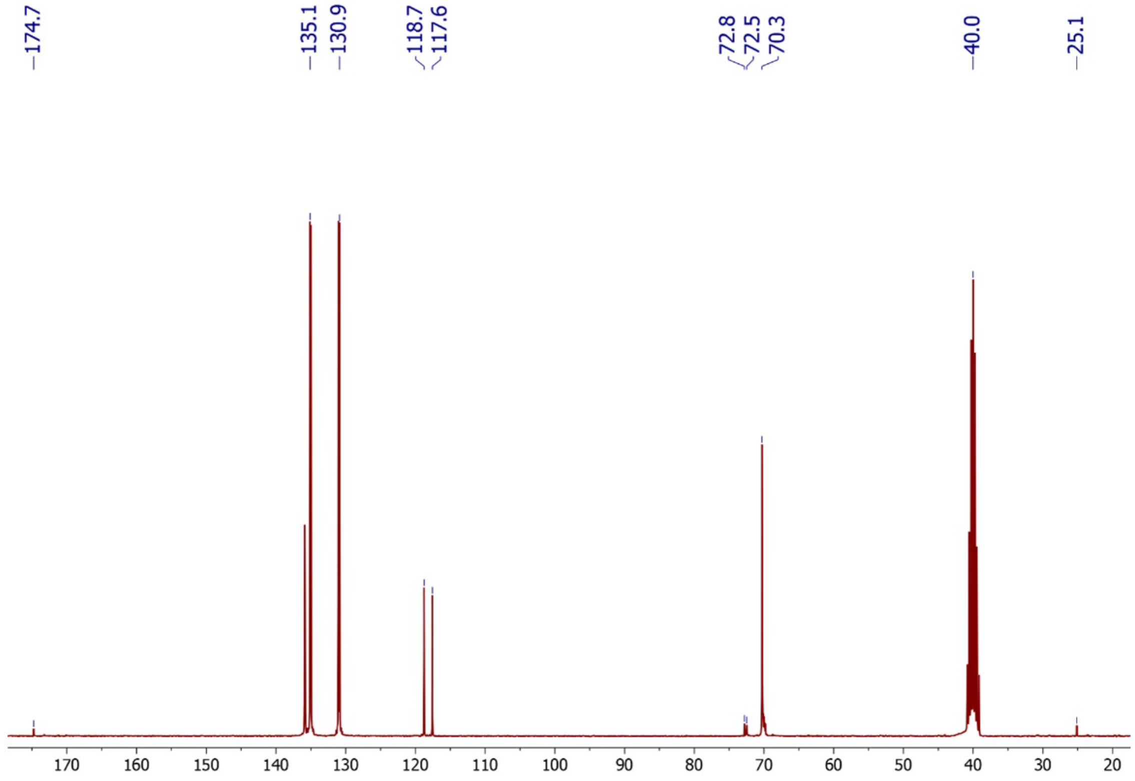

Fig. S8. ^{13}C NMR spectrum in DMSO-d_6.

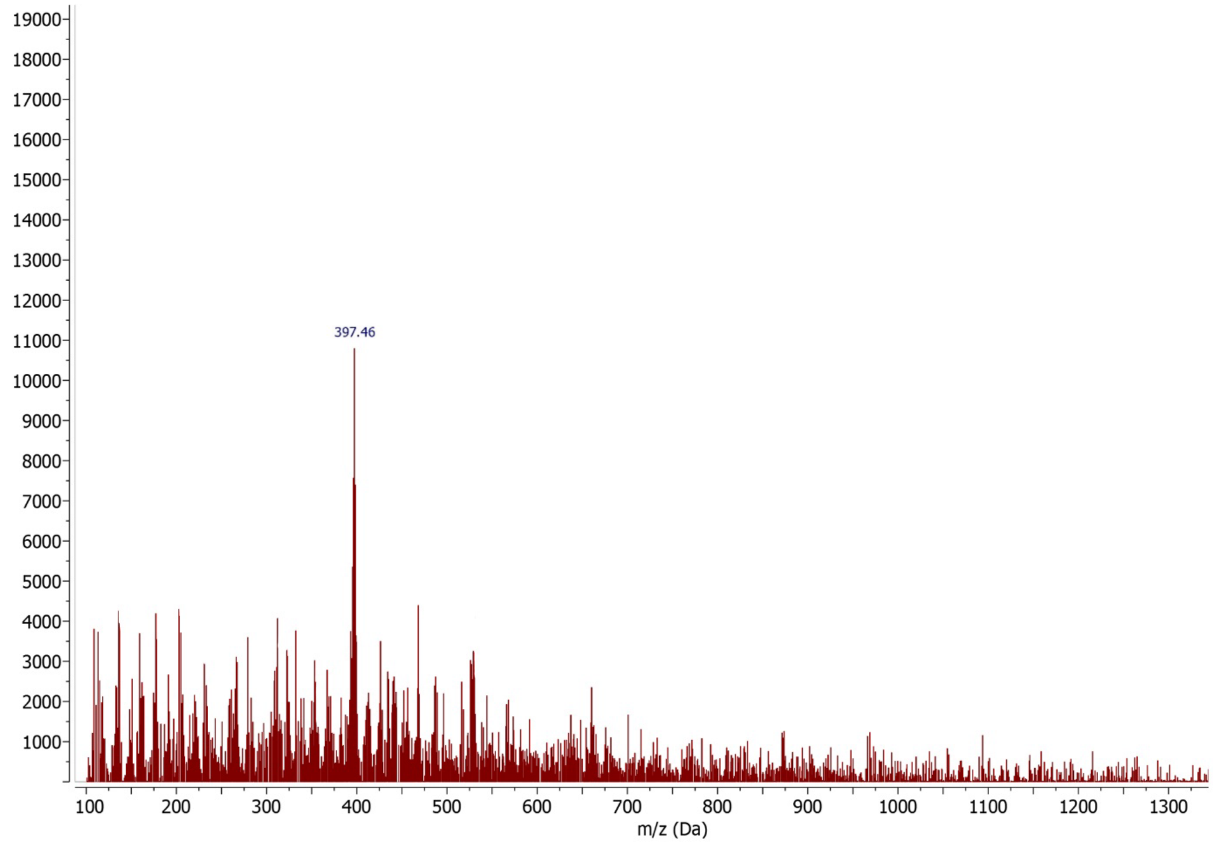

Fig. S9. Anionic part of the ESI mass-spectrum.

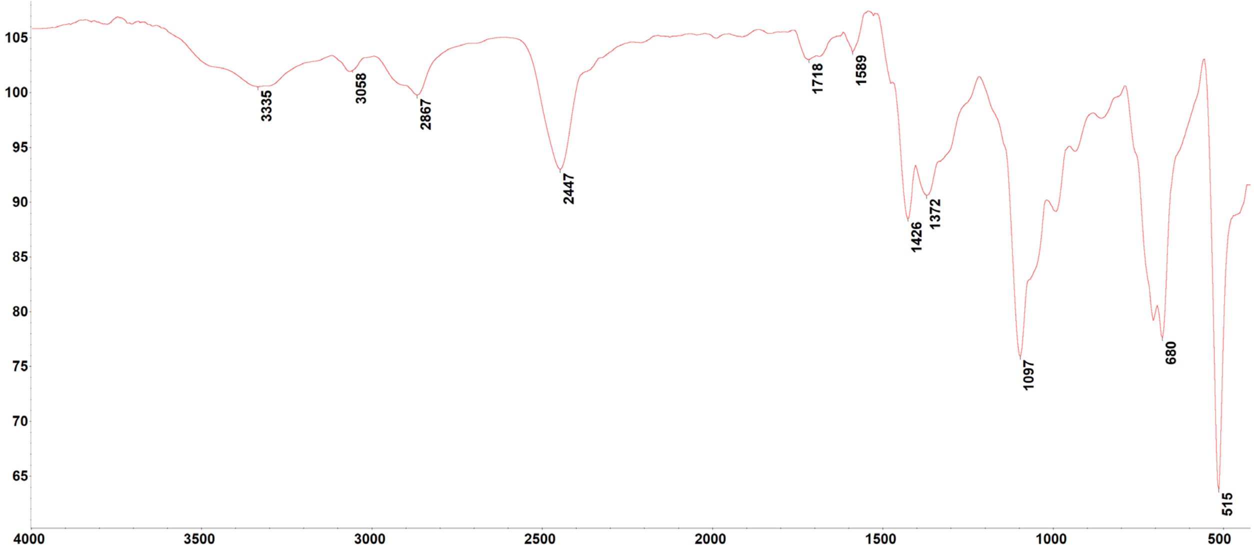

Fig. S10. IR spectrum.

$(Ph_4P)_2[B_{10}H_9O(CH_2CH_2O)_5(CH_2)_3COOH]$, $(Ph_4P)_2$**3**

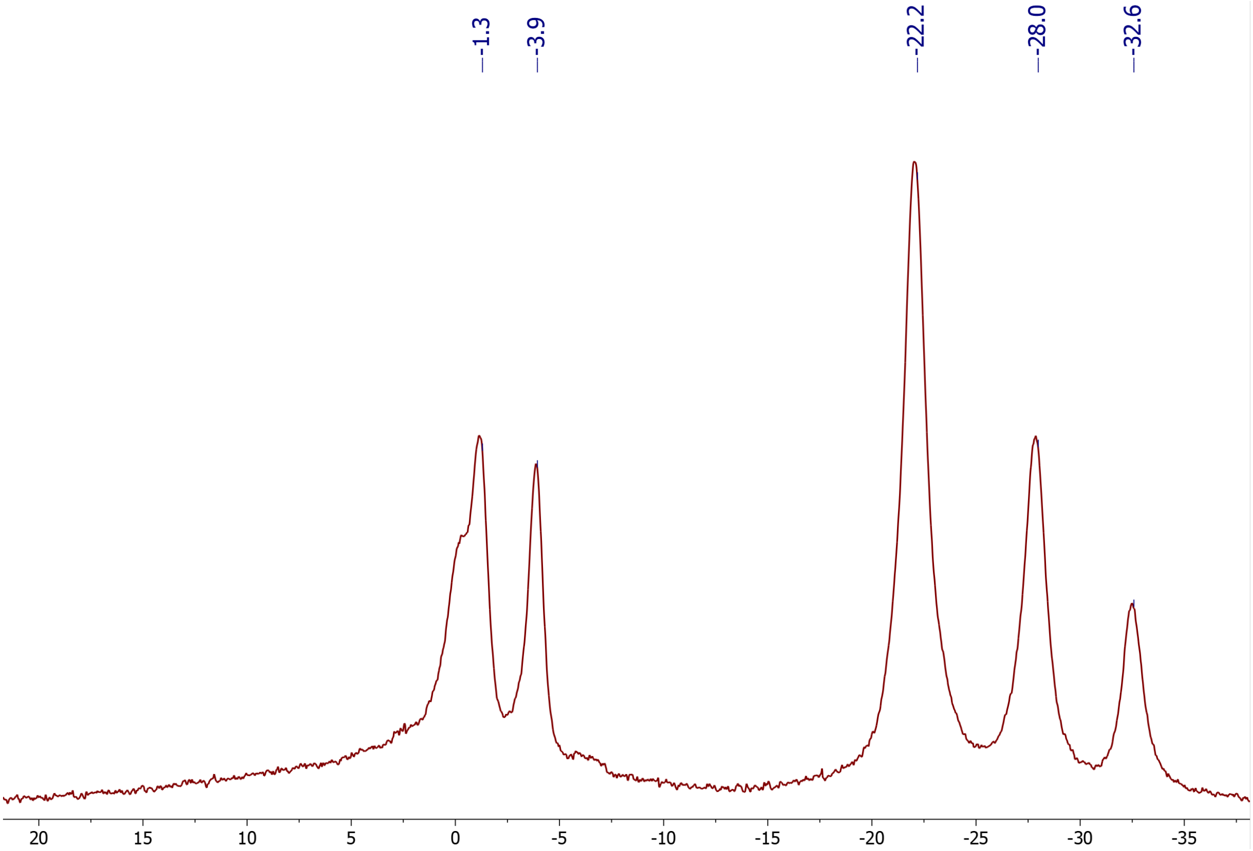

Fig. S11. $^{11}B\ \{^1H\}$ NMR spectrum in DMSO-d$_6$.

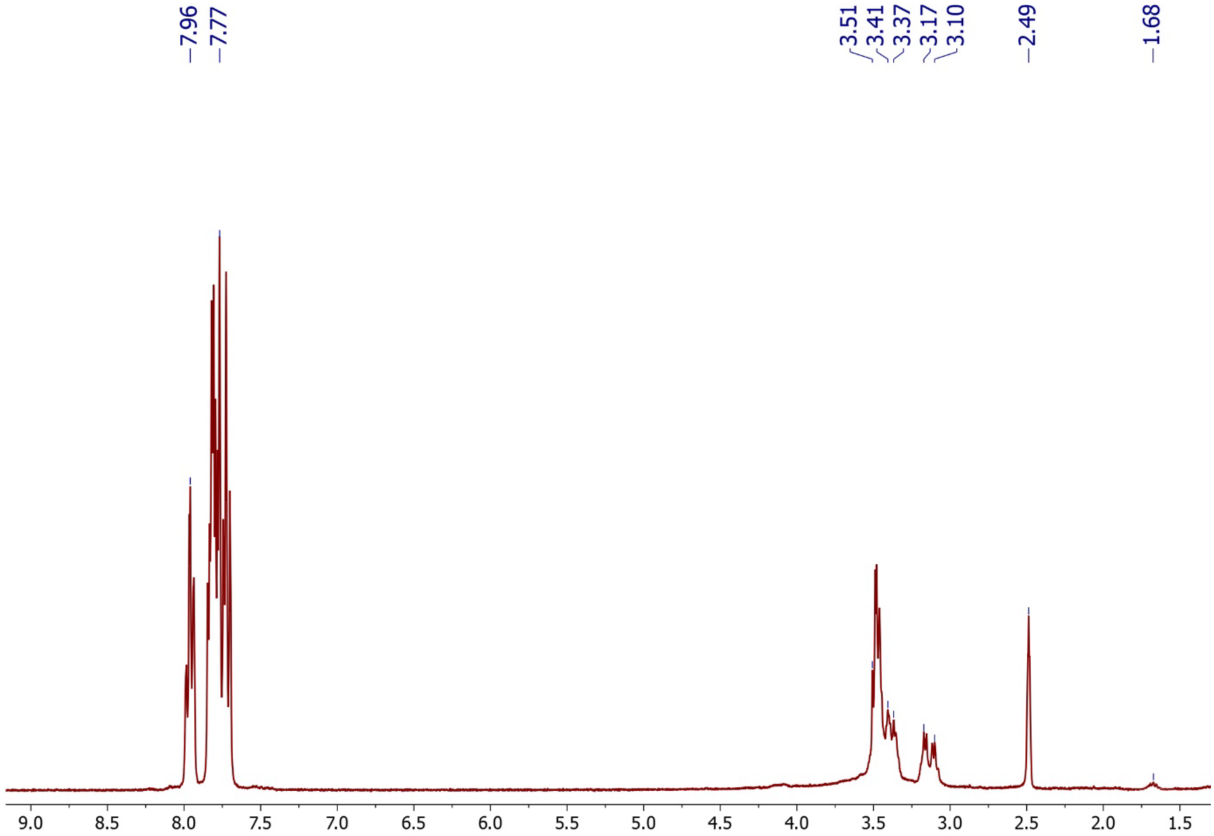

Fig. S12. ^1H NMR spectrum in DMSO-d$_6$.

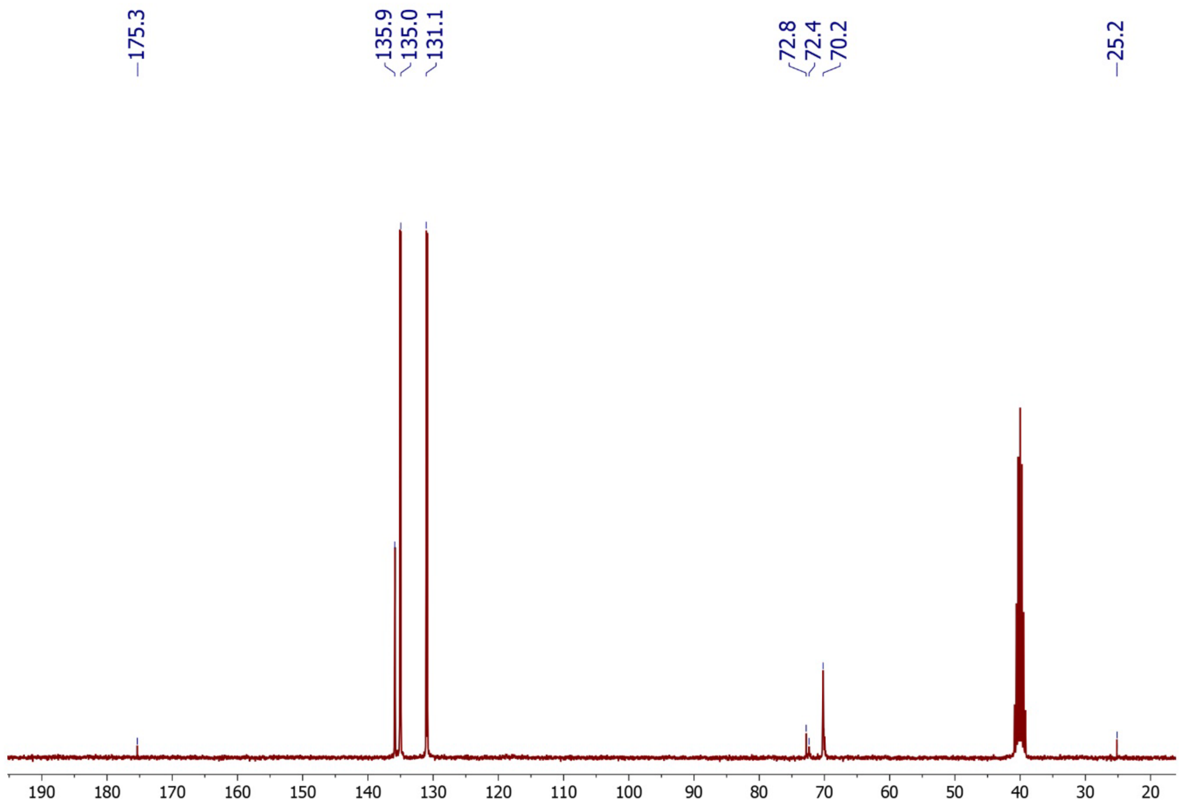

Fig. S13. ^{13}C NMR spectrum in DMSO-d_6.

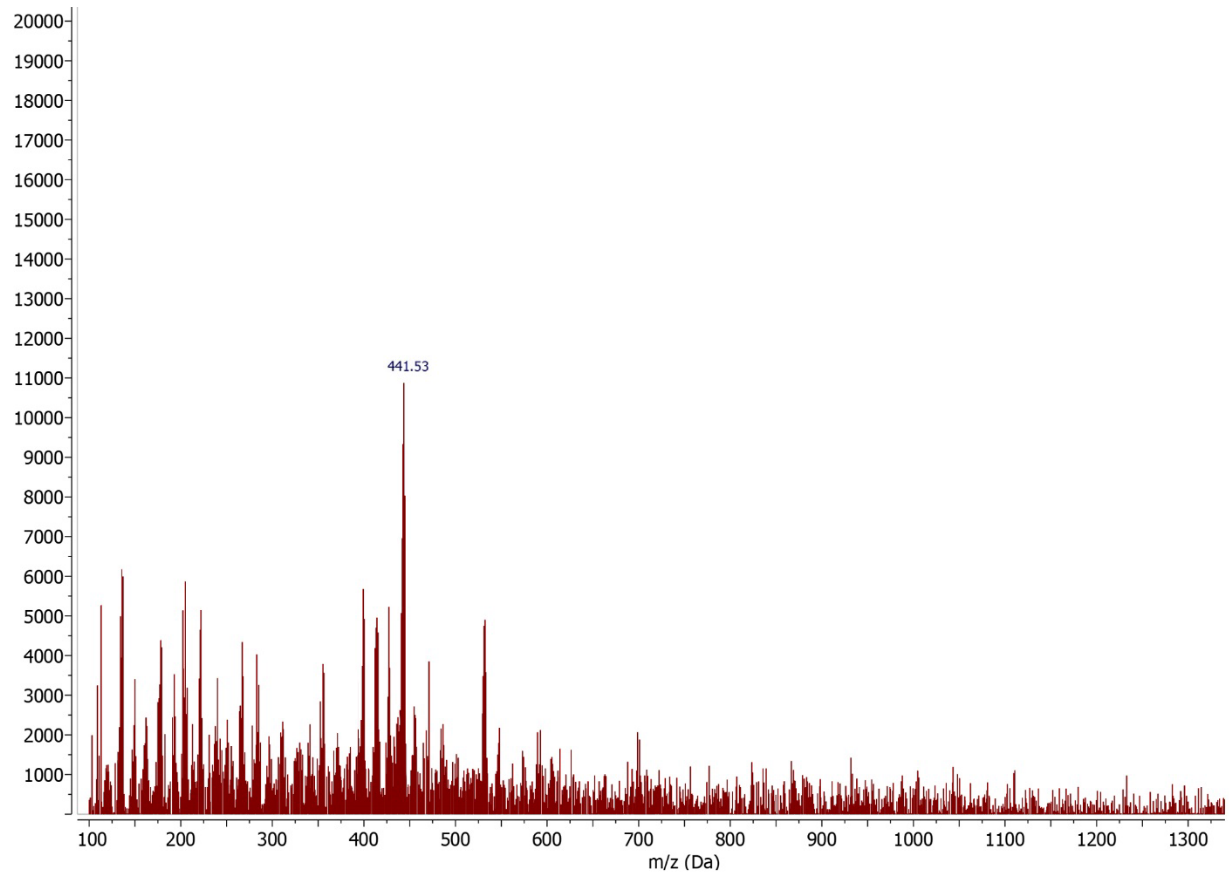

Fig. S14. Anionic part of the ESI mass-spectrum.

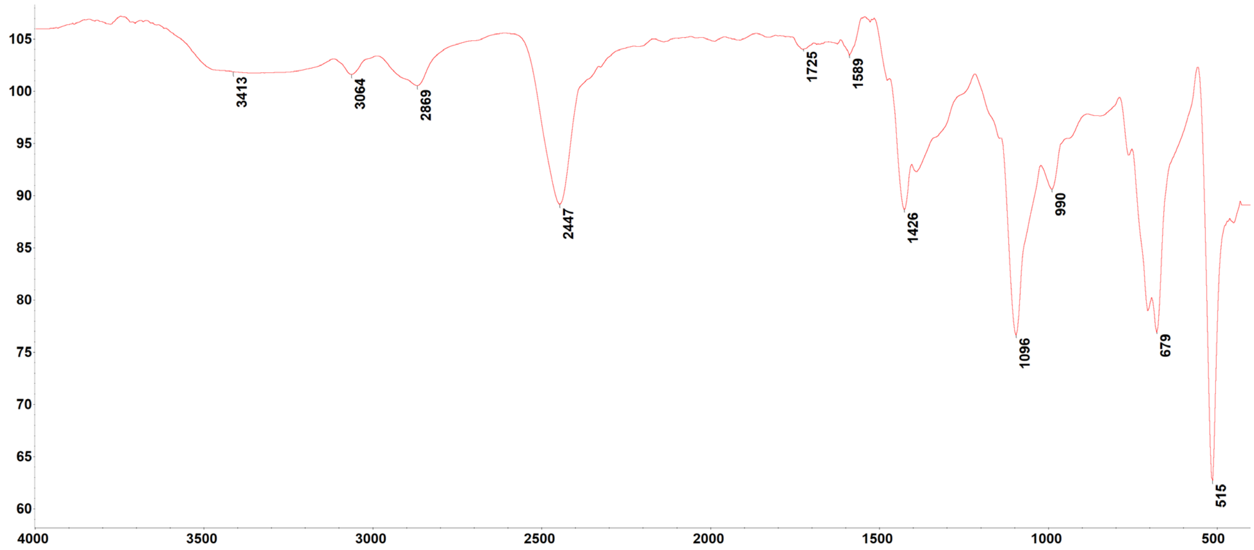

Fig. S15. IR spectrum.

$(Ph_4P)_2[B_{10}H_9O(CH_2CH_2O)_3(CH_2)_3C(O)TrpOMe]$, $(Ph_4P)_2$**4**

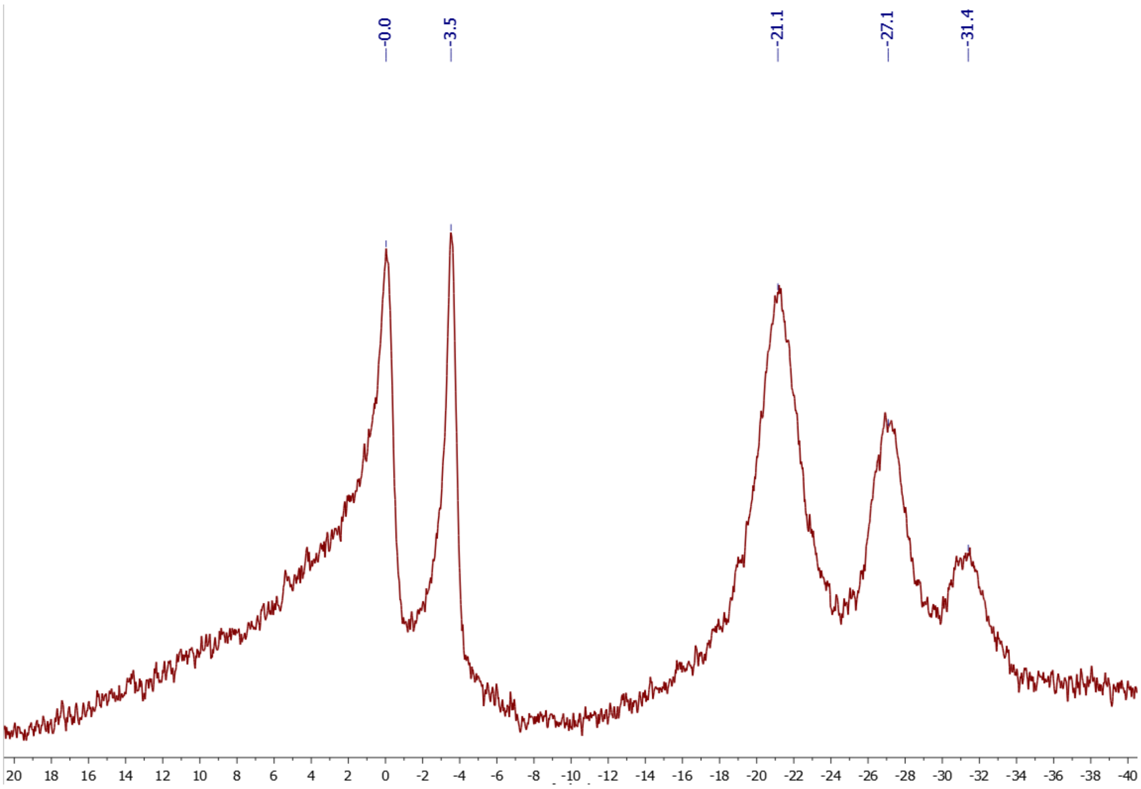

Fig. S16. $^{11}B\{^{1}H\}$ NMR spectrum in DMSO-d_6.

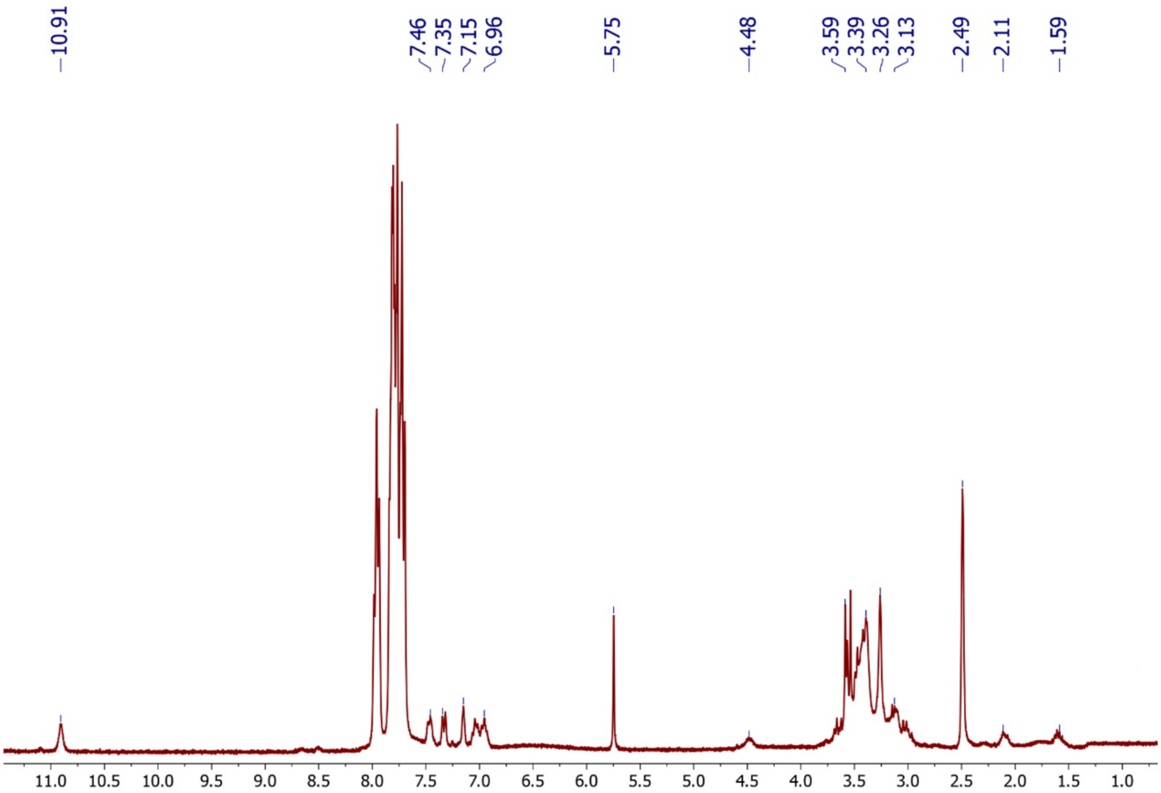

Fig. S17. ^{1}H NMR spectrum in DMSO-d_6.

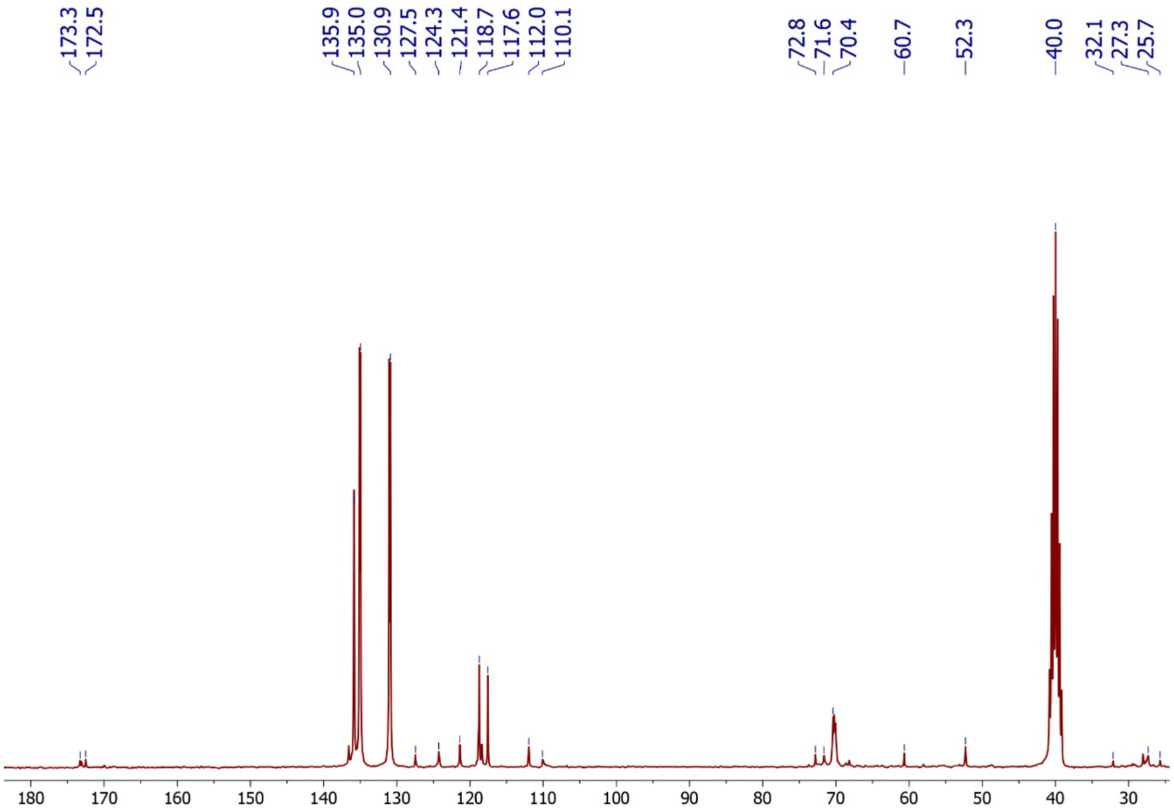

Fig. S18. ^{13}C NMR spectrum in DMSO-d_6.

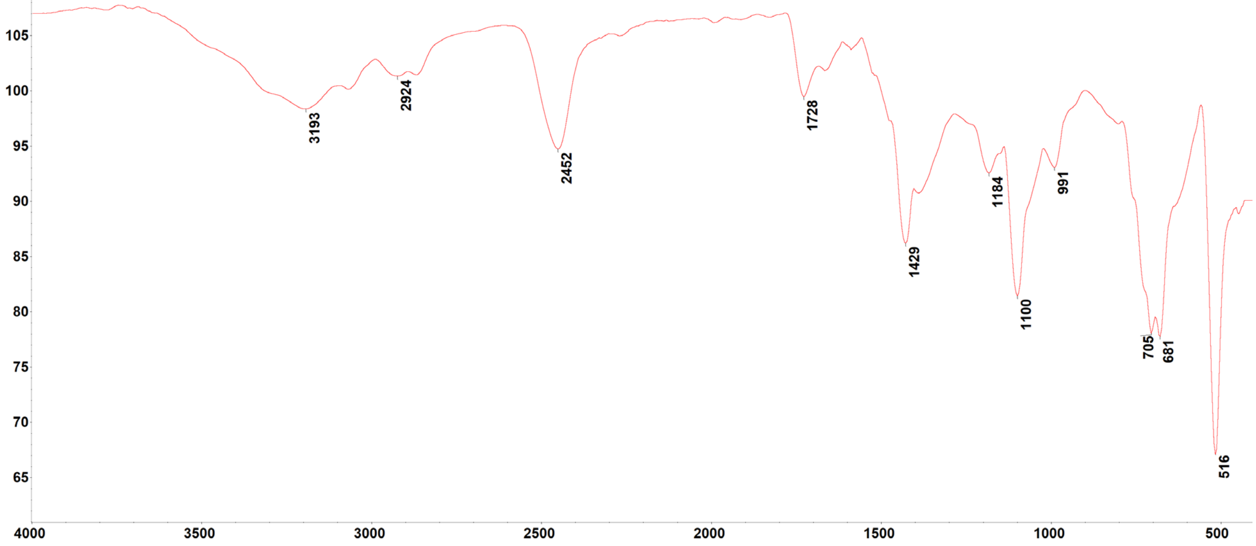

Fig. S19. IR spectrum.

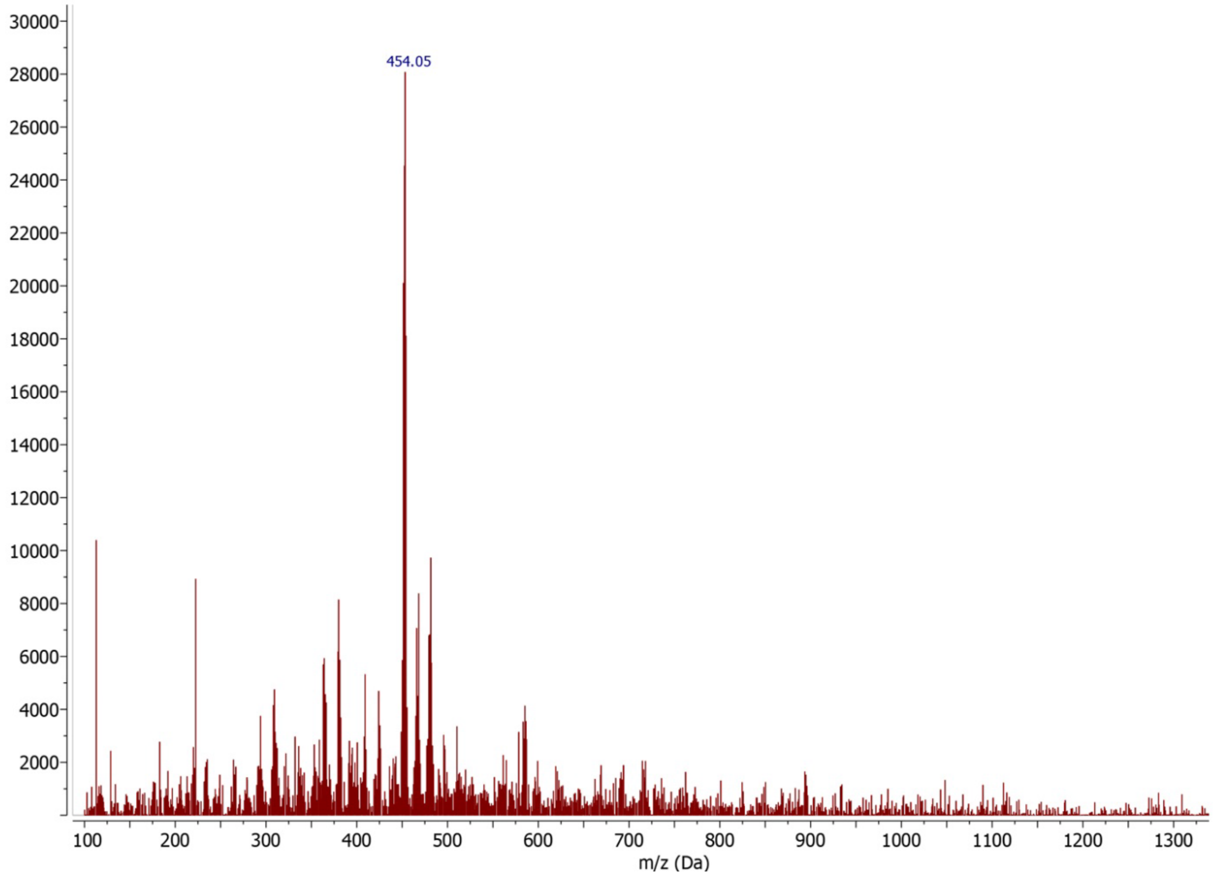

Fig. S20. Anionic part of the ESI mass-spectrum.

$(Ph_4P)_2[B_{10}H_9O(CH_2CH_2O)_3(CH_2)_3C(O)HisOMe]$, $(Ph_4P)_2$**5**

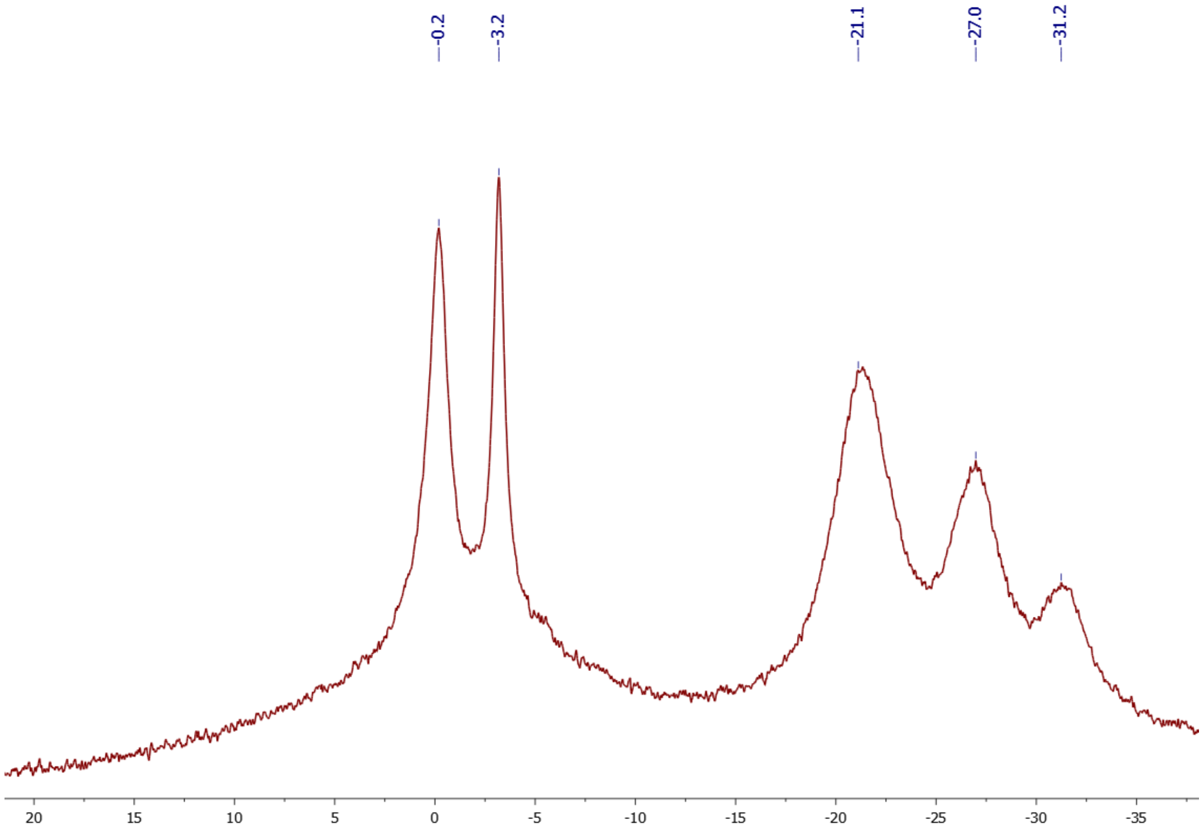

Fig. S21. ^{11}B {^{1}H} NMR spectrum in DMSO-d_6.

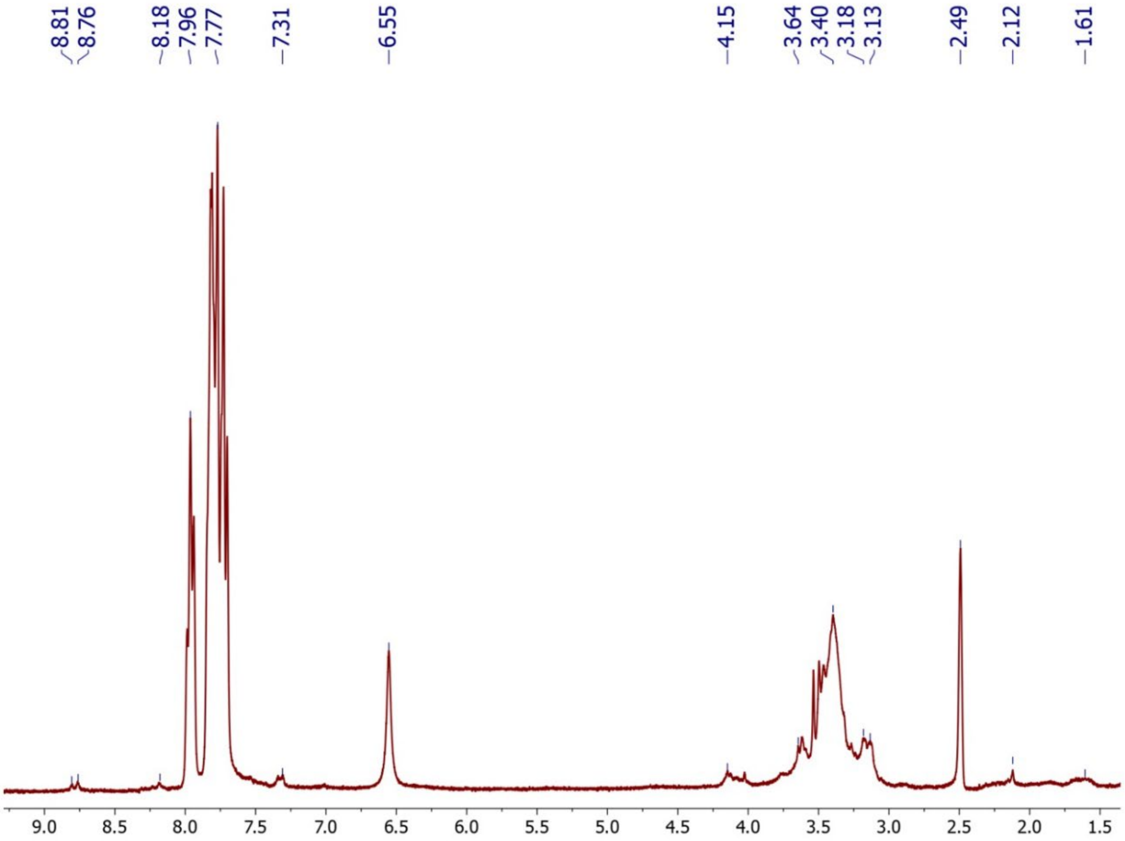

Fig. S22. ^{1}H NMR spectrum in DMSO-d_6.

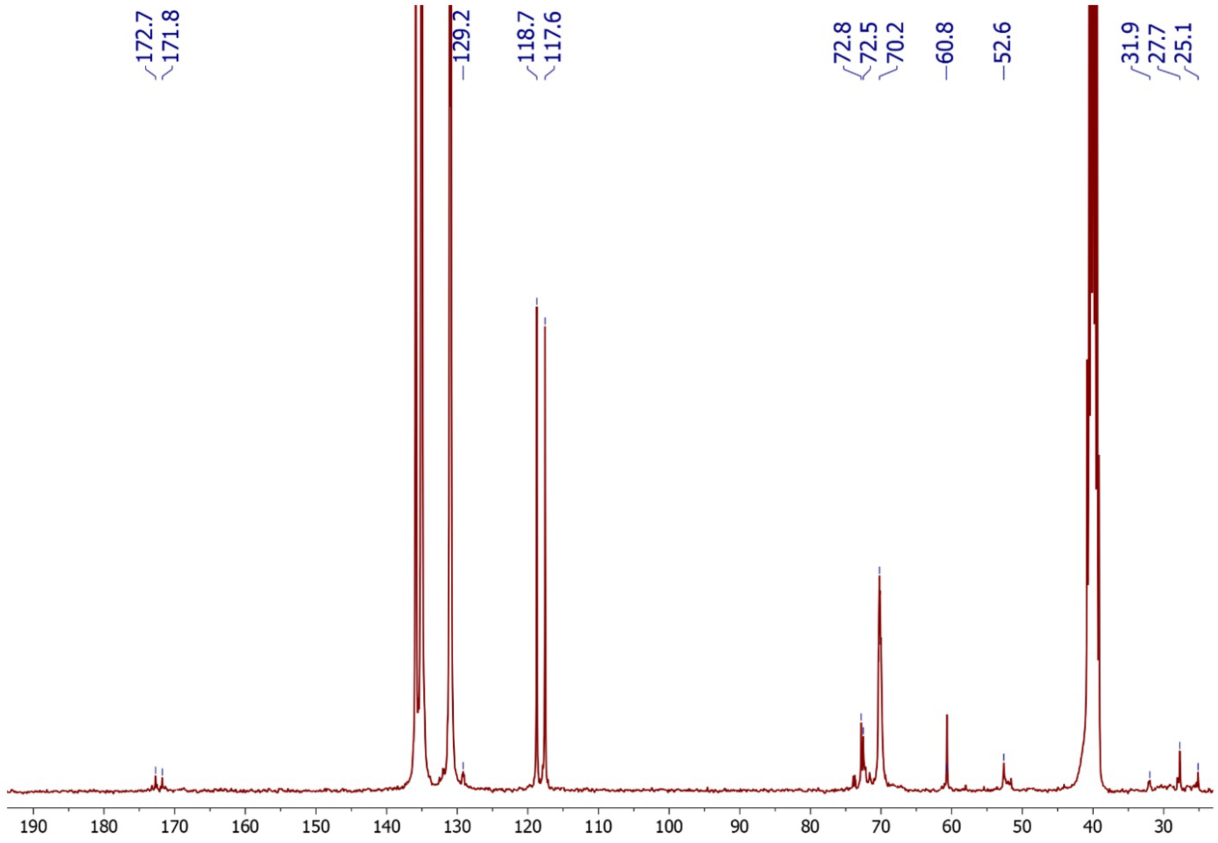

Fig. S23. ^{13}C NMR spectrum in DMSO-d_6.

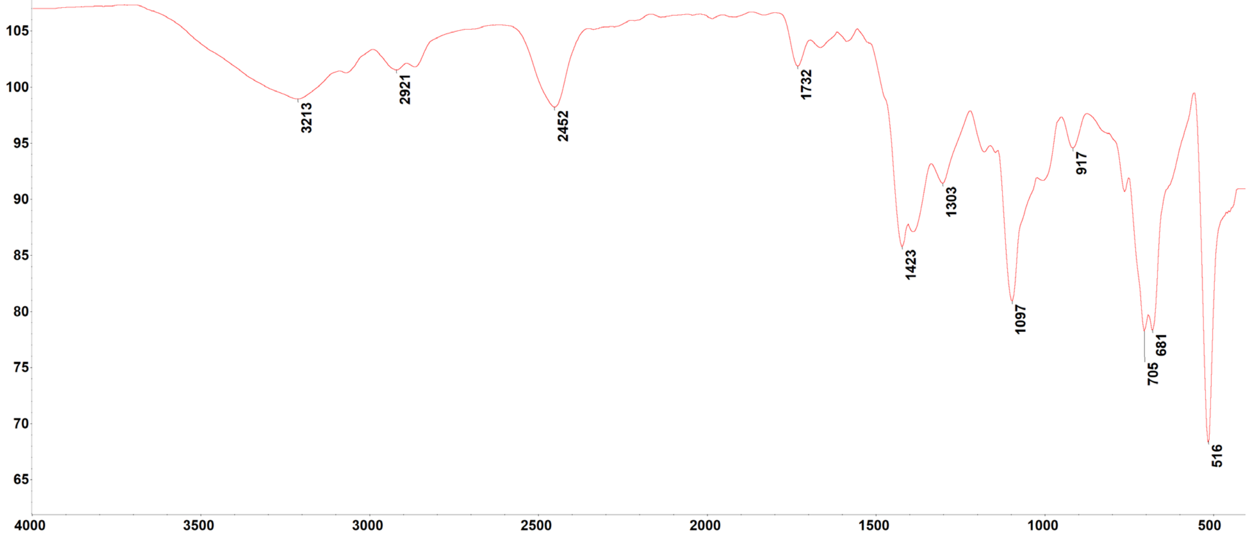

Fig. S24. IR spectrum.

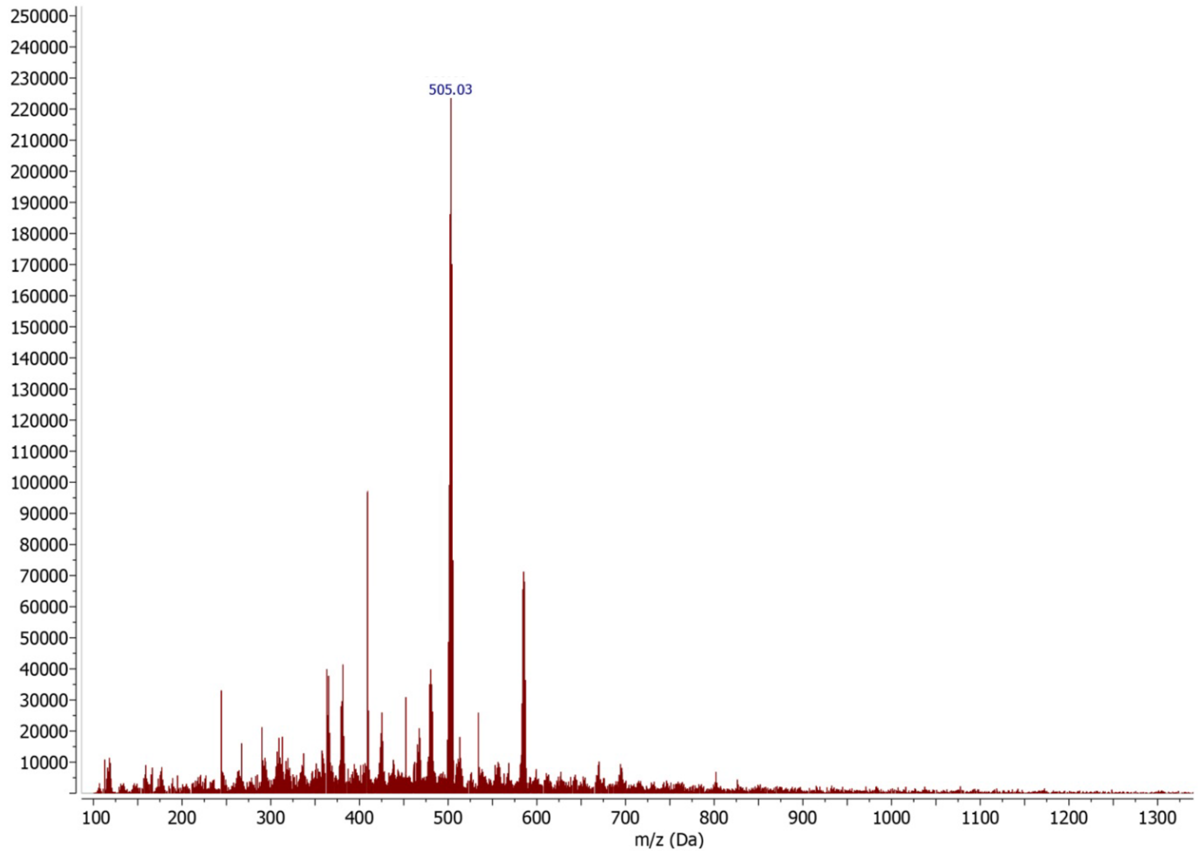

Fig. S25. Anionic part of the ESI mass-spectrum.

$(Ph_4P)_2[B_{10}H_9O(CH_2CH_2O)_4(CH_2)_3C(O)TrpOMe]$, $(Ph_4P)_2$**6**

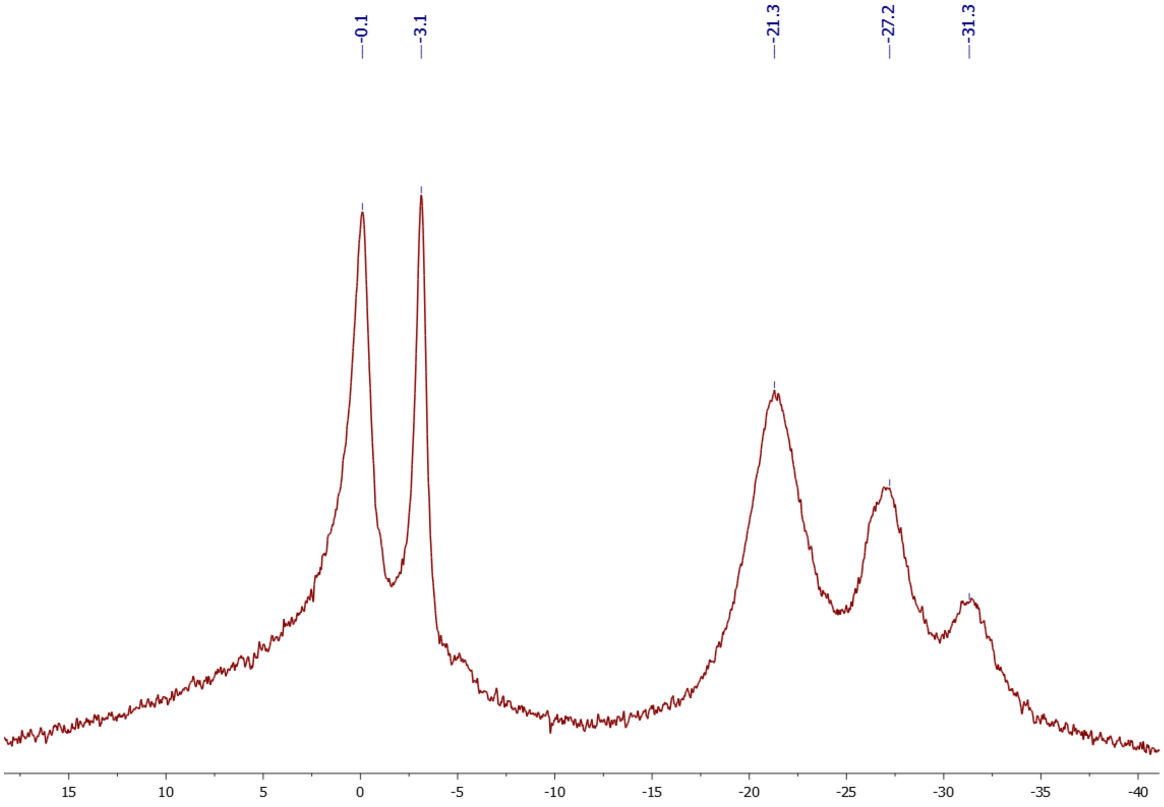

Fig. S26. $^{11}B\ \{^1H\}$ NMR spectrum in DMSO-d_6.

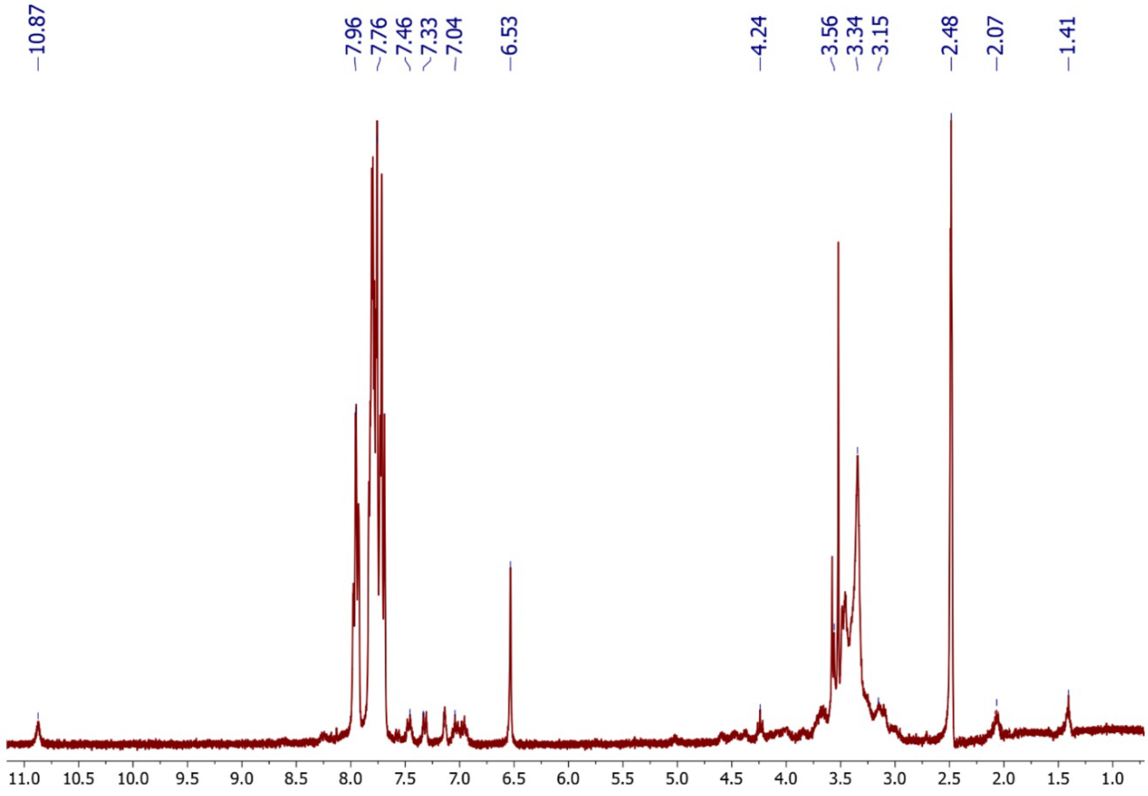

Fig. S27. ^1H NMR spectrum in DMSO-d_6.

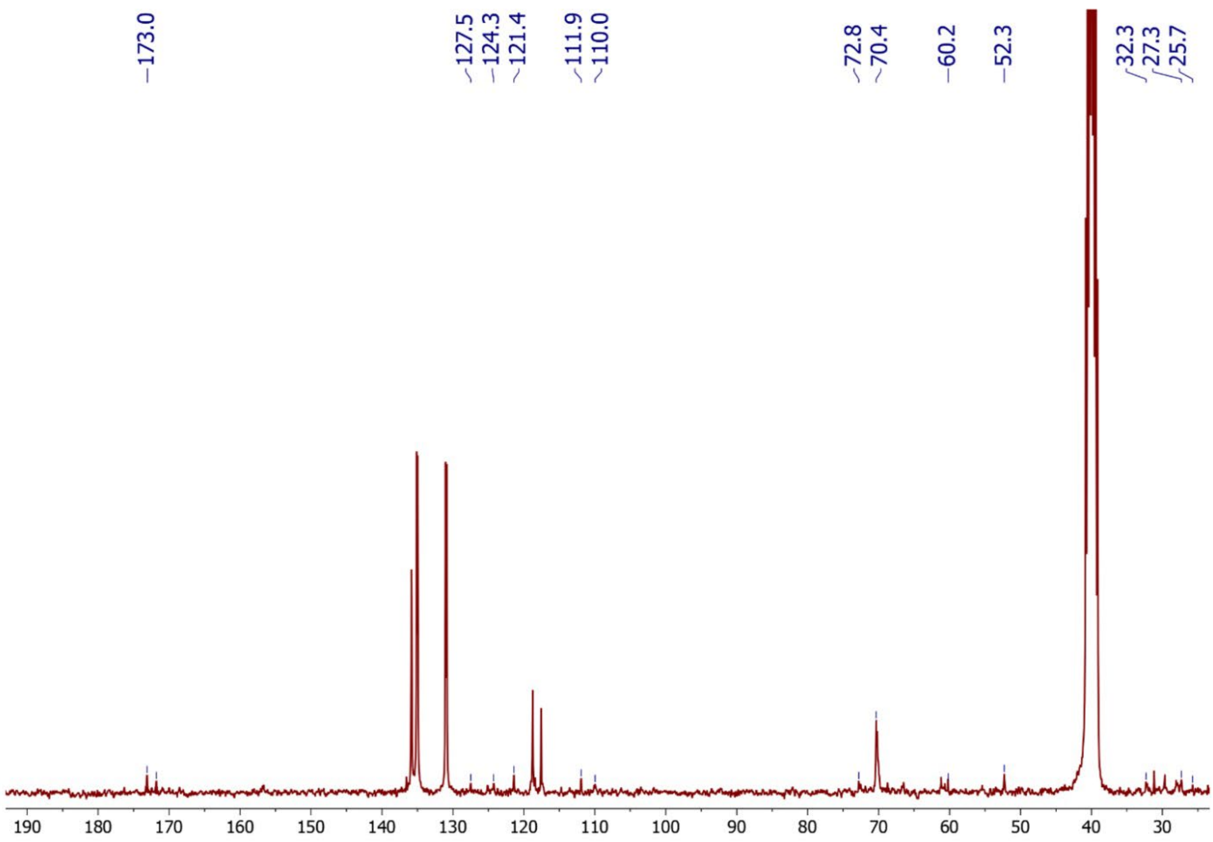

Fig. S28. ^{13}C NMR spectrum in DMSO-d_6.

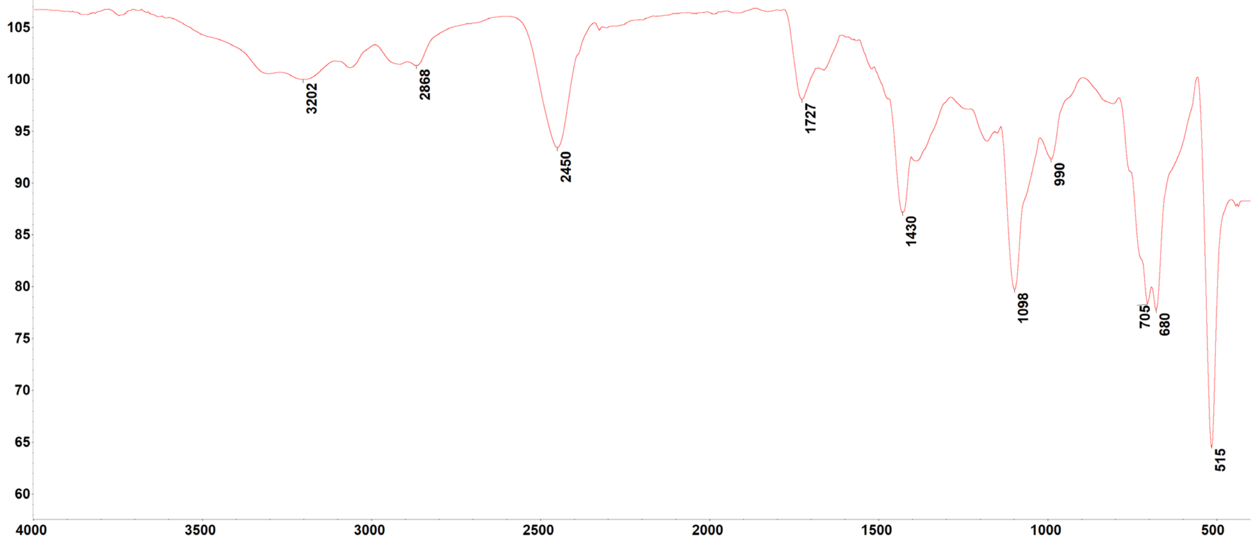

Fig. S29. IR spectrum.

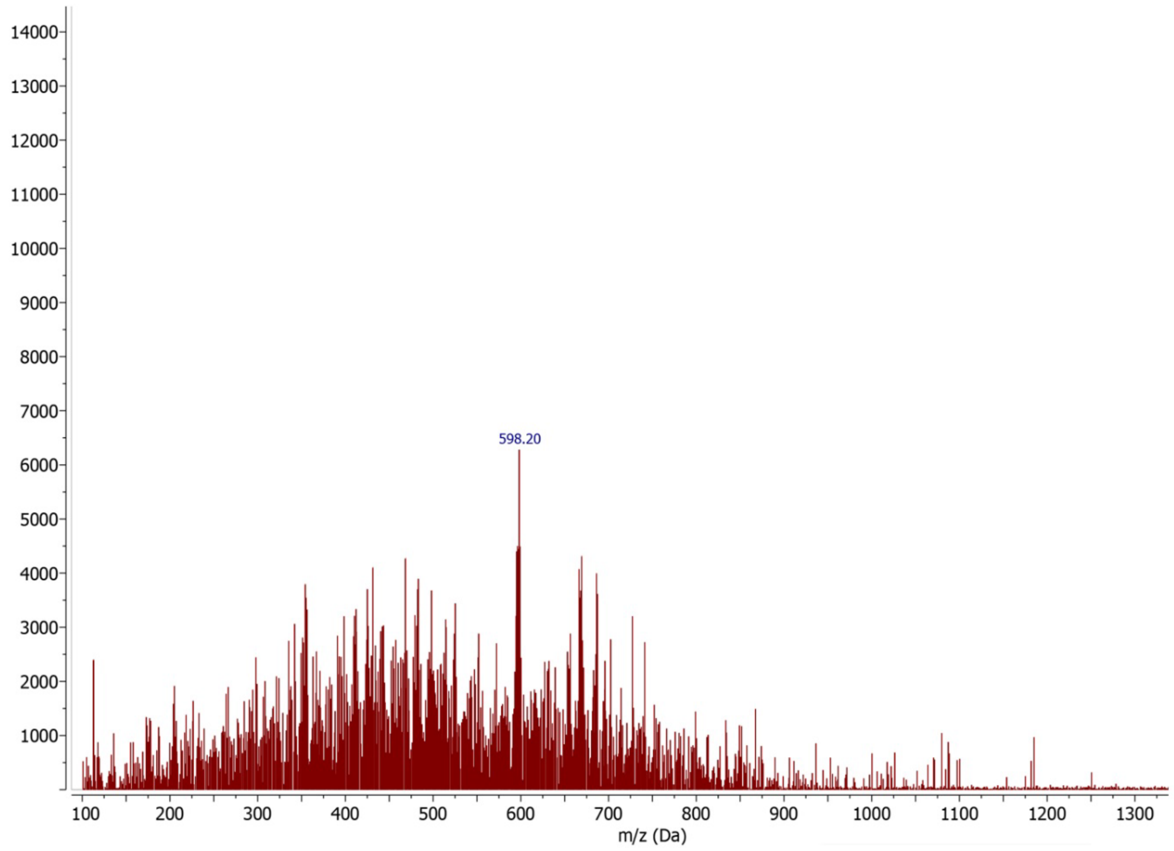

Fig. S30. Anionic part of the ESI mass-spectrum.

$(Ph_4P)_2[B_{10}H_9O(CH_2CH_2O)_4(CH_2)_3C(O)HisOMe]$, $(Ph_4P)_2$**7**

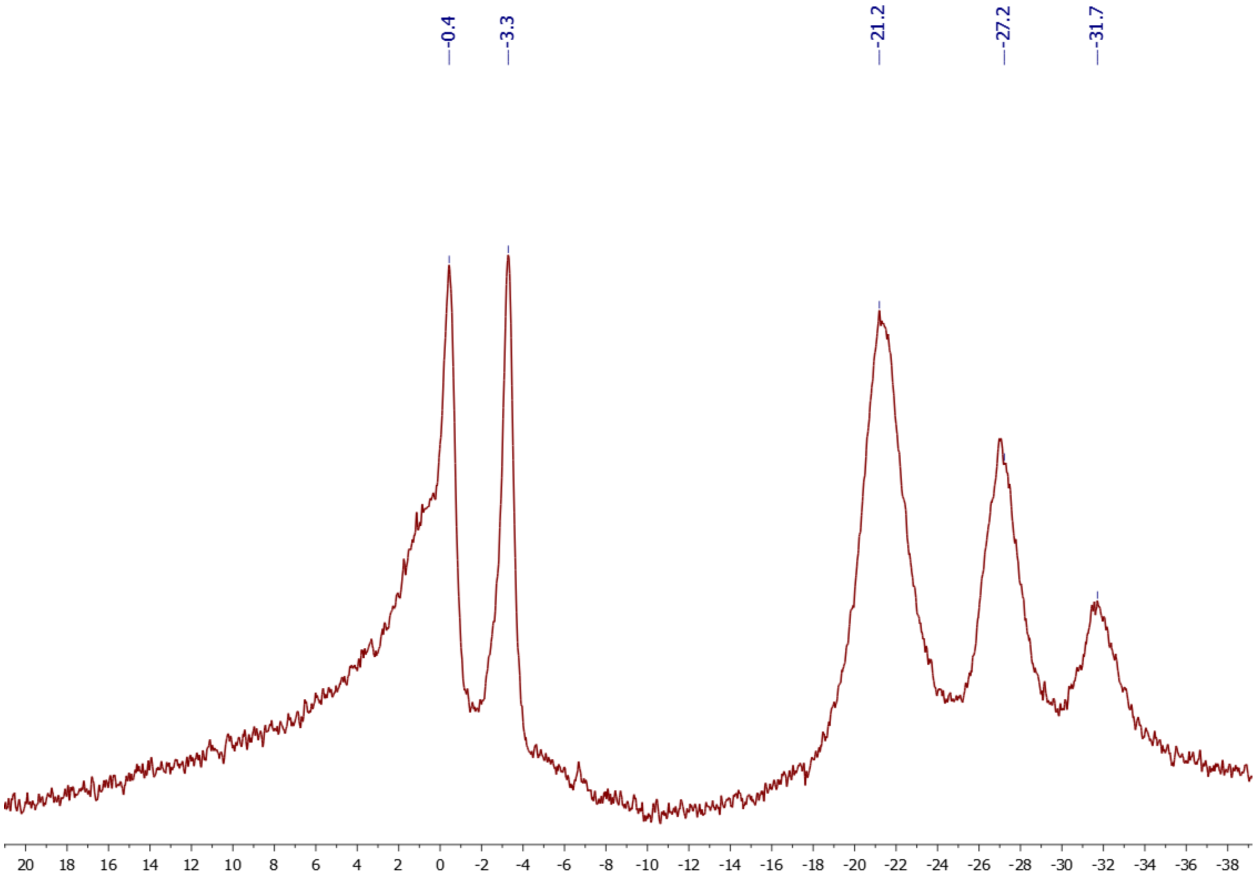

Fig. S31. $^{11}B\{^1H\}$ NMR spectrum in DMSO-d_6.

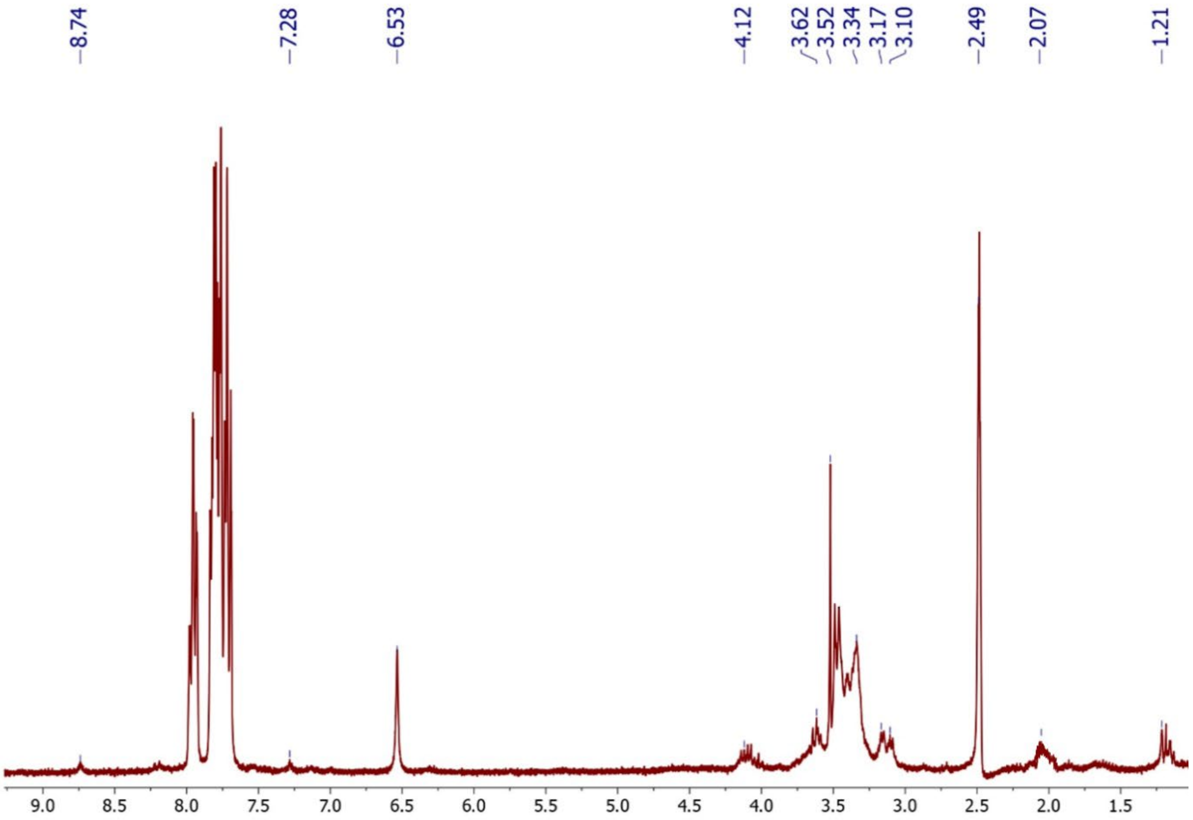

Fig. S32. ^{1}H NMR spectrum in DMSO-d$_6$.

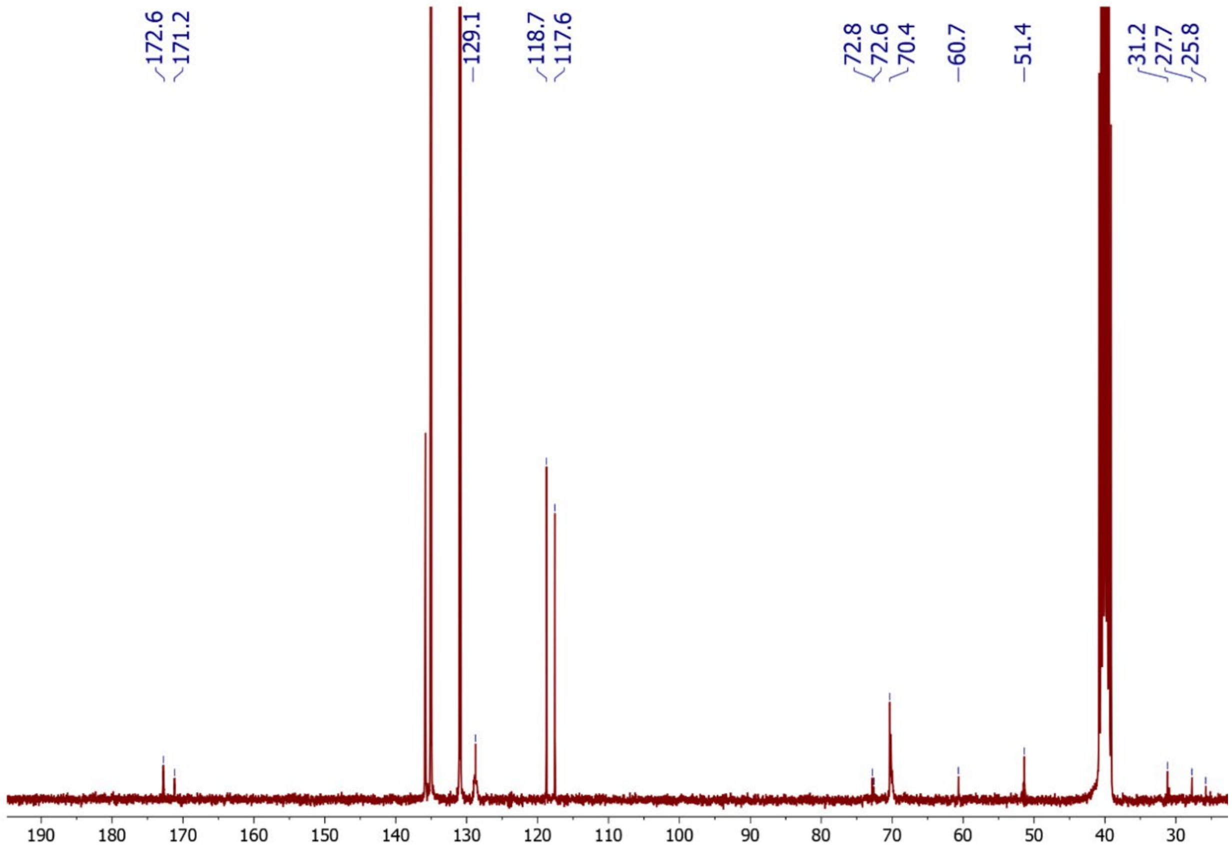

Fig. S33. ^{13}C NMR spectrum in DMSO-d$_6$.

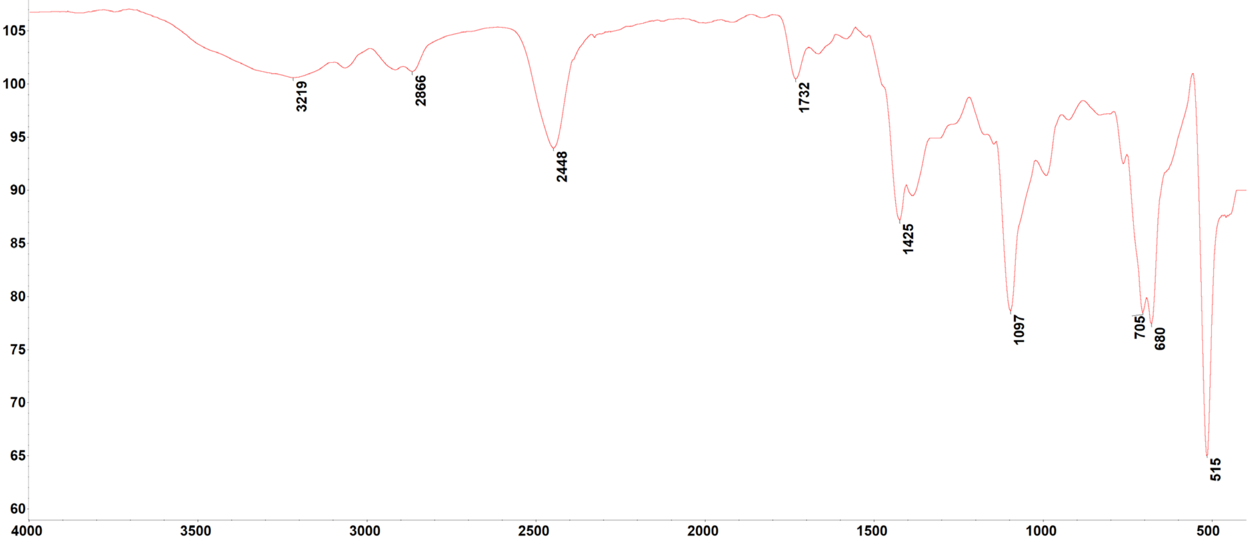

Fig. S34. IR spectrum.

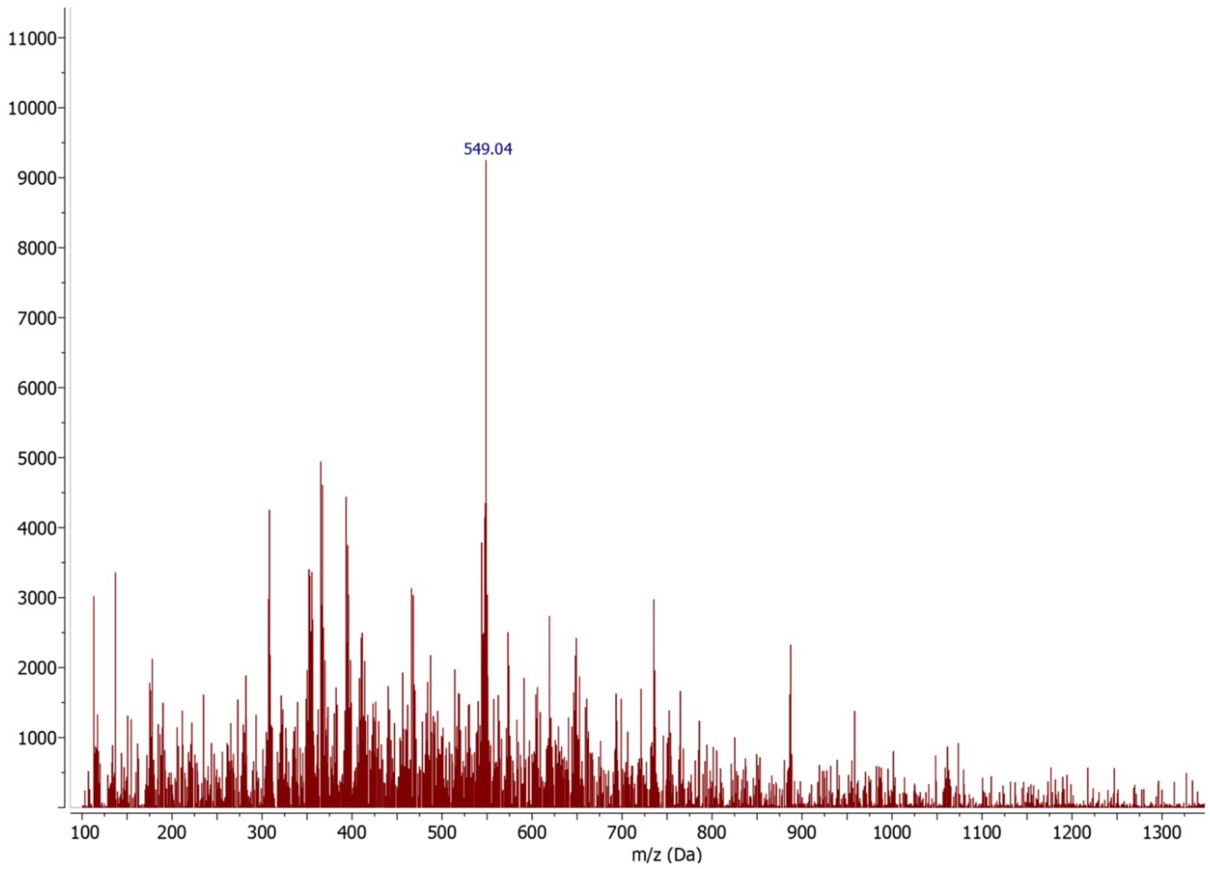

Fig. S35. Anionic part of the ESI mass-spectrum.

$(Ph_4P)_2[B_{10}H_9O(CH_2CH_2O)_5(CH_2)_3C(O)TrpOMe]$, $(Ph_4P)_2$**8**

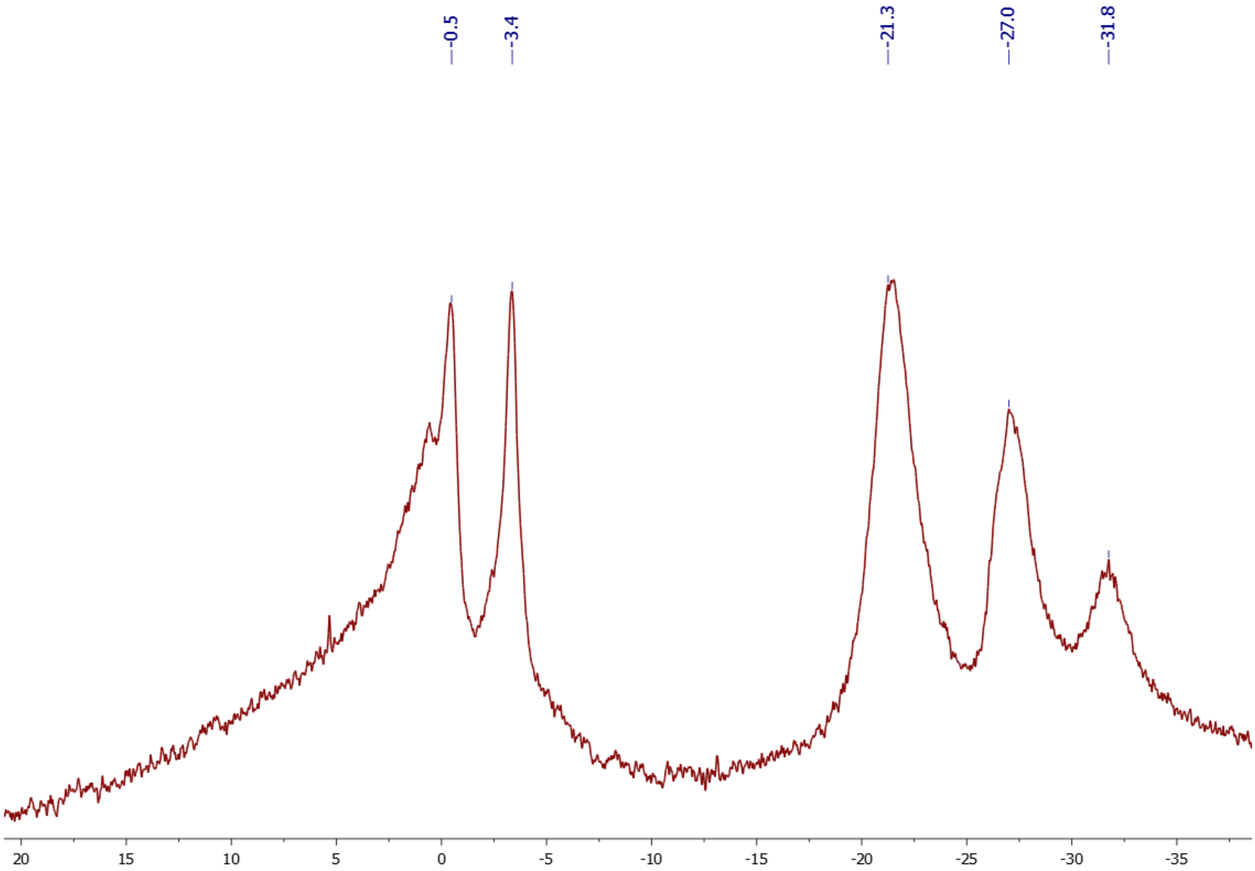

Fig. S36. $^{11}B\ \{^1H\}$ NMR spectrum in DMSO-d_6.

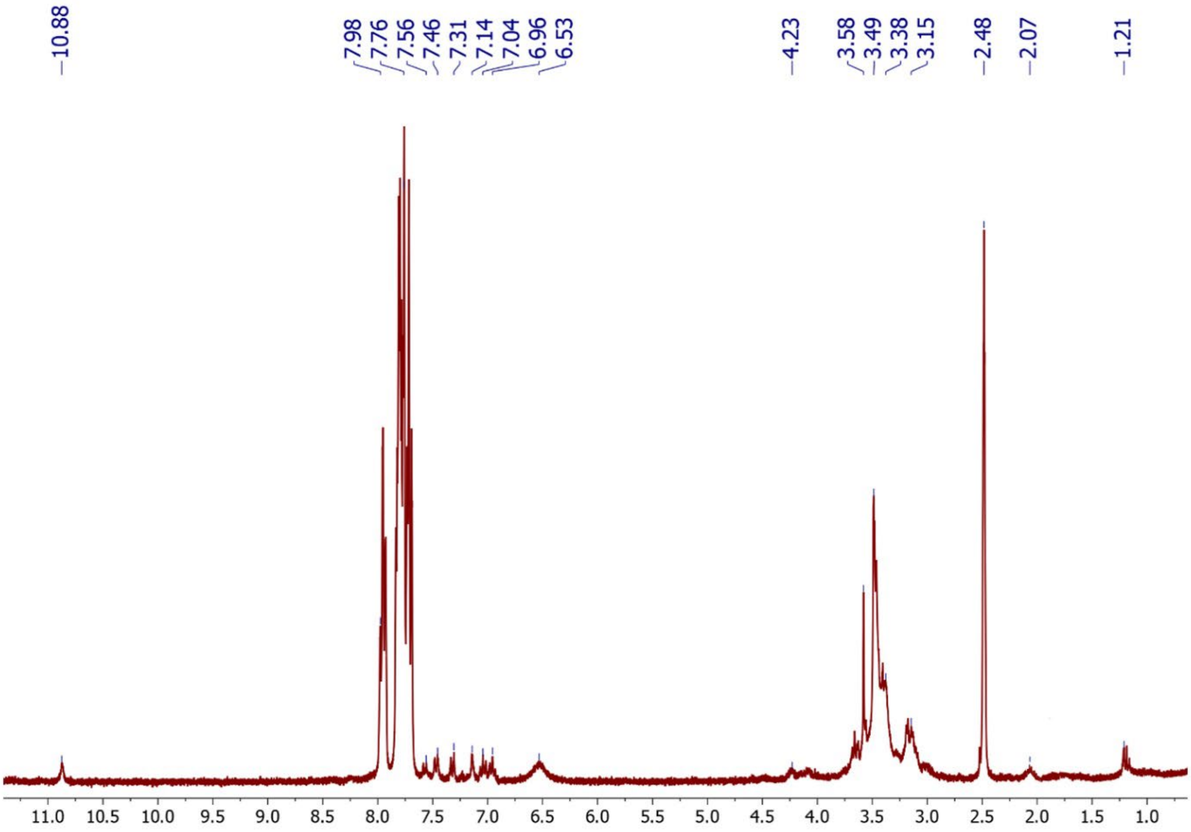

Fig. S37. ^1H NMR spectrum in DMSO-d_6.

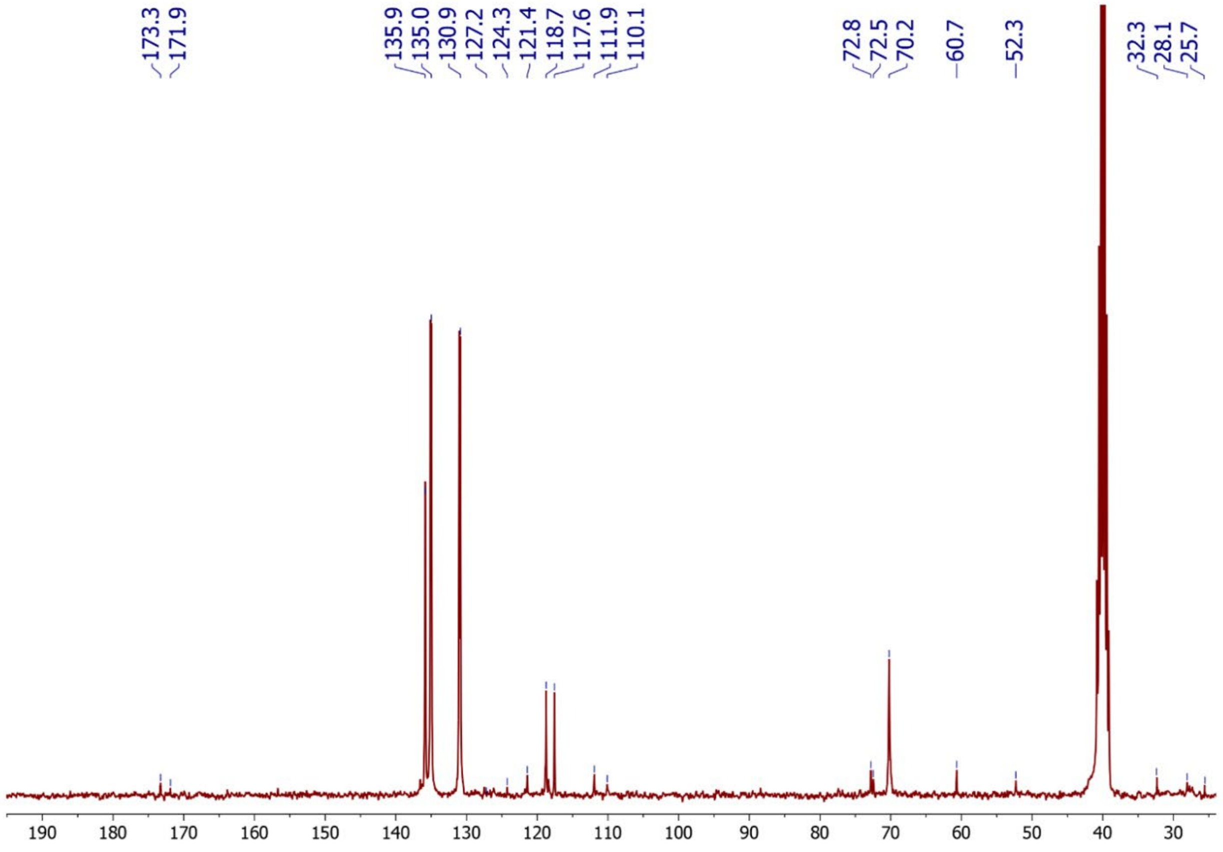

Fig. S38. ^{13}C NMR spectrum in DMSO-d_6.

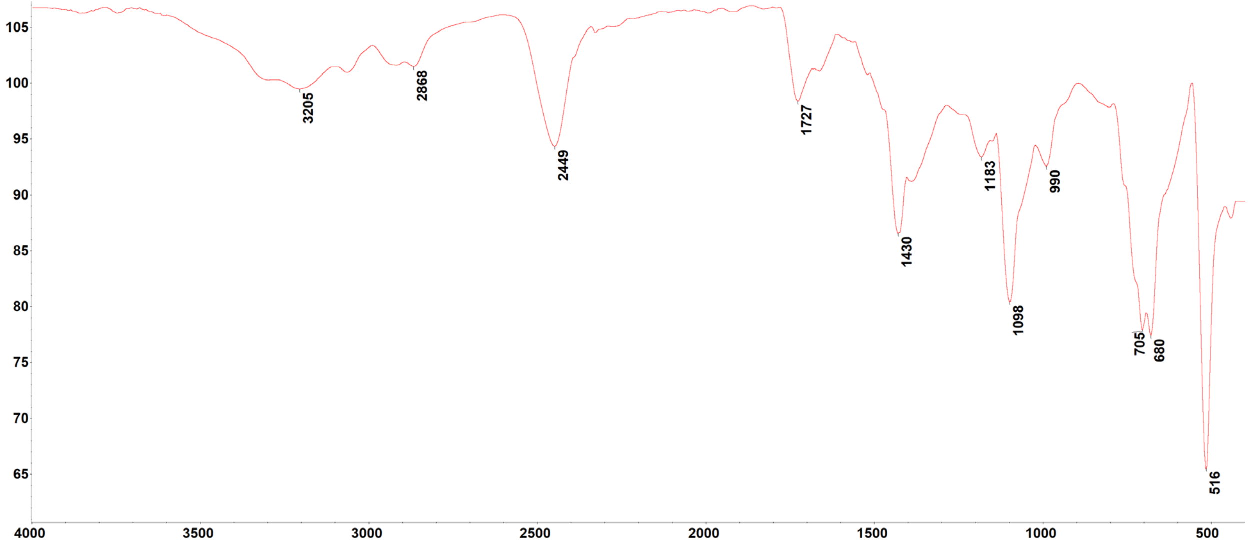

Fig. S39. IR spectrum.

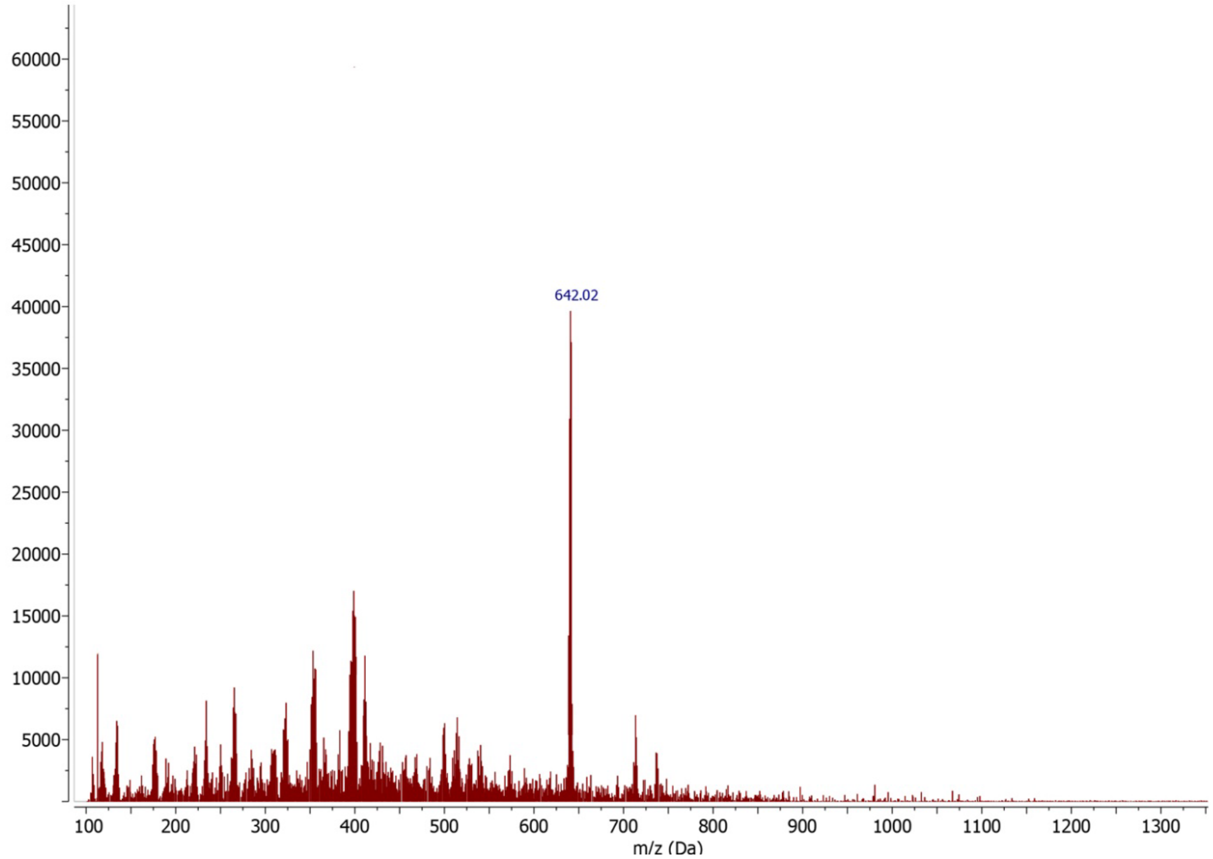

Fig. S40. Anionic part of the ESI mass-spectrum.

$(Ph_4P)_2[B_{10}H_9O(CH_2CH_2O)_5(CH_2)_3C(O)HisOMe]$, $(Ph_4P)_2$**9**

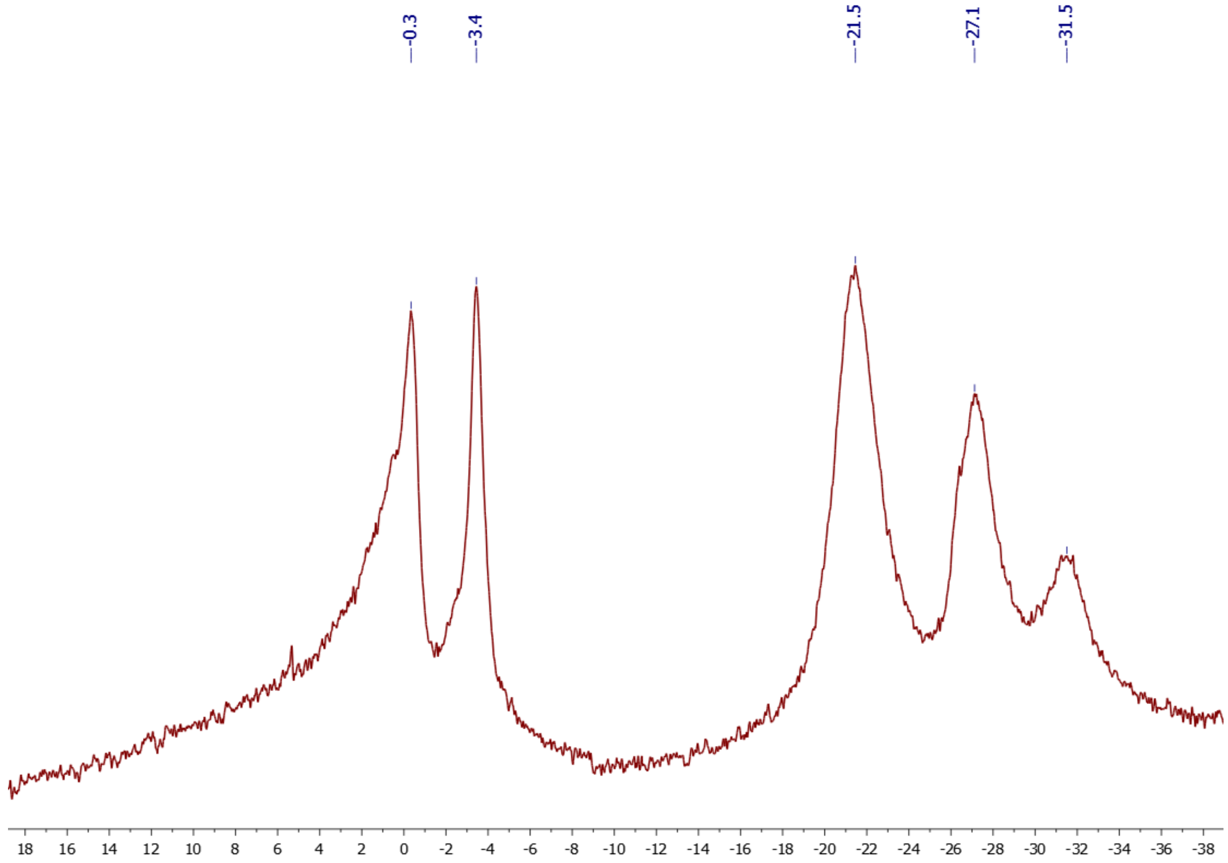

Fig. S41. $^{11}B\{^1H\}$ NMR spectrum in DMSO-d$_6$.

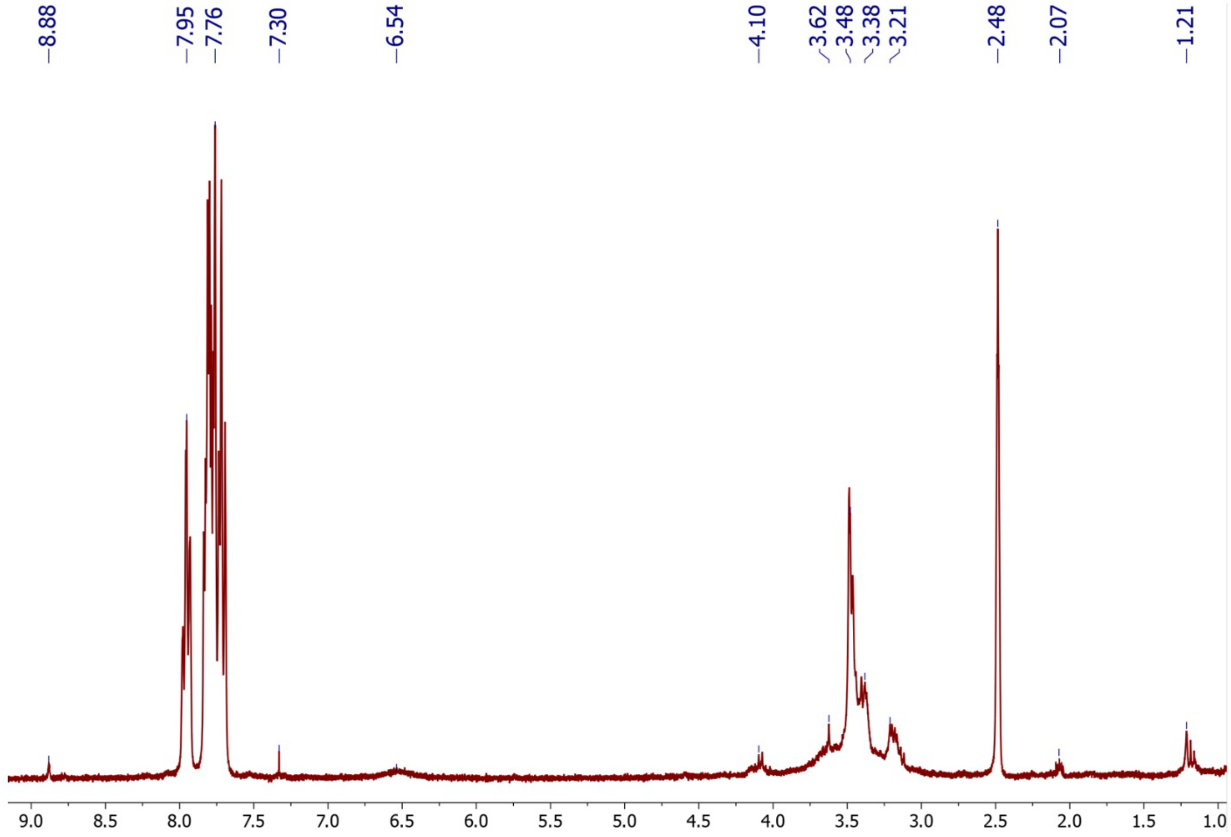

Fig. S42. ^1H NMR spectrum in DMSO-d$_6$.

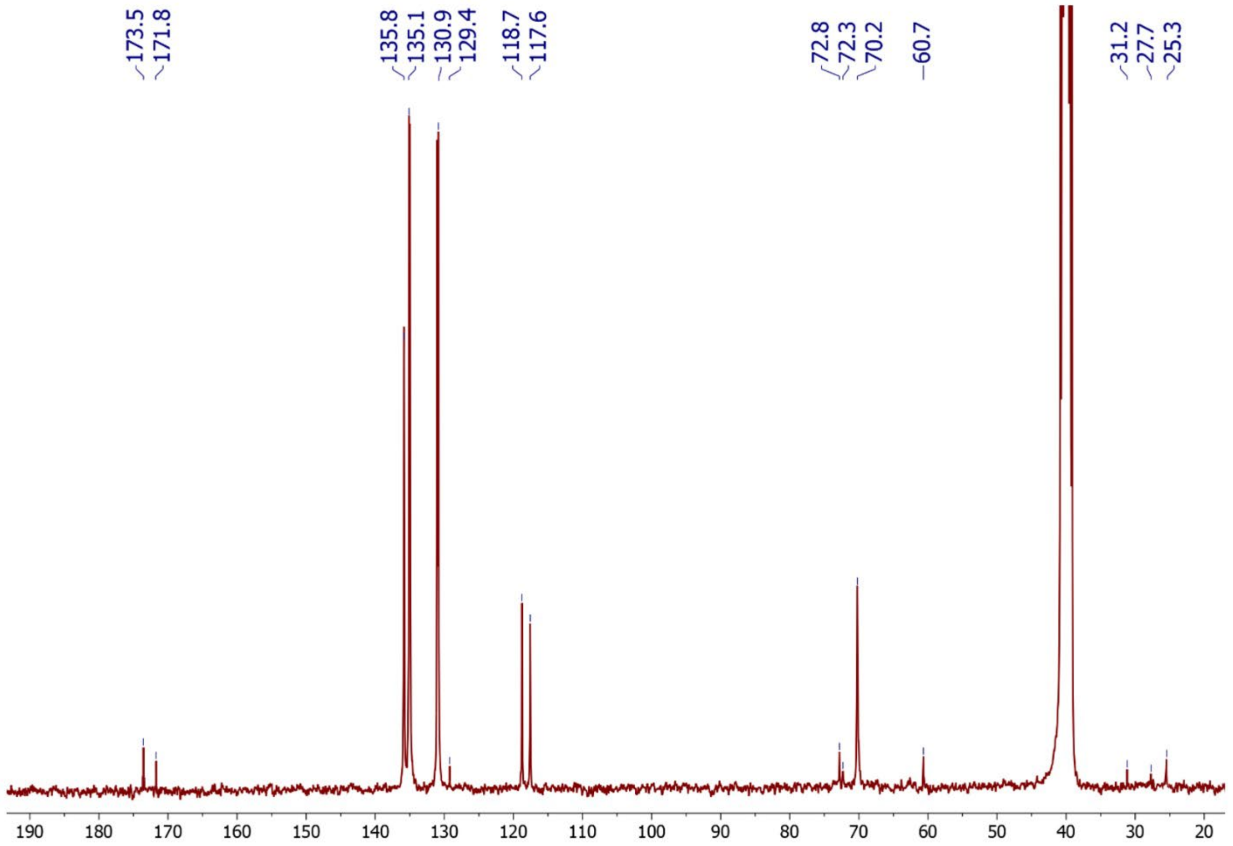

Fig. S43. ^{13}C NMR spectrum in DMSO-d_6.

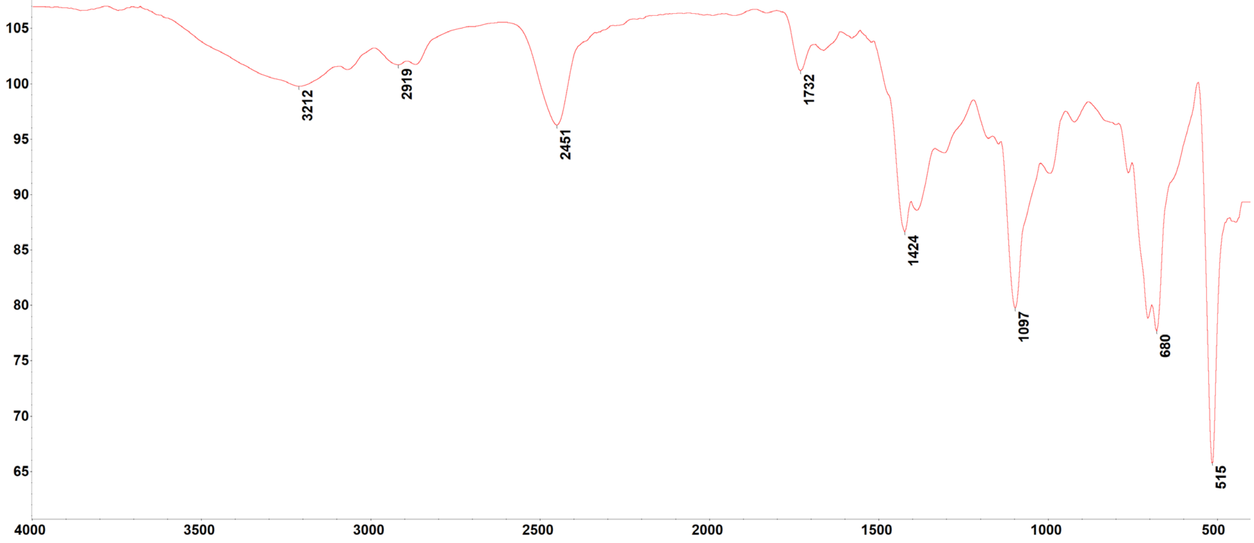

Fig. S44. IR spectrum.

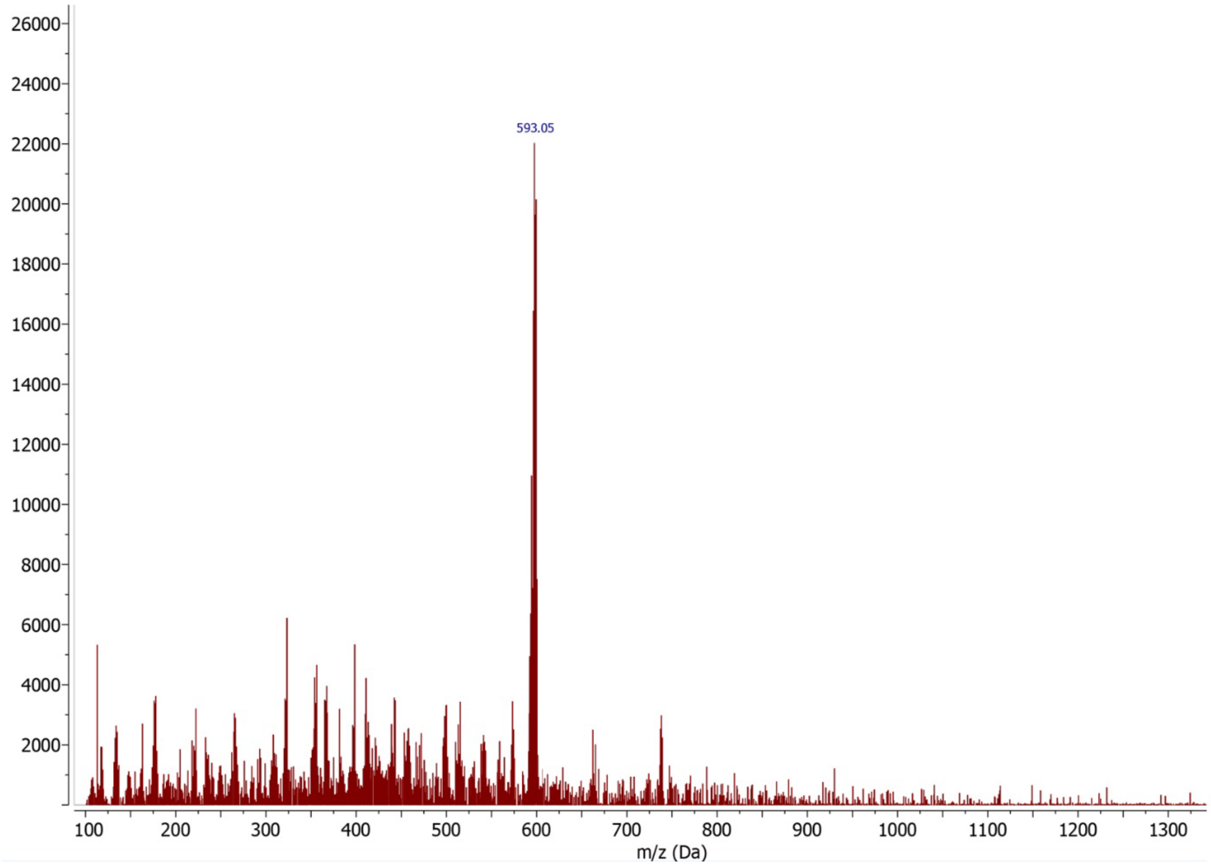

Fig. S45. Anionic part of the ESI mass-spectrum.

^{11}B NMR monitoring of the oxonium ring-opening in the derivatives $[B_{10}H_9O_4(CH_2)_8]^-$, $[B_{10}H_9O_5(CH_2)_{10}]^-$ and $[B_{10}H_9O_6(CH_2)_{12}]^-$ with malonic ester in the presence of K_2CO_3

1) Stirring at room temperature for 12 h

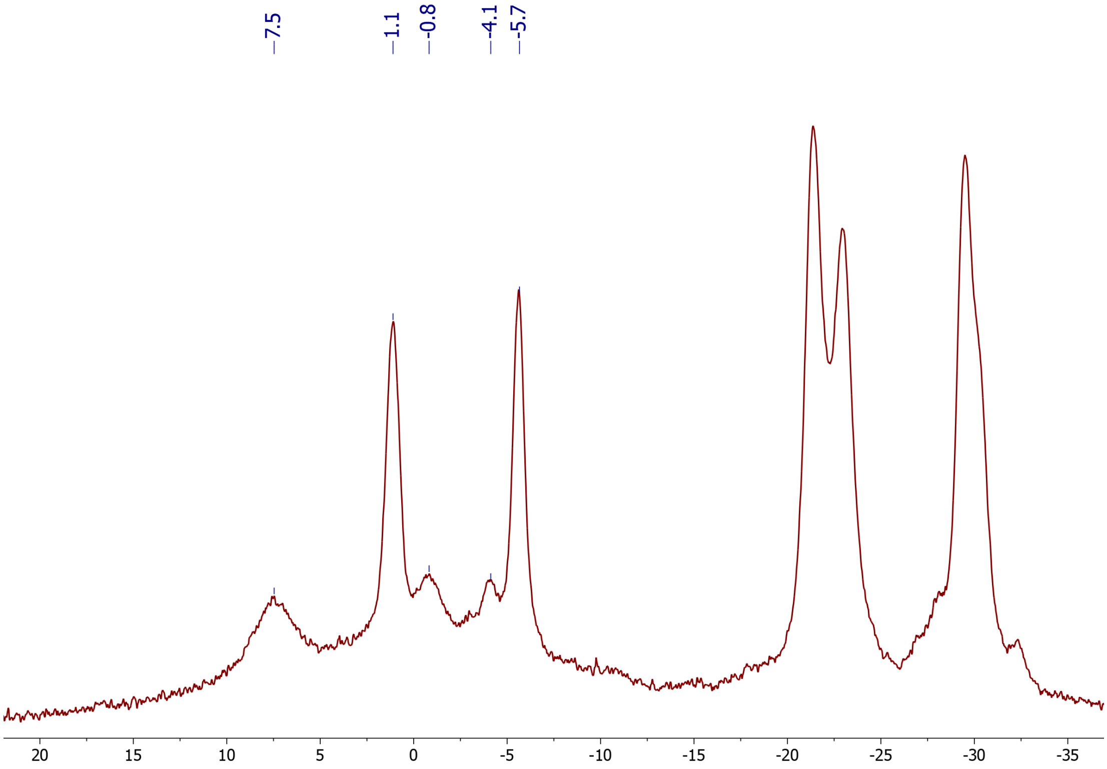

Reaction with $[2\text{-}B_{10}H_9O_4(CH_2)_8]^-$

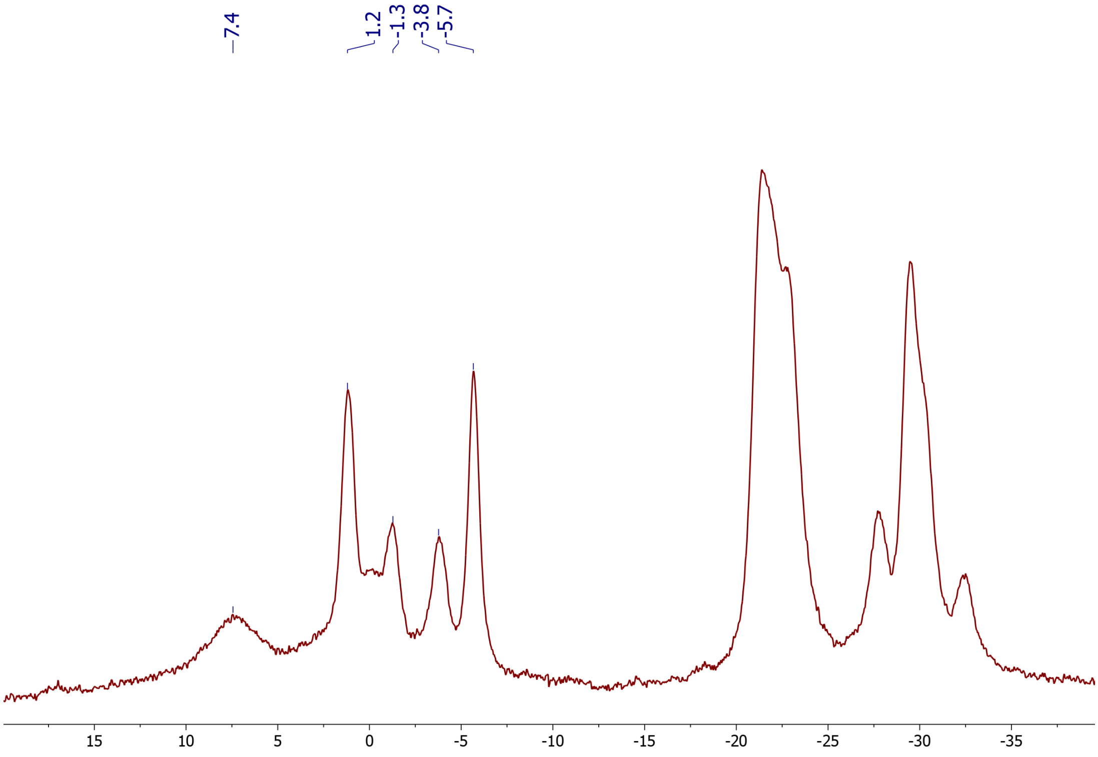

Reaction with $[2\text{-}B_{10}H_9O_5(CH_2)_{10}]^-$

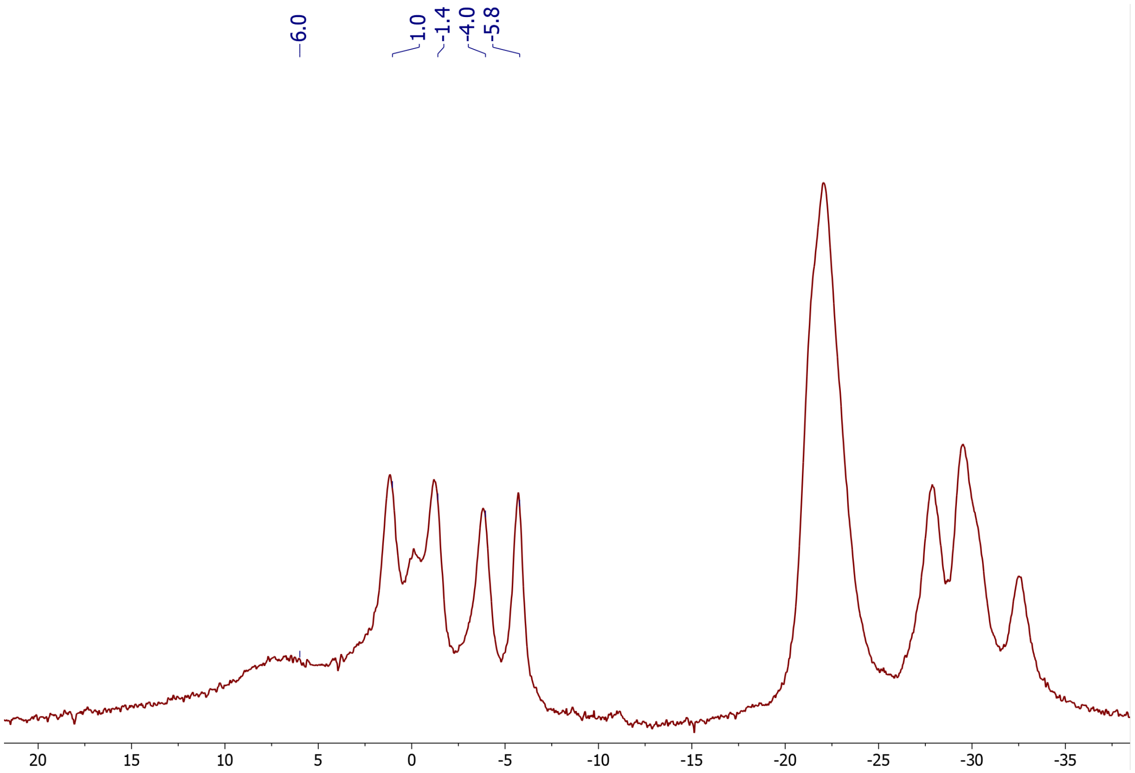

Reaction with $[2\text{-}B_{10}H_9O_6(CH_2)_{12}]^-$

2) Refluxing for 2 h

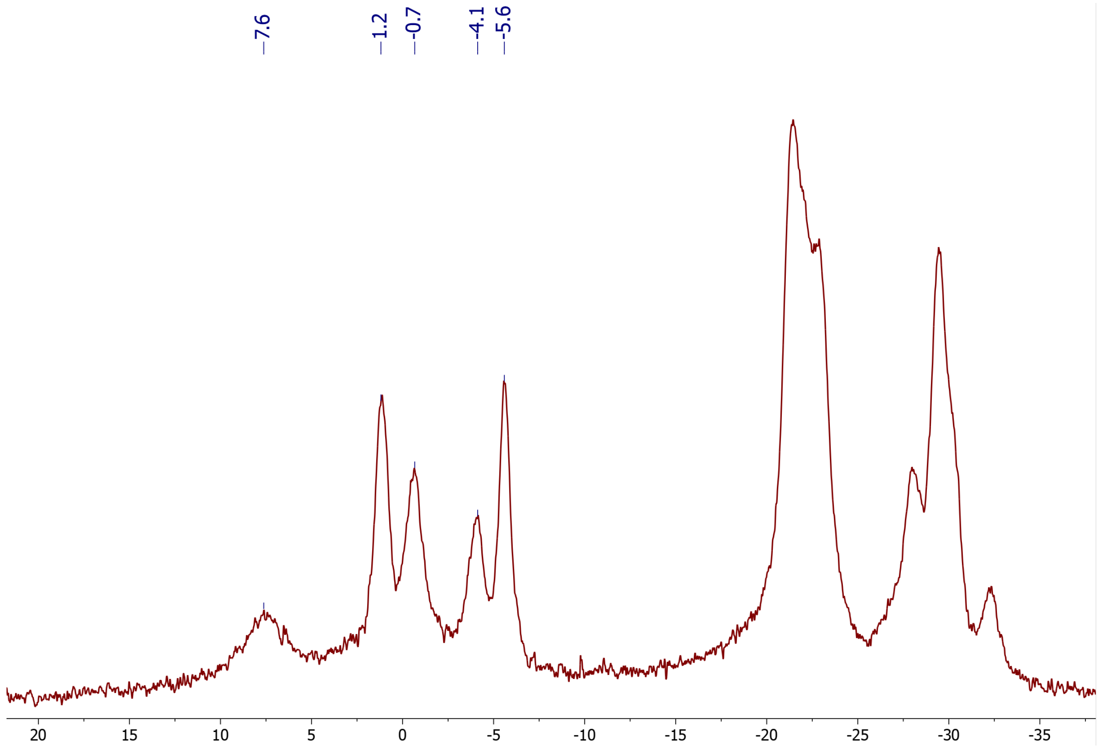

Reaction with $[2\text{-}B_{10}H_9O_4(CH_2)_8]^-$

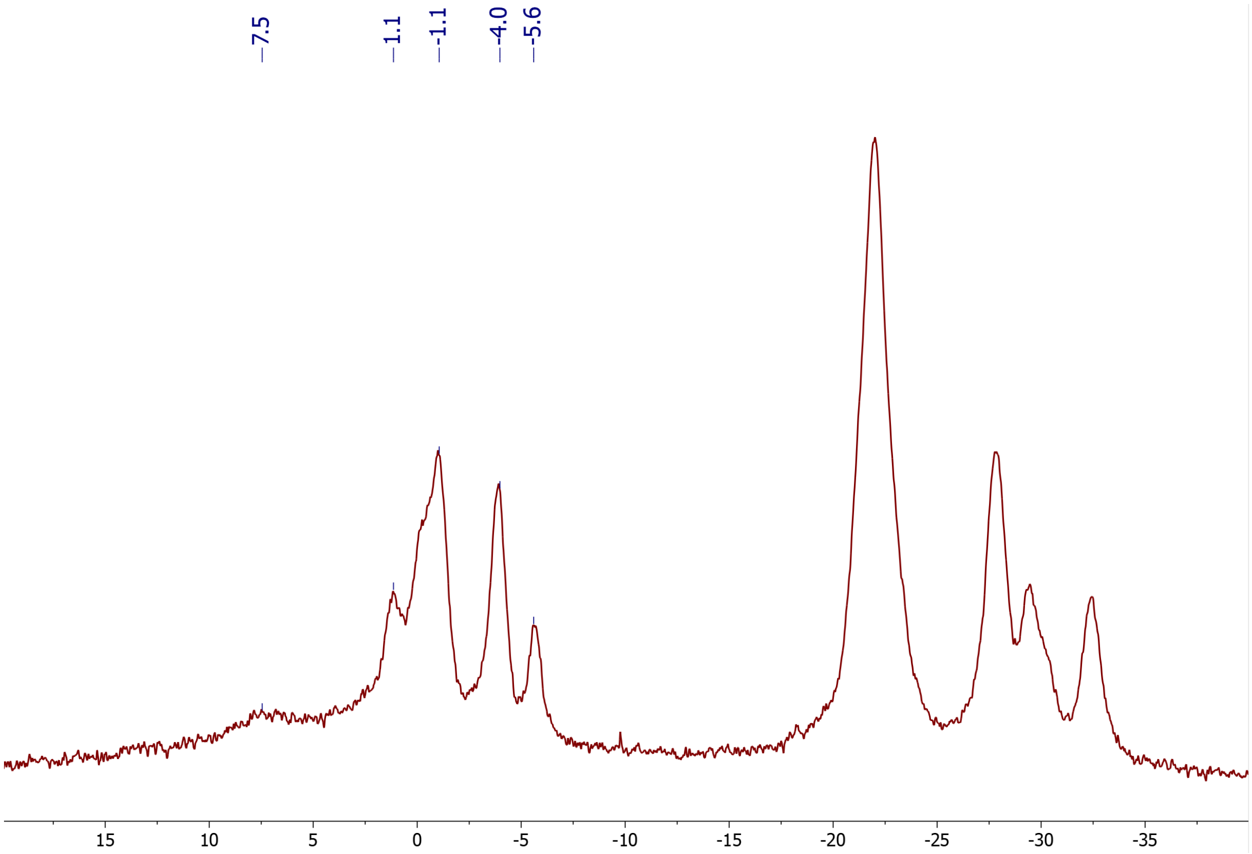

Reaction with $[2\text{-}B_{10}H_9O_5(CH_2)_{10}]^-$

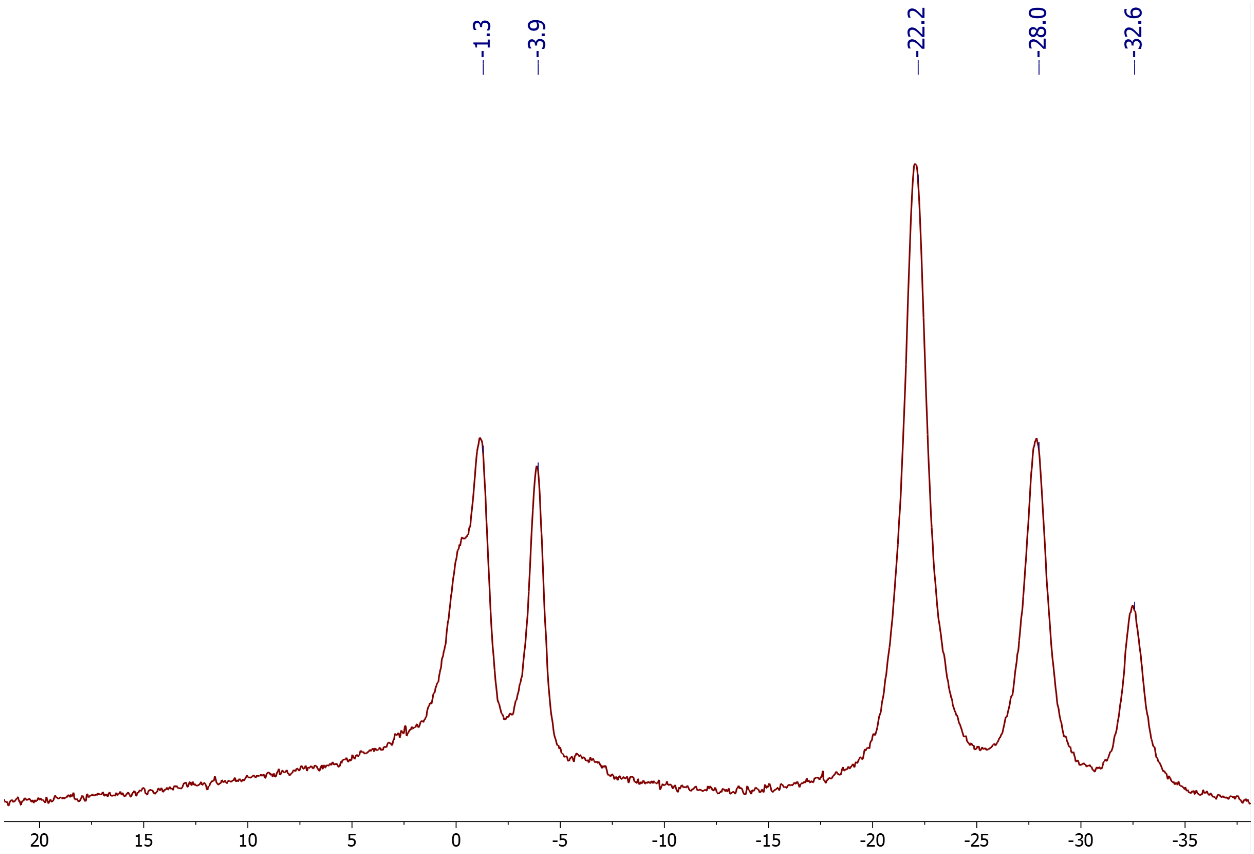

Reaction with $[2\text{-}B_{10}H_9O_6(CH_2)_{12}]^-$

3) Refluxing for 8 h

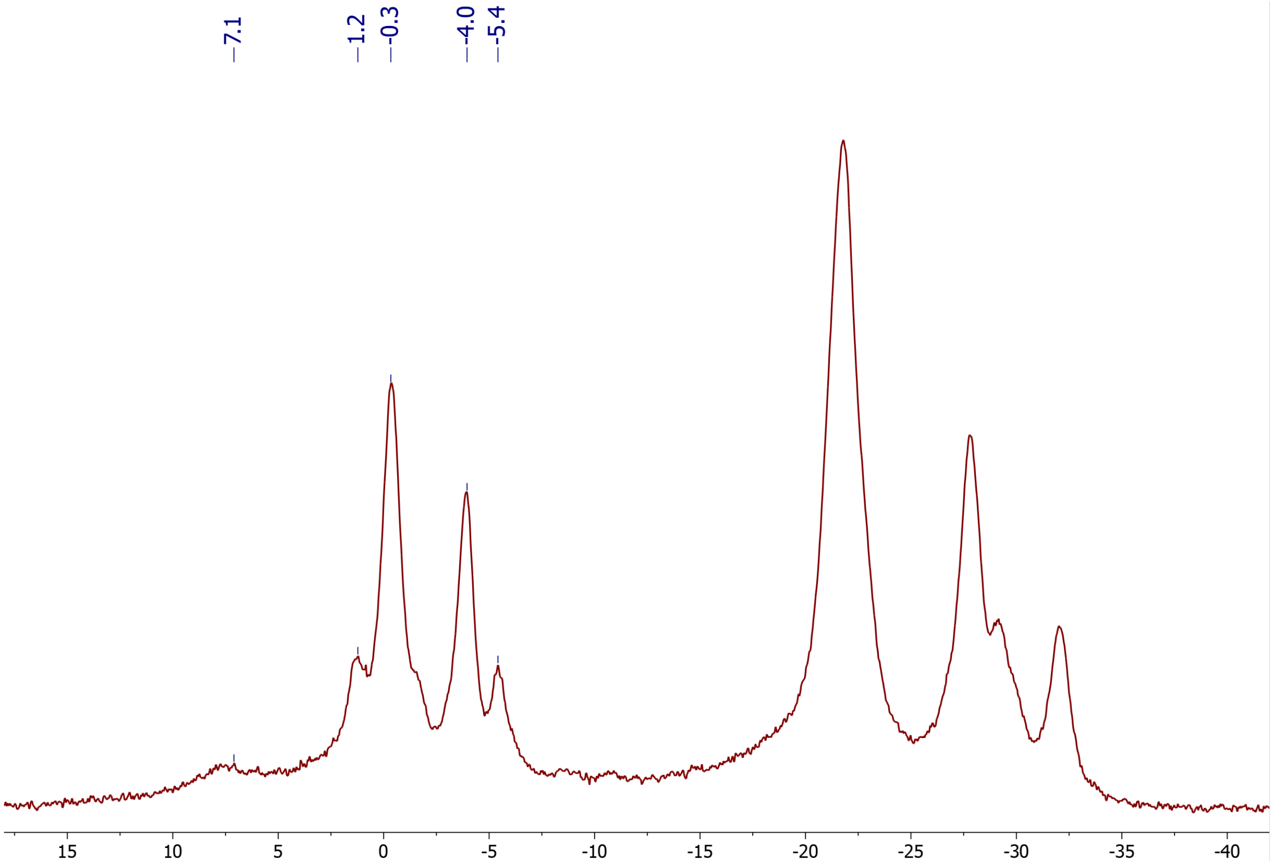

Reaction with $[2\text{-}B_{10}H_9O_4(CH_2)_8]^-$

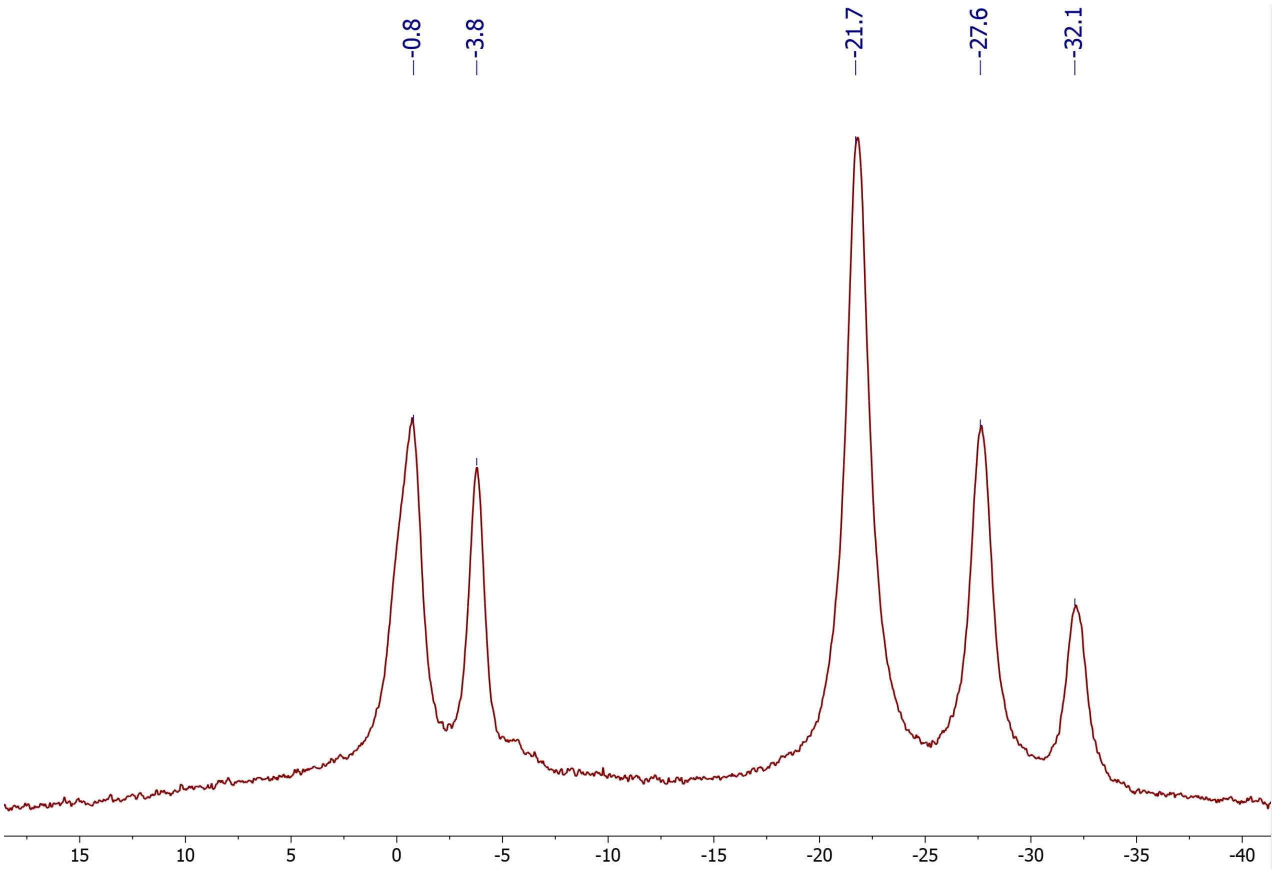

Reaction with $[2\text{-}B_{10}H_9O_5(CH_2)_{10}]^-$

4) Refluxing for 16 h

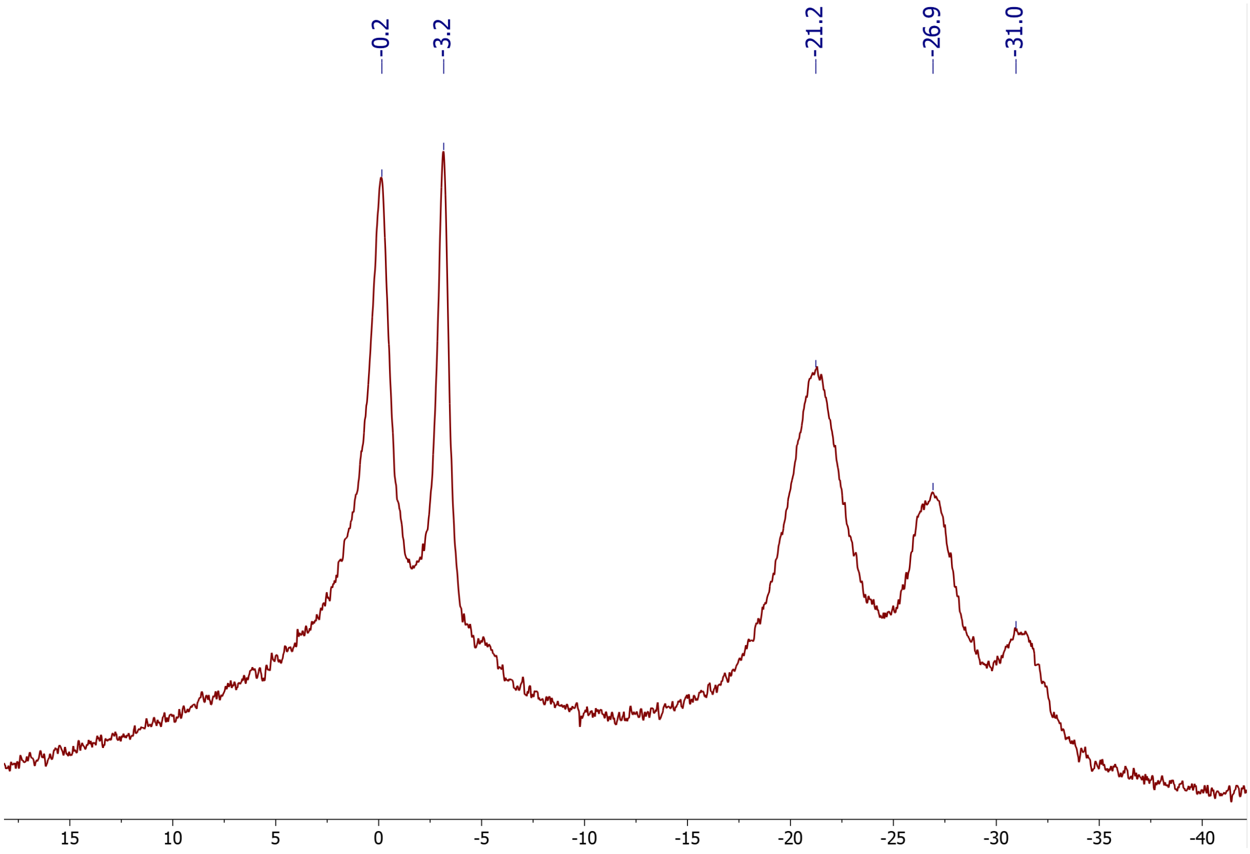

Reaction with $[2\text{-}B_{10}H_9O_4(CH_2)_8]^-$

Details of biological experiments

Study of antiviral activity of synthetic compounds to influenza A virus by enzyme-linked immunosorbent assay (ELISA)

MDCK cells were grown in 96-well panels to a full monolayer and before adding drugs and viruses, washed twice with serum-free medium to reduce possible non-specific reactions. The drugs or compounds under study were added to the cells at a 2-fold concentration in 100 µl of MEM medium. 100 µl of the same medium were added to the viral control, and 200 µl to the cell control. The panels were incubated at 37°C with CO_2. When determining the effect of antiviral drugs on the expression of viral antigens, 4 dilutions of the virus (at a working dose of 10-10,000 $TCID_{50}$) were added to the wells of the cells with the drugs under study, excluding the cell control, taking into account the dilution with the drug in a volume of 100 µl. All procedures were performed on MEM medium with the addition of trypsin (TPCK treated, Sigma, USA) at a concentration of 2 µg/ml. The panels were incubated in a thermostat with CO2 for 24 hours at 37°C. After incubation, the medium was removed and the cells were fixed with 80% acetone in phosphate-buffered saline (PBS) for 15-20 minutes in a volume of 100 µl, then well dried and washed 3 times with a PBS solution with 0.05% TWEEN 20 in a volume of 200 µl. All further washing procedures were carried out with the specified solution. Then 100 µl of ELISA buffer (phosphate-buffered saline with 1% fetal serum and 0.05% TWEEN 20) were added to the cells and incubated at 37°C for 30 min. After removing the solution, 100 µl of monoclonal antibodies (mAbs) to the internal M and NP proteins of the influenza A virus were added to the cells at a dilution of 1:1000 in ELISA buffer. After incubation with antibodies for 1 hour at 37^0C and subsequent 2-fold washing, 100 µl of rabbit IgG against mouse IgG labeled with horseradish peroxidase at a dilution of 1:5000 in ELISA solution were added to the wells and incubated for 1 hour at 37°C. After washing three times, doped peroxidase was detected by adding 100 µl of 3,3',5,5'-tetramethylbenzidine (TMB) in 0.003% citrate buffer pH 5.0 containing 0.003% H_2O_2 (substrate) to the wells. The panels were kept in the dark for 15 minutes until color appeared, the reaction was stopped by adding 50 µl of 1N H_2SO_4 to each well.

Then the optical density (OD) was measured on a Scientific Multiskan GO automatic spectrophotometer (Thermo Fisher, USA) at a wavelength of 450 nm. Wells not infected with the virus were used as a control. The percentage of inhibition of viral reproduction by the test compound was determined by the formula: percentage of inhibition = 100 – (OD of the experiment – OD of the cell control / OD of the viral control in the absence of the compound – OD of the cell control) x 100. Three wells of the panel were used for one experimental point, and each value represents the arithmetic mean ± standard deviation calculated from three such independent experiments.

The concentration of the drug or compound that reduces the OD value by 50% was taken as the minimum inhibitory concentration (IC_{50}). The minimum concentration that suppresses viral activity by 50% was calculated by graphical transfer of the results. The breadth of the pharmacological effect of the compounds was assessed by the chemotherapeutic index (CTI), which was defined as the ratio of the maximum tolerated concentration of the compound to its minimum active concentration: CTI = IC_{50}/ CC_{50}.

Determination of cytotoxic effect (MTT test)

The cytotoxicity of compounds Na21 and Na22 was assessed by adding different concentrations of the compounds to a monolayer of MDCK tissue culture cells in 96-well plates and incubating at 37°C. Initially, the cell monolayer was examined under a microscope. The concentration that caused degeneration of 50% of the cells compared to the control was recorded as the average toxic concentration (CC_{50}).

Additionally, cytotoxicity was evaluated using a colorimetric assay to measure cell metabolic activity with the dye 3-(4,5-dimethylthiazol-2-yl)-2,5-diphenyl-tetrazolium bromide (MTT). The test compound was added to the cells at concentrations of 40.0, 80.0, 160.0, 320.0, and 640.0 μg/mL (8 wells per concentration) in Eagle's MEM nutrient medium with L-glutamine and a double set of amino acids. The plates were incubated in a CO_2 incubator at 37°C for 48 h, with daily visual inspections under an optical microscope. Following incubation, the medium was removed, and 20.0 μL of MTT solution (5.0 mg/mL, 100 μg of MTT) was added to each well. The plates were further incubated at 37°C for 2-4 h, during which dark blue formazan crystals formed in the cells. After incubation, the medium was removed using a multichannel pipette, revealing a blue coating of formazan crystals on the bottom of the wells. To dissolve the formazan crystals, 150.0 μL of dimethyl sulfoxide (DMSO) was added to each well, and the plate was shaken for 5 min.

The optical density (OD) of the solutions in each well was recorded using an automatic spectrophotometer at a wavelength of 490 nm. The cell viability was assessed by comparing the OD of the experimental wells with different drug concentrations with the OD of the control wells (cells without the drug).
